# TNFSF13 insufficiency disrupts human colonic epithelial cell growth and associated B cell dynamics

**DOI:** 10.1172/JCI186032

**Published:** 2026-04-01

**Authors:** Xianghui Ma, Shaneice K. Nettleford, Yuhua Tian, Noor Dawany, Ayano Kondo, Yalan Li, Kelly Maurer, Tatiana A. Karakasheva, Rawan Shraim, Patrick A. Williams, Louis R. Parham, Lauren A. Simon, Charles H. Danan, Maire A. Conrad, David A. Piccoli, Marcella Devoto, Neil Romberg, Kathleen E. Sullivan, Klaus H. Kaestner, Judith R. Kelsen, Kathryn E. Hamilton

**Affiliations:** 1 Division of Gastroenterology, Hepatology, and Nutrition, Department of Pediatrics, Children’s Hospital of Philadelphia, Philadelphia, Pennsylvania, USA.; 2 State Key Laboratory of Metabolic Dysregulation & Prevention and Treatment of Esophageal Cancer, Tianjian Laboratory of Advanced Biomedical Sciences, School of Convergence Medicine, Zhengzhou University, Zhengzhou, Henan, China.; 3 Department of Biomedical and Health Informatics, Children’s Hospital of Philadelphia, Philadelphia, Pennsylvania, USA.; 4 Department of Genetics and Center for Molecular Studies in Digestive and Liver Diseases, Perelman School of Medicine, University of Pennsylvania, Philadelphia, Pennsylvania, USA.; 5 Division of Allergy Immunology, Children’s Hospital of Philadelphia, Philadelphia, Pennsylvania, USA.; 6 Institute for Research in Genetics and Biomedicine, CNR, Cagliari, Italy.; 7 Department of Translational and Precision Medicine, University Sapienza, Rome, Italy.; 8 Institute for Regenerative Medicine, University of Pennsylvania, Philadelphia, Pennsylvania, USA.

**Keywords:** Gastroenterology, Inflammation, Cytokines, Genetic variation

## Abstract

Cytokines mediating epithelial and immune cell interactions modulate mucosal healing—a process that goes awry with chronic inflammation as in inflammatory bowel disease. TNFSF13 is a cytokine important for B cell maturation and function, but roles for epithelial TNFSF13 and putative contribution to inflammatory bowel disease are poorly understood. We evaluated functional consequences of a novel monoallelic *TNFSF13* variant using biopsies, tissue-derived colonoids and induced pluripotent stem cell (iPSC)-derived colon organoids. *TNFSF13* variant colonoids exhibited a >50% reduction in secreted TNFSF13, increased epithelial proliferation, and reduced apoptosis, which was confirmed in iPSC-derived colon organoids. Single cell RNA-sequencing and flow cytometry suggested FAS as the predominant colonic epithelial receptor for TNFSF13, which was confirmed by co-immunoprecipitation and binding assays. Imaging mass cytometry revealed an increase in epithelial-associated B cells in *TNFSF13* variant colon tissue sections. Finally, *TNFSF13* variant colonoids co-cultured with memory B cells demonstrated a reduction in immunoglobulin-producing plasma cells compared to control colonoid cocultures. Our findings support a role for epithelial TNFSF13 as a regulator of colonic epithelial growth and epithelial crosstalk with B cells.

## Abstract 2

Epithelial TNFSF13 regulates colonic epithelial growth and epithelial- B cell interactions.

## Graphical abstract

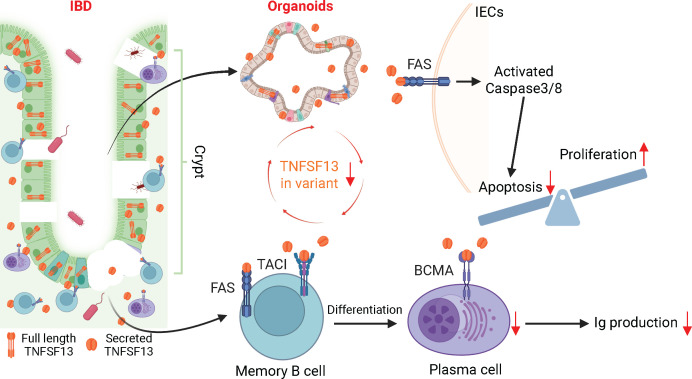

## Introduction

Inflammatory bowel disease (IBD) is attributed to a combination of factors including environment, diet, microbiota, and genetics (1). Very early onset–IBD (VEO-IBD) is a classification of pediatric IBD diagnosed in children who present with symptoms before age 6 (2, 3). Patients with VEO-IBD may exhibit more severe clinical symptoms, higher failure rates to conventional therapies, and strong family history as well as different genetic contributions to disease onset compared with older children or adults with IBD (4). The discovery and characterization of new gene variants in patients with VEO-IBD have improved our understanding not only of IBD pathogenesis, but also of fundamental intestinal biology. Reported genetic variants in patients with VEO-IBD are broadly characterized as immune, epithelial, or combined epithelial and immune in nature (5–7). The current mainstay of treatment for IBD, immunosuppressive therapy, is directed towards immune-mediated pathways, leaving an untapped opportunity for epithelial-targeted therapies (reviewed in (7)). The development of human organoid technology from affected patients’ epithelial stem and progenitor cells provides a translational model to study the physiology of intestinal epithelial cells in IBD (8). In this study, we used tissue-derived colonoids and induced pluripotent stem cell (iPSC)-derived colon organoids to investigate the mechanisms and functional roles of epithelial Tumor Necrosis Factor Ligand Superfamily Member 13 (TNFSF13/APRIL), a cytokine typically attributed to B cell maturation and function.

TNFSF13 is secreted from myeloid cells and is best characterized for its effects on immune cells (9–11). Upon binding to its receptors TACI or BCMA, TNFSF13 promotes B cell activation, proliferation, maturation, plasma cell survival, and subsequent immunoglobin production, leading to activation of anti-inflammatory pathways (12–14). A recent study described a patient with common variable immunodeficiency harboring a homozygous frameshift mutation in *TNFSF13*, which resulted in the absence of plasmablasts and increased marginal zone B cells with a normal number of B cells in blood (15). Moreover, TNFSF13 deficiency in dendritic cells impairs differentiation from memory B cells to plasma cells in vitro (15, 16). Studies in mice suggest that TNFSF13 may have roles in intestinal epithelial cell-immune cell crosstalk. Epithelial cell-secreted TNFSF13 can promote immunoglobulin A2 (IgA2) class switching triggered by bacterial sensing via Toll-like receptors (9). A different mouse study found that overexpression of epithelial TNFSF13 resulted in enhanced anti-inflammatory B cell differentiation in vitro (17). Anti-inflammatory roles of TNFSF13 have also been reported in other tissues (18–20); however, the functional roles of TNFSF13 in human intestinal epithelial cells, and putative contribution to mucosal damage or healing, are not known. The goal of the present study was to evaluate the functional consequences of a novel *TNFSF13* gene variant using organoid models and tissue analyses to understand new, fundamental epithelial biology that may elucidate previously unknown mechanisms of disease pathogenesis.

## Results

### A novel TNFSF13 variant reduces TNFSF13 expression and alters epithelial proliferation.

The current study emerged from a patient with severe colonic infantile onset IBD diagnosed at age 4 months (Supplemental Figure 1A and Supplemental Table 1; supplemental material available online with this article; https://doi.org/10.1172/JCI186032DS1), with clinical history described in Methods. Whole exome sequencing (WES) was performed on the patient and his parents and identified a *de novo* heterozygous frameshift mutation (an inserted T in exon 3) in *TNFSF13* gene (NM_003808: c.372_373insT, pAla125_Thr126fs) in the patient (Supplemental Figure 1B). Repeat immune work up was performed and while his initial immune evaluation was unrevealing, due to refractory disease, repeat studies demonstrated increased transitional B cells. While other, predominantly homozygous TNFSF13 variants have been reported (https://mastermind.genomenon.com /detail?mutatio*n* = NC_000017.11:g.7559652A%3EG), our variant was not found in 1000 Genomes, ESP, ExAC or gnomAD sequence databases and no predictions were available from PolyPhen or SIFT.

**Figure 1:**
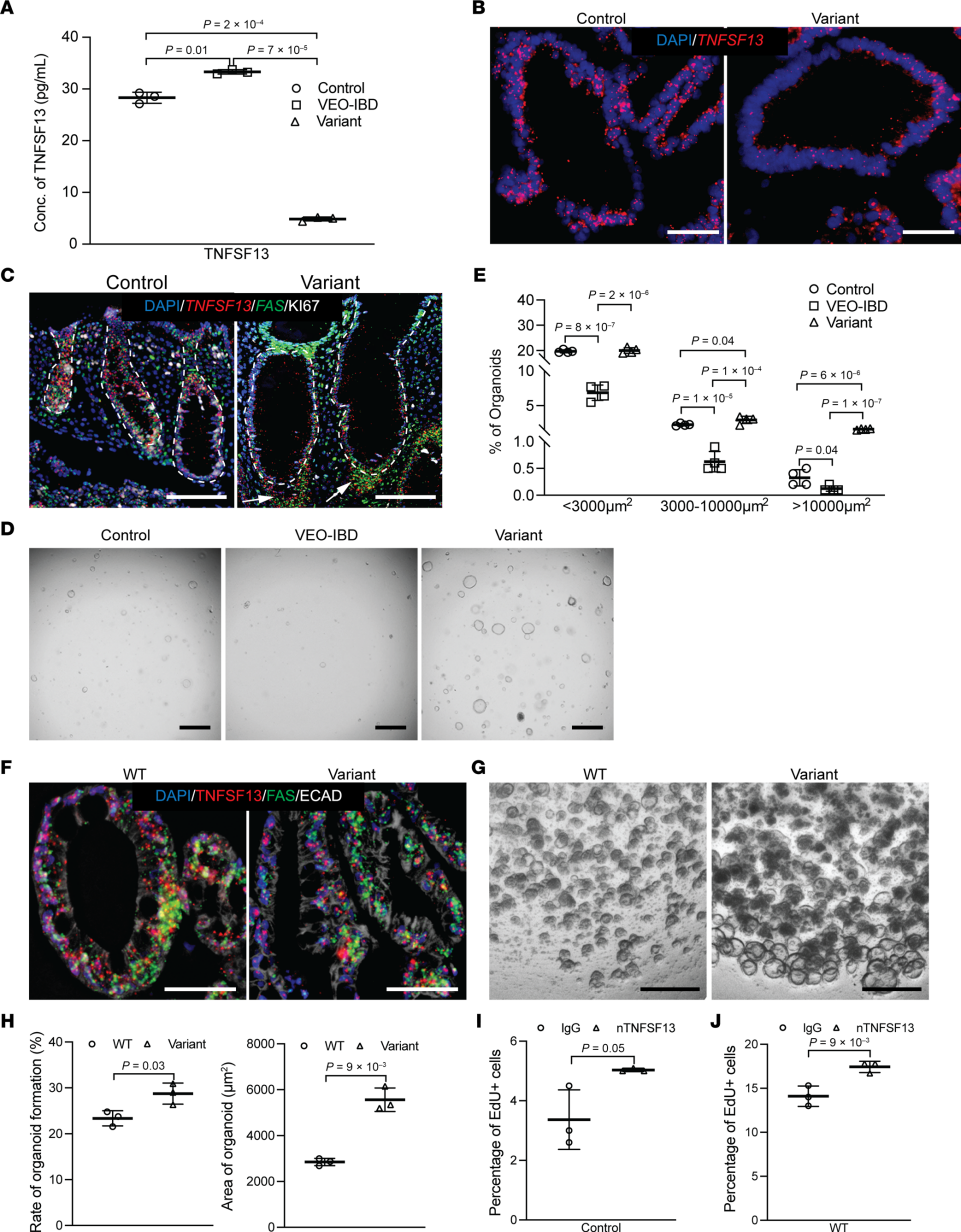
TNFSF13 variant colonoids/organoids exhibit enhanced colonoid formation efficiency and proliferation. (**A**) ELISA for secreted TNFSF13 in colonoid conditioned media (*n* = 3 patient lines for control and VEO-IBD; *n* = 3 passages for variant). (**B**) Representative TNFSF13 RNAscope in colonoids from control and variant participants (scale bar: 50 μm; *n* = 3 patient lines for control; *n* = 3 passages for variant). (**C**) Costaining of TNFSF13 and FAS RNAscope probes with Ki67 antibody in colon biopsies. Arrows indicate cells accumulated outside epithelial crypts (*n* = 3 patients for control; *n* = 3 tissue blocks for variant). (**D**) Representative images of colonoid formation assays at day 6 after seeding (scale bar: 300 μm). (**E**) Quantification of newly formed colonoids by size at day 6. Each passage included 2 technical replicates (*n* = 4 patient lines for control and VEO-IBD; *n* = 4 passages for variant). (**F**) TNFSF13 and FAS RNAscope with E-cadherin immunostaining in WT and variant iPSC-derived colon organoids at day 7 (scale bar: 50 μm; *n* = 3 passages). (**G**) Representative colonoid formation in WT and variant iPSC-derived organoids at day 9 (scale bar: 400 μm; *n* = 3 passages, each with ≥ 2 technical replicates). (**H**) Quantification of colonoid formation rate and area at day 9. Colonoid size calculated by maximum vertical projection area. (**I** and **J**) Percentage of EdU^+^ cells following IgG or TNFSF13 neutralizing antibody (nTNFSF13) treatment in (**I**) control tissue–derived colonoids (*n* = 3 patient lines) or (**J**) WT iPSC-organoids at day 7 (*n* = 3 passages). 2-way ANOVA with multiple comparisons was used for (**A** and **E**); 2-tailed Student’s *t* test for (**H**–**J**). *P* values shown on graphs unless *P* > 0.05.

Sanger sequencing confirmed the presence of the *TNFSF13* variant strand in peripheral blood mononuclear cells (PBMCs) and colonoids from the patient (Supplemental Figure 1B). qPCR with 3 different probes around variant *TNFSF13* indicated a significant decrease in *TNFSF13* mRNA compared with healthy controls and patients with VEO-IBD without an identified monogenic defect, defined herein as *TNFSF13* wild type VEO-IBD (denoted as VEO-IBD below) (Supplemental Figure 1C). This frameshift mutation caused a premature stop codon, leading to a predicted truncation in the protein via SWISS-MODEL (Supplemental Figure 1D). Typically, functional TNFSF13 is assembled into a homo- or hetero-trimer (21). Although it retained the intact transmembrane region and furin cleavage site, the truncated variant protein is predicted to lack most of its soluble region (Supplemental Figure 1, D–G). ELISA confirmed a significant decrease in secreted TNFSF13 in variant colonoid media compared to healthy control and VEO-IBD colonoids (Figure 1A). RNAscope for *TNFSF13* in variant colonoids and colon biopsies demonstrated decrease in epithelial *TNFSF13* transcript levels (indicated by individual red dots) compared to controls (Figure 1, B and C; technical controls in Supplemental Figure 2, A–C). Taken together, these data demonstrate a significant decrease of TNFSF13 on mRNA and protein levels in variant tissue.

Upon visual inspection, we noticed an increase in colonoid number and size in *TNFSF13* variant versus control patient colonoids (healthy subjects and *TNFSF13* wild type VEO-IBD) at day 6 post-seeding (Figure 1, D and E, and Supplemental Figure 2D). We directly assessed organoid formation efficiency via single cell plating and measured colonoid size as a proxy for proliferative capacity. Colonoids were significantly more numerous and larger in *TNFSF13* variant versus controls (Figure 1, D and E). To confirm whether the observed colonoid formation efficiency and size phenotypes were driven by variant *TNFSF13* and not a consequence of the tissue state at the time of biopsy, we generated a human induced pluripotent stem cell (iPSC) line with the same variant and compared it to a WT *TNFSF13* isogenic control line. After directed differentiation into colon organoids (22), RNAscope, qPCR, and ELISA demonstrated the variant line had decreased *TNFSF13* compared with WT (Figure 1F and Supplemental Figure 2, E–G). Furthermore, single cell–seeded organoid formation assays showed higher organoid formation efficiency and size in *TNFSF13* variant versus WT organoids (Figure 1, G and H, Supplemental Figure 2H). Since our data demonstrated that epithelial TNFSF13 may have antiproliferative roles in nonvariant cells, we used a TNFSF13 neutralizing antibody on control colonoids and WT iPSC-derived colon organoids to evaluate proliferation directly using EdU incorporation. We confirmed the ability of the antibody to neutralize TNFSF13 using dose curves in mouse splenic B cell proliferation assays as published previously (23) (Supplemental Figure 3, A–C). We observed an increase in EdU^+^ proliferative cells in both control patient colonoids and WT colon organoids treated with TNFSF13 neutralizing antibody compared to IgG control (Figure 1, I and J). Conversely, the percentage of EdU^+^ cells in variant colonoids was rescued after treatment with recombinant TNFSF13 (Supplemental Figure 3, D and E). Taken together, TNFSF13 neutralization and rescue data are consistent with our observation that decreased TNFSF13 expression promotes increased organoid size as a result of increased proliferation.

### TNFSF13 binds FAS receptor in colonic epithelial cells.

**Figure 2:**
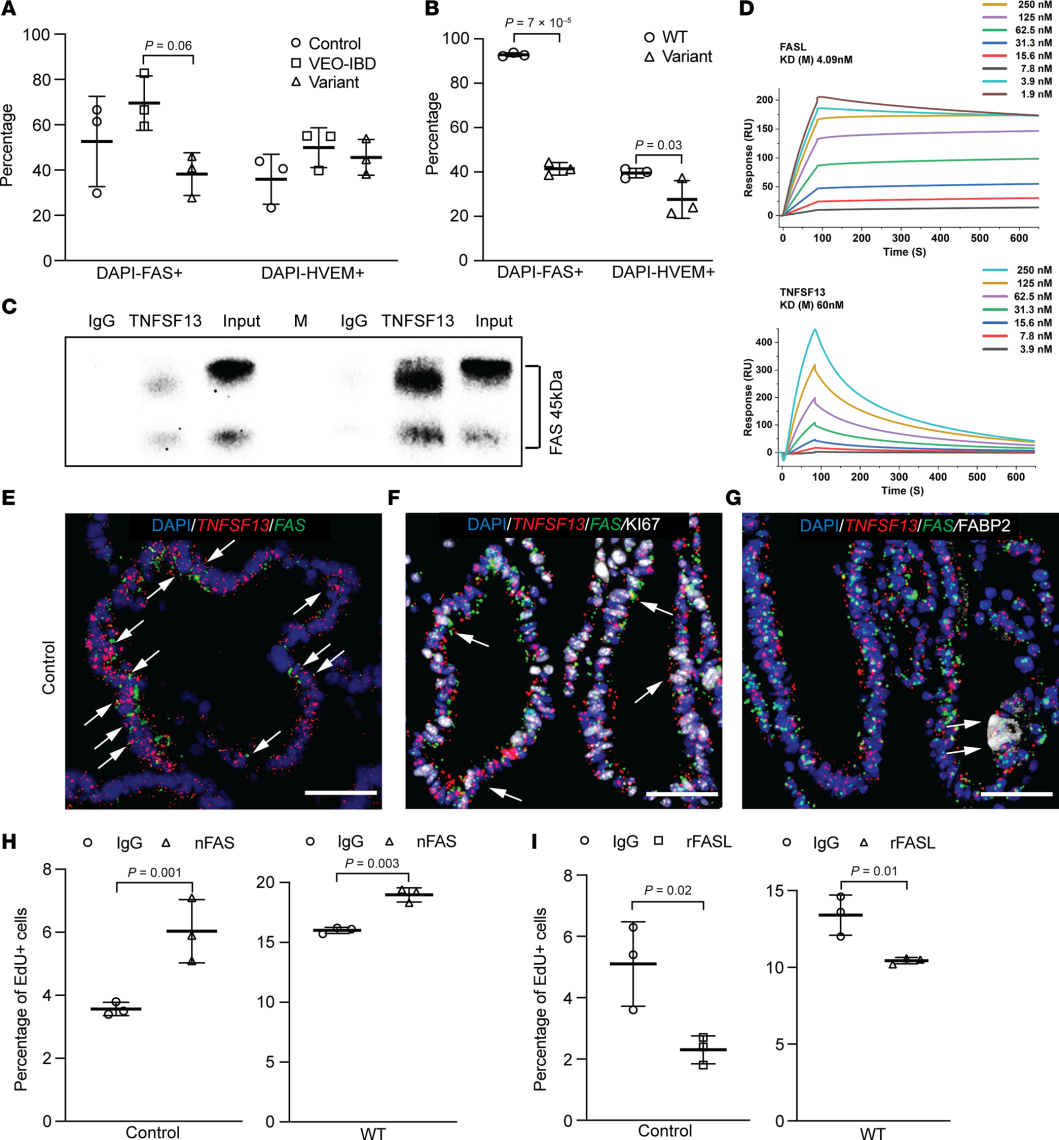
FAS is a receptor for TNFSF13 in colonic epithelial cells. Percentage of FAS^+^ and HVEM^+^ cells by flow cytometry at day 7 after seeding in (**A**) colonoids (*n* = 3 patient lines for control and VEO-IBD; *n* = 3 passages for variant) or (**B**) iPSC-organoids (*n* = 3 passages per line). (**C**) Representative Western blot for FAS from coimmunoprecipitation supernatant in control and VEO-IBD colonoids at day 7. TNFSF13 antibody used for capture. Protein ladder lane (M) not shown in overlay (*n* ≥ 4 patient lines for control and VEO-IBD). (**D**) SPR binding analyses of FASL-FAS (upper) and TNFSF13-FAS (lower) interactions at various concentrations (*n* = 3 independent experiments). (**E**) Costaining of TNFSF13 and FAS RNAscope probes in control colonoids at day 7. Arrows indicate coexpression (scale bar: 100 μm; *n* = 3 experiments). (**F**) Co staining of TNFSF13 and FAS RNAscope probes with Ki67 antibody in control colonoids at day 7. Arrows indicate triple-positive cells (scale bar: 100 μm; *n* = 3 experiments). (**G**) Costaining of TNFSF13 and FAS RNAscope probes with FABP2 antibody in control colonoids at day 7. Arrows indicate triple-positive cells (scale bar: 100 μm; *n* = 3 experiments). (**H** and **I**) Percentage of EdU+ cells in control colonoids (left) or WT iPSC-organoids (right) at day 7 following treatment with (**H**) IgG or FAS neutralizing antibody (nFAS) or (**I**) IgG or recombinant human FAS ligand (rFASL). Two-way ANOVA with multiple comparisons for (**A** and **B**); 2-tailed Student’s *t* test for (**H** and **I**). *P* values shown unless *P* > 0.05.

TNFSF13 can bind to multiple surface receptors in different cell types (12–14). We investigated expression of these receptors in tissue-derived colonoids and iPSC-derived colon organoids. Flow cytometry (FACS) analysis revealed that TACI and BCMA, which are abundant in B cells and plasma cells, are not detected by FACS in colonic epithelial cells (Supplemental Figure 4, A and B). Instead, FACS implicated the lesser known TNFSF13 receptors FAS and HVEM (24) were detected in colonoids and iPSC colon organoids (Figure 2, A and B). TNFSF13 and FAS interactions have been predicted in synovium-DRG neuron interactome (25). Since FAS has been associated previously with proliferation and apoptosis (26), we next tested whether TNFSF13 can interact with the FAS receptor in colonic epithelial cells via co-immunoprecipitation (co-IP). We observed that FAS is only detected when the capture antibody for TNFSF13 is present, in contrast to IgG controls (Figure 2C). To verify TNFSF13 binding FAS protein, we conducted in silico analyses using the HDOCK online server (http://hdock.phys.hust.edu.cn/) (27) and found multiple specific interactions between FAS and TNFSF13: Ser172 and Lys148 of FAS formed hydrogen bonds with Gln51 and Tyr212 of TNFSF13, with bond distances of 2.2 Å and 2.4 Å, respectively (Supplemental Figure 5A lower). In addition, His142 of FAS formed two hydrogen bonds with Arg144 of TNFSF13, with distances of 2.5 Å and 2.8 Å. A salt bridge was also observed between Asp144 of FAS and Arg144 of TNFSF13, with 3.9 Å (Supplemental Figure 5A, lower panel). Furthermore, multiple hydrophobic interactions contributed to the stabilization of the FAS–TNFSF13 complex (Supplemental Figure 5A lower). We also evaluated the well-defined ligand of FAS (FASL) as a positive control, which can bind to Trp14, Glu87, Glu32, Thr312, Arg192 of FAS through Thr40, Tyr244, Arg44, Gly41, Thr34 of FASL, respectively (Supplemental Figure 5A, upper panel). Docking and confidence scores calculations suggest that FAS is predicted to interact with FASL more strongly than TNFSF13 (docking score = −322.73, confidence = 96% versus docking score = −284.45, confidence = 93%); however, typical protein-protein interactions in the Protein Data Bank have a docking score of around -200 or better (more negative) with a confidence score above 70% indicating the two molecules would be very likely to bind. As such, both TNFSF13 and FASL are predicted to bind to FAS. Finally, we performed Surface Plasmon Resonance (SPR) to quantify direct binding interactions between FAS and TNSF13 and FASL. Both TNFSF13 and FASL exhibited concentration-dependent binding to immobilized FAS with clear saturation kinetics (Figure 2D). Fitting sensorgrams with to a 1:1 kinetic binding model indicated equilibrium dissociation constants of 60 nM for FAS-TNFSF13 and 4.09 nM for FAS–FASL, indication higher affinity of FAS for FASL than for TNFSF13 (Figure 2D). To assess whether TNFSF13 and FASL compete for FAS binding, we performed competitive SPR assays. Neither condition resulted in detectable changes in binding response for the competing ligand, suggesting that TNFSF13 and FASL do not compete for FAS binding under these conditions (Supplemental Figure 5B).

We performed additional studies to interrogate TNFSF13-FAS interactions. RNAscope data indicate expression of TNFSF13 and FAS in human colonoids, providing spatial evidence of their co-expression in the same and neighboring cells, including in Ki67^+^ proliferating cells and FABP2^+^ enterocytes (Figure 2, E–G, white arrowheads). Immunofluorescence staining (IF) data further show TNFSF13 and FAS co-localized in FAS-expressing HEK293T cells (Supplemental Figure 5C). To functionally validate FAS in colonic epithelial cells, we treated control colonoids and WT iPSC colon organoids with a FAS neutralizing antibody and observed a significant increase in EdU^+^ cells by flow cytometry (Figure 2H). Conversely, recombinant human FAS ligand decreased EdU^+^ cells (28, 29) (Figure 2I). Variant colonoids and variant iPSC colon organoids exhibited similar responses to FAS modulation, suggesting that the proliferative phenotype observed in variant organoids at baseline likely results from loss of TNFSF13-mediated FAS activation (Supplemental Figure 5, D and E). FAS is a TNF superfamily receptor commonly described as a pro-apoptotic factor; however, some studies demonstrate additional roles such as NFκB activation, among other roles (30). FAS neutralization phenocopied the proliferative effect of TNFSF13 neutralization in our cultures systems, supporting a model in which TNFSF13-FAS signaling negatively regulates colonic epithelial proliferation in vitro.

### Transcriptome analysis reveals altered apoptosis pathways in TNFSF13 variant epithelium.

**Figure 3:**
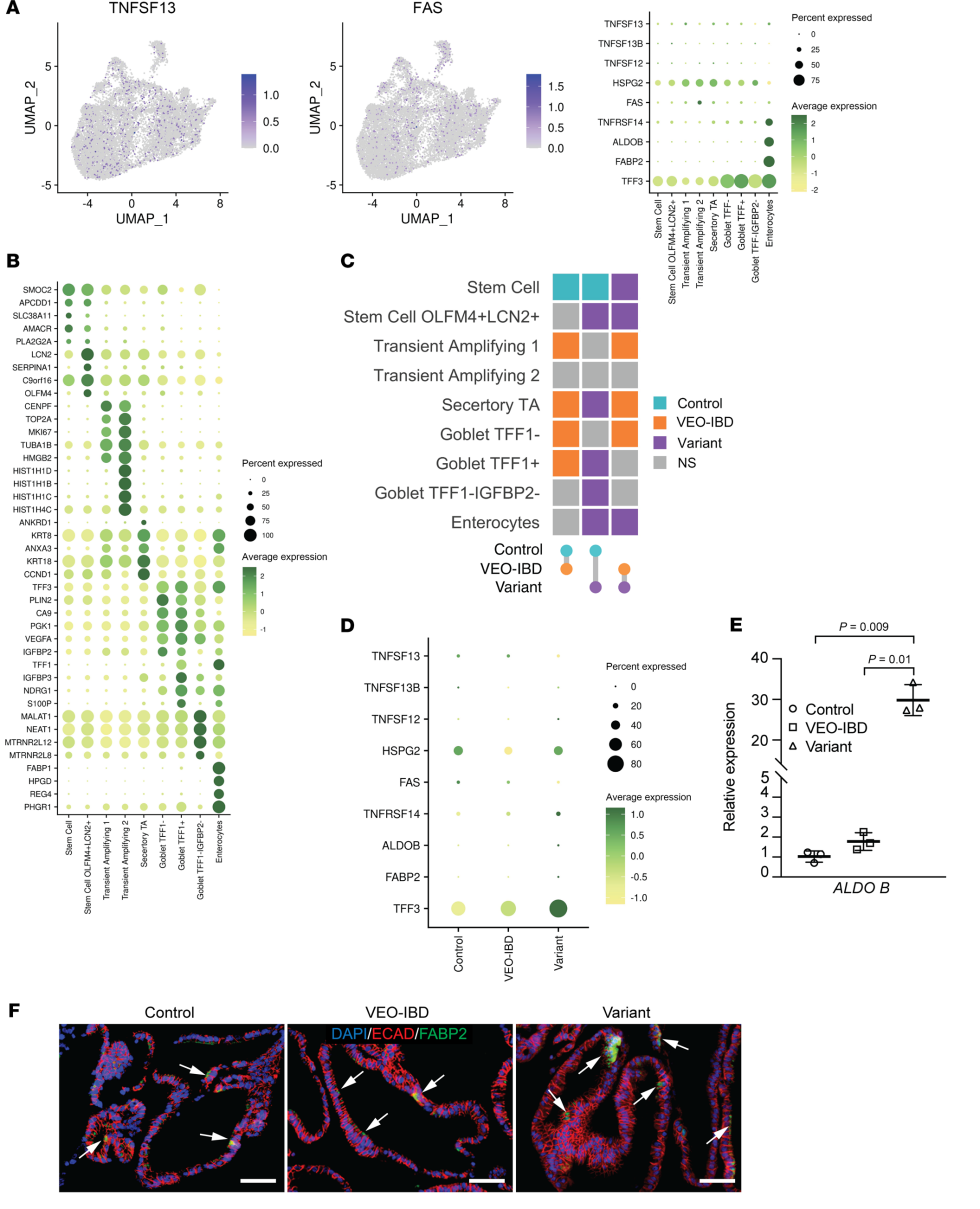
Transcriptomic profiling in human colonoids. (**A**) UMAP showing the expression pattern of *TNFSF13* and *FAS* in scRNA-seq data from human tissue–derived colonoids. (*n* = 2 lines of colonoids from 2 different patients for control and VEO-IBD, *n* = 2 independent passages of colonoids for variant.) Dot plot indicating the relative expression pattern of selected genes of *TNFSF13* family and related receptors and enterocyte markers among annotated clusters for human colonoids scRNA-seq data. (**B**) Dot plot with relative expression of top 5 changed genes for each annotated cluster for scRNA-seq datasets in human colonoids. (**C**) Color scale indicates group with higher percentage of cells within a given cluster in each comparison. The color indicates the condition with higher percentage of a cluster in each pairwise comparison. (**D**) Dot plot with relative expression of selected genes of TNFSF13 family and related receptors and enterocyte markers among control, VEO-IBD and variants in human colonoids. (**E**) qPCR for ALDOB in colonoids on d7 after seeding. *n* = 3 lines of colonoids from 3 different patients for control and VEO-IBD, *n* = 3 passages of colonoids for variant. One-way ANOVA (with multiple comparisons) was used for statistical analysis. (**F**) Representative IF images for FABP2 and E-cadherin in human colonoids. White arrows denote FABP2^+^ cells. Scale bar: 50 μm. *P* values are shown on bar graphs unless *P* > 0.05.

We next evaluated transcriptional differences between control, *TNFSF13* wild type VEO-IBD and *TNFSF13* variant colonoids via single cell RNA sequencing (scRNA-Seq). Based on previously annotated marker genes (31), we identified and assigned colonic epithelial cells to 9 clusters: 2 stem cell clusters, 3 transit-amplifying (TA) cell clusters, 3 goblet cell clusters and 1 enterocyte cluster which were visualized by uniform manifold approximation and project (UMAP) (Figure 3, A and B; cell counts in Supplemental Table 3). UMAP and dot plot of combined samples demonstrated broad expression of TNFSF13 and FAS in human colonoids, especially in TA cells and colonocytes (Figure 3A; dark purple dots on UMAP). Overall, we observed moderate but likely inconsequential shifts in other cell type proportions when evaluating UMAPs of control, VEO-IBD, and variant colonoids separately (Figure 3B and Supplemental Figure 6A).

Evaluation of cell type proportions demonstrated that stem cells with expression of inflammatory marker LCN2 are significantly increased in TNFSF13 variant colonoids (denoted as “Stem Cell OLFM4^+^LCN2^+^”), as are goblet cells (denoted as “Goblet TFF1^+^” and “Goblet TFF1^–^IGFBP2^–^”) and colonocytes (Figure 3C). Likewise, gene expression analysis demonstrated increased expression of goblet cell marker *TFF3* and enterocyte markers *ALDOB* (32) and *FABP2* (33) in *TNFSF13* variant colonoids, albeit in a small percentage of cells (Figure 3D). We confirmed increased expression of *ALDOB* by qPCR (Figure 3E) and immunofluorescence (IF) staining for FABP2 (Figure 3F; white arrows) in *TNFSF13* variant colonoids compared with control and VEO-IBD colonoids. We also performed scRNA-seq on fresh biopsies from the same *TNFSF13* variant participant and an additional healthy control participant (Supplemental Table 3) and annotated clusters using published cell markers from human biopsies (34). Analysis of biopsy scRNA-seq data confirmed expression of *TNFSF13* and *FAS* in epithelial cells and a similar lack of robust differences in cell type proportions between *TNFSF13* variant and controls, as seen in respective colonoid lines (Supplemental Figure 6, B–F). We conclude that phenotypic differences between *TNFSF13* variants and controls are not due to significant changes in lineage allocation between groups.

**Figure 4:**
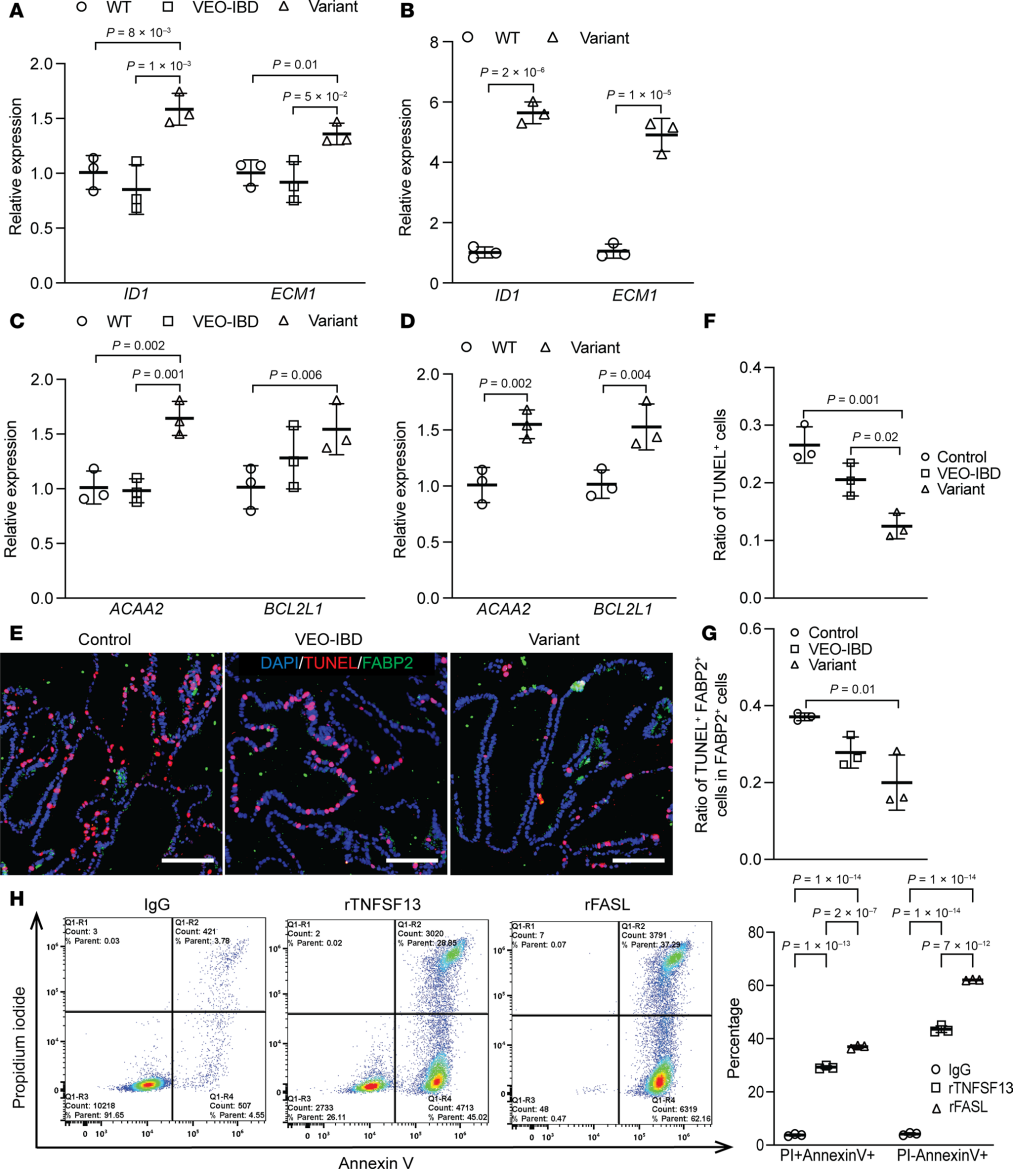
TNFSF13 augments the balance of apoptosis and proliferation through FAS-apoptosis pathway. (**A**) qPCR for *ID* and *ECM1* in colonoids and (**B**) iPSC-derived colon organoids. (**C**) qPCR for *ACAA2* and *BCL2L1* in tissue-derived colonoids from control, VEO-IBD, variant, and (**D**) iPSC organoids from WT and variants. *n* = 3 lines of colonoids from 3 different patients for Control and VEO-IBD, *n* = 3 passages of colonoids for variants. (**E**) Representative immunostaining images for TUNEL and FABP2 in colonoids. Scale bar: 100 μm. (**F**) Quantification for ratio of TUNEL^+^ cells per colonoid and (**G**) TUNEL^+^FABP2^+^ cells in FABP2^+^ cells. One-way ANOVA (with multiple comparisons) was used for statistical analysis with 3 independent replicates. (**H**) Left: Representative flow cytometry plots for apoptotic cells in IgG, rTNFSF13 and rFASL treated Jurkat T cells. Right: Percentage of PI^+^AnnexinV^+^ (late apoptotic) and PI^–^AnnexinV^+^ (early apoptotic) populations across treatments. Three independent experimental replicates are shown. *P*-value shown in the bar graphs unless *P* > 0.05. Two-way ANOVA (with multiple comparisons) was used for statistical analysis in **A**–**D** and **H** and 1-way ANOVA (with multiple comparisons) for **F** and **G**.

To explore putative mechanisms of the TNFSF13-FAS axis in colonic epithelial cells, we performed Gene Ontology (GO) enrichment analysis of the combined scRNA-seq data (Supplemental Table 4). Consistent with phenotypic data, we observed an enrichment of pathways involved in epithelial cell proliferation and apoptosis in *TNFSF13* variant versus VEO-IBD or healthy controls (Supplemental Figure 7A, red arrowheads). Colonoid qPCR data confirmed the relative increased expression of proliferation-associated genes, *ID1* and *ECM1* (35, 36) (Figure 4, A and B), and mitochondrial anti-apoptotic genes, *ACAA2* (37) and *BCL2L1* (38) (Figure 4, C and D) in *TNFSF13* variant versus VEO-IBD or healthy controls. Immunostaining for apoptosis (TUNEL) demonstrated significantly fewer TUNEL^+^ cells and TUNEL^+^ FABP2^+^ cells in *TNFSF13* variant versus control colonoids (Figure 4, E–G), which could explain the increase in enterocytes observed in *TNFSF13* variant colonoids in Figure 3. Flow cytometry for AnnexinV and PI confirmed a significant increase in apoptotic cells in rTNFSF13- or rFASL-treated Jurkat T cells compared with IgG control (Figure 4H), with FASL inducing a stronger apoptotic response than TNFSF13. Consistently, immunoblot indicated increased BCL-XL (antiapoptotic protein encoded by *BCL2L1*) (38) and cleaved capase3 in both *TNFSF13* variant colonoids and iPSC colon organoids compared with respective controls (Supplemental Figure 7, B and C). We observed more cleaved caspase 3 and caspase 8 in rTNFSF13- or rFASL-treated Jurkat T cells compared with IgG control, whereas BCL-XL levels did not show the same changes observed in organoid models (Supplemental Figure 7D). The discrepancy between primary organoid models and Jurkat T cells could be due to differences in cellular context and experimental system, or through other mechanisms to be evaluated in future studies. Taken together, transcriptomics, histological analyses, and immunoblot data support the conclusion that TNFSF13 insufficiency both enhances proliferation and limits apoptosis in colonic epithelial cells, particularly colonocytes.

### Epithelial TNFSF13 regulates tissue-associated memory B cell differentiation.

TNFSF13 is best characterized for its roles in regulating proliferation and differentiation in B cells and plasma cells. We therefore evaluated circulating and tissue immune populations in *TNFSF13* variant and control participants. We first examined peripheral blood immune changes using flow cytometry of PBMCs (Supplemental Figure 8A). We observed an increase in CD19^+^ B cells in *TNFSF13* variant blood compared with healthy controls, but not as much as compared with *TNFSF13* WT VEO-IBD (Supplemental Figure 8, A and B). CD19^+^CD27^+^CD38^+^ plasmablasts, switched plasmablasts, and IgM memory B cells were relatively lower in *TNFSF13* variants compared with VEO-IBD (Supplemental Figure 7B). There were no significant differences in IgM^+^ plasmablasts, nor in memory, class-switched memory (CSM), naive or transitional B cells, or other immune cells (T cell, nature killer cell, and monocyte) in *TNFSF13* variants compared with healthy control and VEO-IBD PBMCs (Supplemental Figure 8, B and C). Interestingly, although He et al. showed that epithelial-derived TNFSF13 promotes IgA class switching in mice (9), our data did not show obvious change in class-switched memory B cells, which may be due to the difference between human and mouse. Consistent with the previous report of increased IgM^+^IgD^low^CD27^+^ B cells (MZB) (15), the percentage of the marginal zone–like (MZ-like) B cells in the variant patient is also relatively increased compared with VEO-IBD. Taken together, there were moderate but nonsignificant differences in peripheral B cells in *TNFSF13* variant versus control participants.

**Figure 5:**
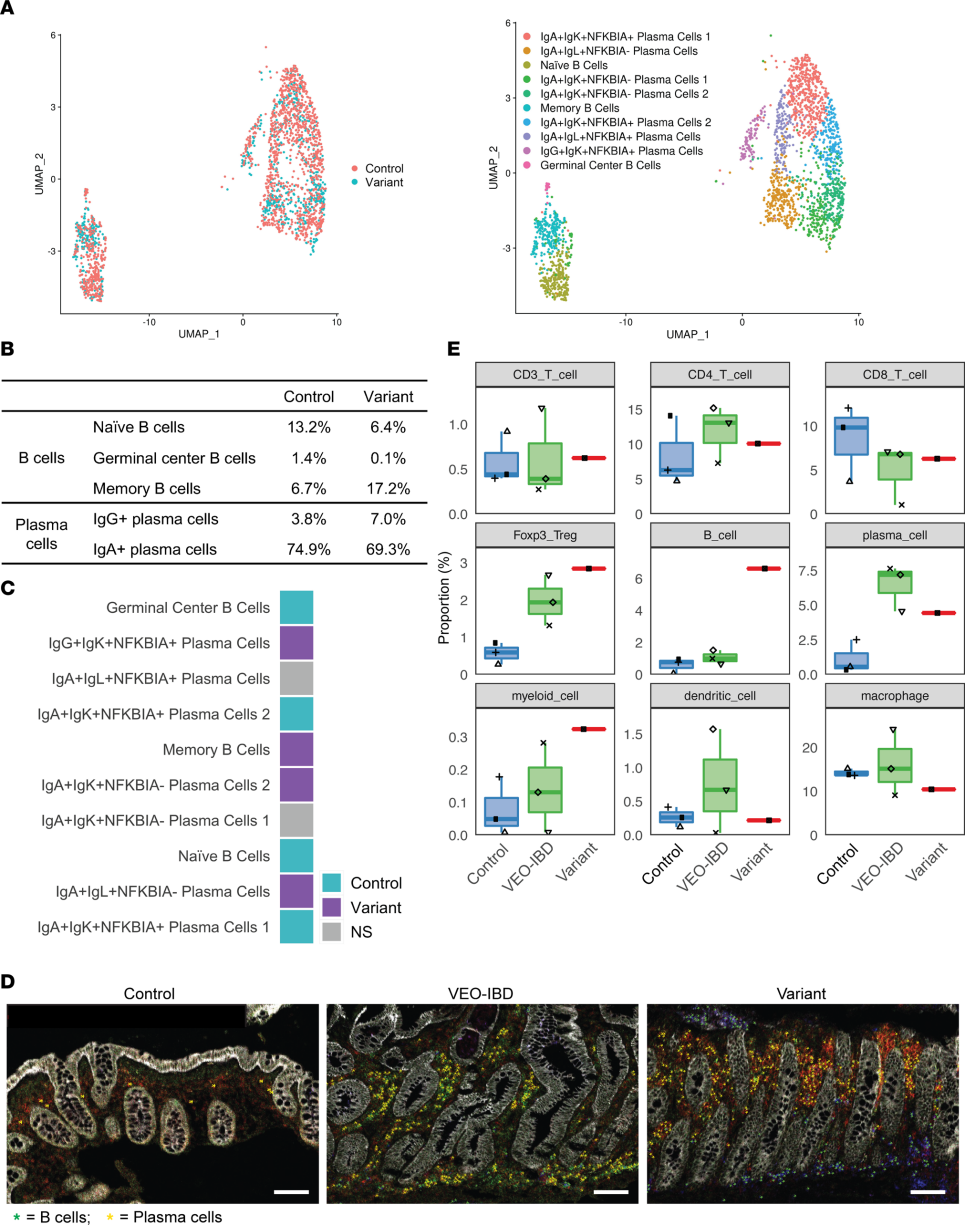
Increased abundance of memory B cells and depletion of IgA^+^ plasma cells observed in *TNFSF13* variant colon. (**A**) UMAP visualizations of scRNA-seq data for B cell and plasma cell clusters among lamina propria cells from control and variant colon biopsies. *n* = 1 patient for control and variant. Left: Overlay of control and variant samples; Right: Annotated cell clusters of control and variant samples. (**B**) Table indicates abundance (%) of B cell and PC subsets in control and variant samples from scRNA-seq data from variant and control colon biopsies. (**C**) Comparison of cell type abundance between samples from scRNA-seq data from variant and control colon biopsies. Color scale indicates which group has a higher percentage of cells within a given cluster. (**D**) Representative IMC overlay images of epithelial (white), B cell (green), and plasma cell (yellow) markers in colon from control, VEO-IBD and variant patient. Scale bar: 100 μm. Marker for B cell: CD20^+^; Markers for plasma cell: CD20^–^CD27^+^CD38^+^. *n* = 3 different patients for Control and VEO-IBD, *n* = 3 slides from different blocks for variants. (**E**) Boxplot showing the rate of immune cell composition quantified by calculating the proportion of specific markers in all cells at the same region (both lamina propria and epithelial cell populations). *n* = 3 different patients for control and VEO-IBD, *n* = 3 slides from different blocks for variant.

The immune cells within the intestinal mucosa play an essential role in the establishment and regulation of intestinal inflammation and injury in IBD (39). We therefore explored immune changes in colonic tissue of *TNFSF13* variant and control participants. We first evaluated 6,014 variant and 4,755 control cells in the scRNA-seq data of lamina propria layer from the same biopsies as described above, which were subclustered into 6 subsets (Supplemental Figure 9, A–C). We further subclustered B cells (germinal center B cells [GC B cells], memory B cells, and naive B cells) and plasma cells (7 clusters based on Ig types IgA, IgK, IgL, IgG, and NFKBIA signature) populations (40) (Figure 5A). Cell type abundance analysis indicated fewer germinal center B cells and naive B cells, but more memory B cells in *TNFSF13* variants compared with control biopsies (Figure 5, B and C). These findings are consistent with prior reports of increased memory B cell recruitment and differentiation to plasma cells under inflammatory conditions (41). For plasma cells, although the population of IgA^+^IgK^+^NFκBIA^–^ and IgA^+^IgL^+^NFκBIA^–^ cells are relatively increased, total IgA^+^ PCs (approximately 69.3%) were relatively decreased in *TNFSF13* variant compared with control (approximately 74.9%) (Figure 5C). In contrast, we noticed IgG^+^ plasma cells, which have been reported to contribute to IBD (41), were relatively increased.

Imaging mass cytometry (IMC) is a multiplexed imaging platform that utilizes antibodies conjugated to heavy metal isotopes, permitting quantification of different cell types within local tissue niches (42). IMC identified 9 major immune cell populations within colon sections from 7 patients (3 controls, 3 TNFSF13 WT VEO-IBD, and 1 *TNFSF13* variant with 2 different biopsies)): CD3^+^ T cells, CD4^+^ T cells (T helper cells), CD8^+^ T cells (cytotoxic T cells), FOXP3^+^ regulatory T cells (Tregs), B cells, PCs, myeloid cells, dendritic cells, and macrophages (Figure 5D, Supplemental Figure 10, A and B, and Supplemental Table 5). Cell annotation markers are listed in Supplemental Materials and Methods. Because IMC retains the X and Y coordinates of each cell in each image, we were able to assess immune cell composition with spatial resolution. We found increased CD20^+^ total B cells near epithelial crypts in *TNFSF13* variants compared with control and VEO-IBD tissue (Figure 5, D and E, pictured in green at crypt base). Plasma cell numbers in *TNFSF13* variants were lower than VEO-IBD, but higher than controls (Figure 5E). Given that TNFSF13 promotes proliferation and differentiation of B and plasma cells (9), we evaluated cell abundance of proliferative B cells and plasma cells combined with Ki67 staining for proliferation as a putative explanation for aggregation of B cells in *TNFSF13* variant tissue. The percentage of Ki67^+^ total B cells in *TNFSF13* variant sections was lower than control and *TNFSF13* WT VEO-IBD sections, suggesting that accumulated B cells in *TNFSF13* variant tissue is likely due to enhanced recruitment rather than increased B cell proliferation (Supplemental Figure 10C). Similarly, the percentage of Ki67^+^ plasma cells in *TNFSF13* variant tissue was lower than in VEO-IBD tissue (Supplemental Figure 10C). Taken together, scRNA-seq and IMC data suggest that decreased TNFSF13 in variant tissue might reduce differentiation of memory B cells to IgA-producing plasma cells and could contribute to accumulation of B cells near epithelial crypts. These newly described phenotypes in *TNFSF13* variant tissue may separately contribute to mucosal damage via (a) reduced beneficial epithelial–plasma cell interactions (43) and (b) aberrant B cell accumulation in the epithelial compartment, which studies in mice suggest may hinder stromal contributions to mucosal healing (44).

### Coculture studies confirm epithelial TNFSF13-mediated B cell modulation.

**Figure 6:**
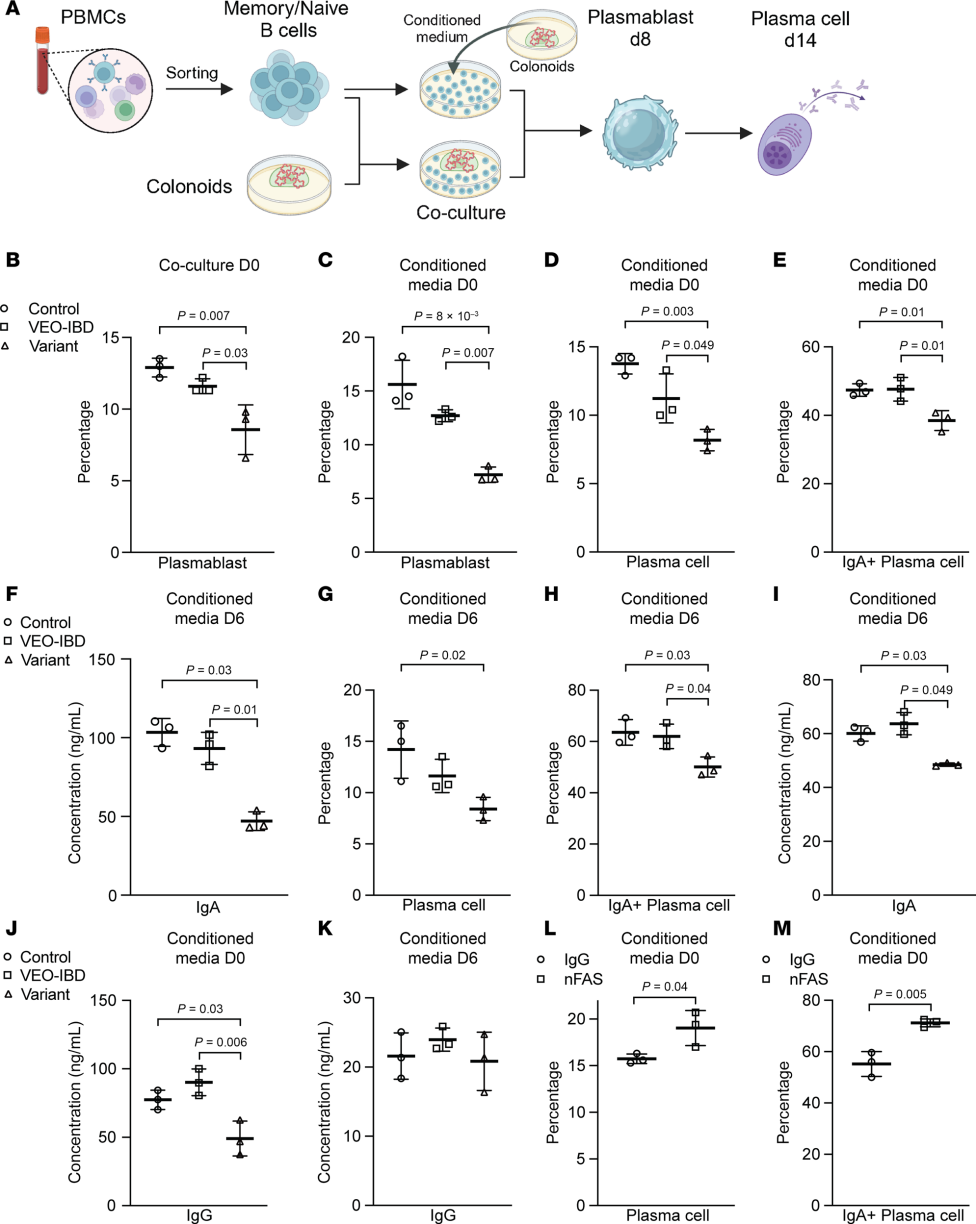
Epithelial-secreted TNFSF13 modulates differentiation of memory B cells to plasmablasts and plasma cells. (**A**) Schematic of human memory B cell and colonoid coculture experimental model. (**B**) Percentage of plasmablasts differentiated from sorted human memory B cells at day 8 via co-culture with equal numbers of control, VEO-IBD, or variant colonoids in IntestiCult:B cell media (1:1). (**C**) Percentage of plasmablasts at day 8 cultured in B cell media:conditioned media (1:1). (**D** and **E**) Percentage of (**D**) plasma cells and (**E**) IgA^+^ plasma cells differentiated at day 14 in B cell media:conditioned media (1:1). (**F**) IgA ELISA from differentiated B cell culture media at day 14. (**G** and **H**) Percentage of (**G**) plasma cells and (**H**) IgA+ plasma cells at day 14 when conditioned media mixture was added starting day 7 (cells cultured in B cell media alone days 0–6. (**I**) IgA ELISA from B cell culture media at day 14 with conditioned media added starting day 6 (*n* = 3 independent replicates). (**J**) ELISA for IgG in media from differentiated human memory B cells at day 14 and (**K**) at day 14 with delayed (day 6) conditioned media addition. (**L** and **M**) Percentage of (**L**) plasma cells and (**M**) IgA^+^ plasma cells at day 14 from IgG or FAS neutralizing antibody–treated (nFAS-treated) B cells cultured in control colonoid-conditioned media:B cell media (1:1). Three independent control colonoid lines used. Unless otherwise noted, *n* = 3 patient lines for control and VEO-IBD; *n* = 3 passages for variant colonoids and iPSC-organoids; *n* = 11 independent B cell donors. One-way ANOVA with multiple comparisons or 2-tailed Student’s *t* test used for statistical analysis. *P* values shown unless *P* > 0.05.

Through engagement of BCMA and TACI, TNFSF13 has been reported to promote plasma cell maintenance, class-switch recombination, and immunoglobulin secretion, thereby supporting humoral immune responses and B cell homeostasis across multiple tissue contexts (12–14). Prior studies reported that TNFSF13 defects in dendritic cells or monocyte-derived dendritic cells differentiated from iPSCs impaired memory B cell proliferation and differentiation to plasmablasts and then to plasma cells (15, 16). To investigate the function of epithelial-secreted TNFSF13 on memory B cell differentiation, we developed a series of methods to either directly coculture human colonoids with memory B cells or use human colonoid–conditioned media to treat memory B cells or naive B cells activated with R848 (Figure 6A). Coculture of sorted human memory B cells (flow sorted Zombie UV^–^CD19^+^CD20^+^CD27^+^ or isolation with EasySep Human Memory B Cell Isolation Kit) with equal numbers of human control, VEO-IBD, and variant colonoids at day 8 after seeding indicated that the percentage of differentiated plasmablasts (ZombieUV^–^FSC^hi^CD20^+^CD27^+^CD38^+^CD138^lo^) significantly decreased in variant conditions (Figure 6B and Supplemental Figure 11, A and B, and Supplemental Figure 12A). Since mixing colonoid and B cell media at a ratio of 1:1 only permitted short-term coculture, we collected 2-day conditioned media from equal numbers of human control, VEO-IBD, and variant colonoids and mixed it with human B cell media (ratio of 1:1) for treatment of cultured B cells. Consistent with coculture studies, we found the percentage of plasmablasts differentiated from sorted/isolated human memory B cells significantly decreased in variants at day 8 after seeding with colonoid conditioned media and B cell media mixture (Figure 6C, Supplemental Figure 11, A and B, and Supplemental Figure 12, A–C). We also found a reduction in the percentage of plasma cells (ZombieUV^–^FSC^hi^CD20^−^CD27^hi^CD38^hi^CD138^+^) that differentiated from plasmablasts at day 14 after seeding in variant media–treated B cell cultures (Figure 6D). Given that plasma cells are an important source of immunoglobin, including IgA, IgG, and IgM, we examined the IgA^+^ population in total plasma cells, which revealed a decrease in the percentage of IgA^+^ plasma cells in variant media conditions (Figure 6E). ELISA for IgA in media at day 14 after seeding confirmed that secreted IgA was decreased in B cells with variant conditioned media compared with control and VEO-IBD (Figure 6F). We next evaluated the role of TNFSF13 in differentiation of plasmablasts to plasma cells by maintaining memory B cells in B cell media for 6 days to get an equal percentage of plasmablasts and then changing to conditioned media mixture from control, VEO-IBD, or variant colonoids at day 6 after seeding. Flow cytometry and ELISA data at day 14 after seeding indicate the percentage of both plasma cells and IgA^+^ plasma cells decreased in variant media conditions, which is consistent with differentiation directly from memory B cells (Figure 6, G–I, and Supplemental Figure 12D). Similarly, we investigated the expression level of IgG (presumably from IgG^+^ memory B cells) and IgM and found both were relatively decreased in variant media–treated memory B cells (Figure 6, J and K, and Supplemental Figure 12, E and F).

Since naive B cells also can differentiate into short-lived plasma cells (45, 46), we examined differentiation of naive B cells treated with a combination of variant colonoid–conditioned media and B cell media supplemented with R848. Flow cytometry data indicated that the percentage of plasmablasts and plasma cells decreased in variant media–treated naive B cells (Supplemental Figure 12, G and H). Our data and those from other groups (47) indicate that FAS is broadly expressed in memory B cells and lymphocytes (Supplemental Figure 12 I, left). To test whether FAS modulation can augment memory B cell differentiation, we treated memory B cells with the FAS neutralizing antibody described above (nFAS) or IgG control in control conditioned media. The percentage of plasmablasts did not change in nFAS-treated memory B cells at day 8 after seeding (Supplemental Figure 12 I, right); however, both plasma cells and IgA^+^ plasma cells were significantly increased compared with IgG control (Figure 6, L and M). To determine whether this FAS-mediated effect requires TNFSF13, we repeated these experiments in variant conditioned media. FAS neutralization in variant media did not significantly change plasmablast, plasma cell, or IgA^+^ plasma cell frequencies (Supplemental Figure 13, A and B). Conversely, treatment with recombinant FASL in variant media significantly decreased both plasmablasts and plasma cells compared with IgG control (Supplemental Figure 13, C and D). These data support a potential role for FAS in memory B cell differentiation and suggest that this regulatory function is, in part, dependent on TNFSF13. While our findings were initially unexpected, a recent study leveraged FAS-deficient B cells from patients with autoimmune lymphoproliferative syndrome (ALPS) to uncover nonapoptotic roles for FAS signaling in B cell fate decisions. In that study, authors describe a mechanism in which nonapoptotic FAS signaling modulates mTOR activation in FAS-competent B cells, which they demonstrate is defective in B cells from patients with ALPS with deficient FAS signaling that may underlie low memory B cells in patients with ALPS (48). Given the importance of memory B cells and immunoglobulin-producing plasma cells for immune homeostasis in the gastrointestinal tract (43), our data suggest that changes in the epithelial-secreted TNFSF13-FAS axis may be an unappreciated mechanism contributing to disease (6).

## Discussion

Local cues from intestinal epithelial cells can shape the functional specificity of immune responses, and understanding new mechanisms of epithelial-immune interactions is critical for designing new, epithelial-targeted treatments for IBD. We used reductionist organoid culture systems to dissect novel epithelial cell roles for TNFSF13 in epithelial and immune cells of the human colon. We demonstrated that TNFSF13 secreted by colonic epithelial cells can act upon epithelial cells themselves to increase proliferation and decrease apoptosis in vitro. We further identified FAS as the putative TNFSF13 receptor expressed in epithelial cells that can modulate apoptosis signaling either directly through caspase-8–mediated proteolysis of effector caspases (i.e., caspases-3 and caspase-7) or through mitochondria-released apoptosome to propagate the caspase activation cascade (49). These findings are particularly intriguing, as dysregulated cell death can be an important feature in patients with IBD.

When broadening our analyses to tissue biopsies, we found dysregulation of local immune responses in the colon, particularly affecting B cell and plasma cell populations in *TNFSF13* variant tissue, as reported previously (9, 10, 15). Prior studies demonstrated that TNFSF13 is secreted by intestinal epithelial cells upon Toll-like receptor–mediated bacterial sensing, leading to IgA2 class switching. These findings support a dual-hit model whereby (a) depleted epithelial TNFSF13-FAS axis signaling promotes an imbalance in proliferation and apoptosis leading to aberrant wound healing and (b) depleted epithelial-derived TNFSF13 leads to insufficient antibody production in response to antigen exposure. While we do not provide data in direct support of the latter, our scRNA-seq and imaging mass cytometry data demonstrate that TNFSF13 deficiency is associated with decreased plasma cells and increased B cell accumulation in the colonic lamina propria. These findings suggest that, while the patient’s total quantitative immunoglobulins were normal, under tissue stress, mucosal changes in TNFSF13 could lead to impaired tissue immunoglobulin levels. In addition, we provide data that FAS neutralization augments B cell fate in vitro, supporting putative roles for the TNFSF13-FAS axis to affect both epithelial and B cell compartments. Our findings are timely, since a recent report in mice demonstrated that B cell depletion is beneficial for mucosal healing in experimental colitis (44). This same study identified a robust expansion of an IFN-induced population of B cells marked by CD274 and Ly6a during the mucosal healing phase following experimental colitis in mice. We evaluated our scRNA-seq data for CD274 and additional reported marker genes from this study but did not find an analogous cell cluster in our human biopsy data (not shown).

Patients with antibody defects can develop IBD, and the contribution of B cells to IBD pathogenesis has been a long-standing area of interest in the field (reviewed in ref. 50). Recently, aberrant mucosal B cell diversity and maturation were reported via scRNA-seq analysis in patients with ulcerative colitis (41). As such, we decided to pursue epithelial B cell interactions via 3D coculture. While 2D coculture systems, including epithelial cells and B cells, have been reported previously, coculture in 3D-culture systems in human primary cells are only recently emerging (51–53). Coculture of human primary B cells with 3D epithelial colonoids has not been reported. In this study, we developed a human primary memory B cell and colonoid coculture system to explore secreted epithelial TNFSF13 and memory B cell maturation. This system not only provides an accessible method to study signaling between epithelial and immune compartments in vitro but also provides a powerful tool to explore dual tissue compartment changes associated with genetic variants in patients. While TNFSF13 has been described previously as a regulator of B cell maturation, our model permitted this paradigm to be tested directly in primary human cells. Optimization of this coculture model will allow for future studies to further evaluate epithelial B cell interactions, including reciprocal effects of B cells on epithelial cells or with the addition of other immune or stromal cell types, as has been reported in mice (44).

Our study has limitations. It is tempting to speculate that phenotypes observed in TNFSF13 variant colonoids are due to a generalized disease state rather than a variant-specific state. Our generation and evaluation of *TNFSF13* variant- and isogenic control iPSC-derived colon organoids provided orthogonal data in support of epithelial TNFSF13 functions that have not been described previously. Furthermore, retention of in vivo inflammatory components during colonoid establishment could hinder epithelial cell growth and be a confounding variable. To mitigate this concern, we evaluated all colonoid samples between passages 6 to 15. In addition, we noticed the percentage of FAS^+^ cells between patient tissues and organoid models are not consistent — FAS^+^ cells are higher in variant tissue compared with control tissue, whereas FAS^+^ cells are comparable in variant versus control colonoids. Given that organoids are derived from proliferative crypt-derived epithelial stem cells and may not fully recapitulate the cellular heterogeneity present in patient tissues, direct comparison of the overall percentage of FAS^+^ cells in patient biopsies versus organoids should be interpreted with caution. A separate limitation of our study is that TNFSF13 deletion has been reported previously in both human and in mice, where intestinal or IBD-like symptoms are not prominent (15, 54). Our study therefore suggests that TNFSF13 insufficiency may contribute to, rather than cause, IBD. Alternatively, our specific variant could have unknown functions leading to the colonic manifestations observed in the patient. As such, biochemical studies of our variant and analyses in conditional mouse models will be needed to disentangle dose- and tissue-dependent effects of TNFSF13 and direct links to the complex clinical presentation observed in the present patient.

While data presented herein are based upon a single patient with variant *TNFSF13,* there is precedent for leveraging single patient findings to better understand pathology underlying groups of human diseases (55). As such, our findings may have broader implications. A recent study demonstrated that recombinant TNFSF13 can restore plasmablast differentiation in vitro in a dendritic cell B cell co-culture experiment using cells from a patient with common variable immunodeficiency harboring a different *TNFSF13* variant (15). It is therefore possible that restoration of TNFSF13 could be a tractable therapy for patients with other *TNFSF13* variants. It is also possible that, given the mechanism of disease, patients with variants in *TNFSF13* may not have a sustained response to conventional IBD therapies. In addition, our finding that *TNFSF13* variant colonic epithelial cells exhibit increased proliferation and decreased apoptosis may indicate the need for earlier and more frequent cancer surveillance, as this cellular phenotype likely increases risk of colorectal cancer even more than already exists for patients with IBD. In summary, our findings demonstrate novel roles for TNFSF13 to modulate the balance of proliferation and apoptosis in colonic epithelial cells. An imbalance of proliferation and apoptosis, together with aberrant mucosal B cell dynamics observed in the present study, underscores the importance of identifying mechanisms converging upon epithelial and immune compartments that could serve as future therapeutic targets.

## Methods

### Sex as a biological variable.

Both male and female patients were included in the study.

### Participant enrollment and demographics.

Biopsy specimens, human peripheral blood mononuclear cells (PBMCs), and histological samples were obtained from deidentified patients. The patient with the TNSF13 variant presented with colonic IBD at 4 months of age with diarrhea and poor growth. Due to medically refractory disease, the patient underwent diverting ileostomy at 21 months of age and over time developed progressive stricturing disease requiring subsequent hemicolectomy with sparing of the right colon. The patient developed sacroilitis postoperatively and ultimately achieved remission of intestinal and joint disease with infliximab and rapamycin. Immunologic workup was performed at the patient’s initial presentation that was unrevealing, including lymphocyte subsets, immunoglobulins, DHR, and FOXP3 analysis. As part of the research protocol, biosamples were obtained from patients with VEO-IBD and healthy controls. Patients with VEO-IBD were diagnosed at less than or equal to 6 years old of age using standard methods of endoscopic, radiologic, laboratory and clinical evaluation. Indications for colonoscopy in patients with VEO-IBD included diagnosis, change in disease status, and surveillance of disease. All patients with VEO-IBD underwent immunologic and genetic evaluation. Control VEO-IBD participants denoted as *TNFSF13* WT do not have a known or candidate monogenic disorder. Genetic studies were carried out through whole exome sequencing (WES) and included trio analyses. Healthy control samples were selected from subjects undergoing colonoscopy for the following reasons: abdominal pain, poor growth, rectal bleeding, or diarrhea, and had normal endoscopic and histologic findings. Individuals with a previous diagnosis of other intestinal or systemic inflammatory disease, including chronic allergic or inflammatory diseases, were excluded. Detailed patient information and the purpose for each sample used in this study can be found in Supplemental Table 1.

### Whole exome sequencing, variant calling, and annotation.

Whole exome sequencing was performed on the variant patient and his parents (Supplemental Table 1). Library preparation and exome capture were performed using the Agilent SureSelect v4 capture kit with DNA samples isolated from PBMCs. Sequence reads were aligned to the reference human genome (GRCh37) using the Burrows–Wheeler alignment (BWA) algorithm (v.0.7.15) (56) and variants were called using GATK’s best practices. Variants were functionally annotated with information from multiple databases including dbSNP (57), dbNSFP (58), 1000 Genomes Project (59), and the Genome Aggregation Database (gnomAD v2.1.1) (60) using SnpEff (http://snpeff.sourceforge.net) then filtered to retain only moderate- and high-effect, rare (minor allele frequency < 1%) variants. Trio analysis for the patient included identifying variants that are de novo, compound heterozygous, homozygous, and X-linked, and results were limited to variants within genes known to be associated with VEO-IBD or immunodeficiency.

### Generation of colonoids from patient biopsies.

Mucosal biopsies were obtained from endoscopically affected and unaffected areas of the terminal ileum and/or left/right colon during colonoscopy procedures conducted for disease surveillance or diagnosis (Supplemental Table 1). The collected samples were promptly transported in cold DMEM (Corning, New York, USA) on ice to the laboratory for subsequent crypt isolation. To generate patient-derived colonoids, biopsies were rinsed once in cold DPBS (Corning, New York, USA) and then in chelation buffer. Subsequently, they were incubated in a cold, fresh 0.5M EDTA chelation buffer comprising 2% sorbitol (Fisher Scientific, Massachusetts, USA), 1% sucrose (Sigma-Aldrich, Massachusetts, USA), 1% BSA (Fisher Scientific, Massachusetts, USA), and 1x Gentamicin (Thermo Fisher Scientific, Massachusetts, USA) in DPBS for a duration of 30 minutes. Following incubation, the biopsy samples were gently scraped off using forceps to release crypts. The isolated crypts were then resuspended in 30–50 μL of Matrigel (Corning, New York, USA) after filtrating with 70 μm nylon strainer, with the exact volume adjusted based on the number of crypts and plated to achieve an optimal density. Successful isolation was defined by the presence of at least 50 crypt units per biopsy region, and plating volumes of Matrigel were meticulously adjusted to ensure uniform crypt density and minimize potential density-related growth bias. Colonoids were fed every other day in human IntestiCult Organoid Growth Media (Stem Cell Technologies, British Columbia, Canada) and collected or split roughly 7 days after plating, depending on the specific experiment. When colonoids reached an appropriate density to avoid overgrowth, mechanical passaging was initiated as outlined below: colonoids were suspended in 3 mL cold advanced DMEM/F12 (Thermo Fisher Scientific, Massachusetts, USA) and then pipetted up and down 5 times in 15 mL conical tube using a p1000 μL pipette tip fitted with p200 μL pipette tip. Subsequently, the collected colonoids were spun down, resuspended, and replated in the Matrigel at an appropriate volume to maintain uniform density. 10 μM Y-27632 (LC Laboratories, Massachusetts, USA) was added for the first 2 days. Media was changed every other day. All colonoid lines in this study were utilized between Passage 6 and Passage 15 for consistency and reliability.

### Generation of human induced pluripotent stem cell–derived organoids.

Human induced pluripotent stem cell (iPSC) lines carrying the same heterozygous frameshift mutation in TNFSF13 as human variant patient (NM_003808: c.372_373insT, pAla125_Thr126fs), and WT iPSC line were generated and validated by the Children’s Hospital of Philadelphia Human Pluripotent Stem Cell Core using the parental line CHOPWT14 described previously (61). Feeder-independent culture, expansion, and differentiation of both the WT and variant iPSC lines were performed at the University of Pennsylvania (UPenn) iPSC Core. To prepare cells for differentiation, iPSCs were seeded on 0.12 mg/ml Geltrex LDEV-free reduced growth factor basement membrane matrix (ThermoFisher Scientific, Massachusetts, USA, 1:100 diluted in cold DMEM/F12 from the same vendor) precoated plates (1 hour at RT) and maintained with mTeSR1 complete medium at 37°C with 5% O_2_ and CO_2_ incubator. Human iPSC-derived organoids were differentiated and maintained as previously described protocol with the following adjustments (22). Approximately 5 million live single cells (per well of a 6-well plate) were seeded on Corning Matrigel (diluted 1:30 in cold DMEM/F12) coated plate after digesting with Gentle Cell Dissociation Reagent for 10 min at 37°C (with the addition of 10 μM Y-27632 for the first 10 days). The STEMdiff Definitive Endoderm Kit (Stem Cell Technologies, British Columbia, Canada) was used to differentiate monolayer cultures to definitive endoderm (DE) once cells reached around 90%–100% confluency on day 1 or day 2 after plating. Following a 4-day DE differentiation with sequential administration of supplements MR and CJ according to the manufacturer’s instructions, the cells was transitioned to hindgut endoderm (HE) differentiation by treating with HE media (containing 3 μM CHIR99021- Cayman Chemical Company, 1x GlutaMAX-Thermo Fisher Scientific, 1x Pen/Strep-Thermo Fisher Scientific, 0.5 μg/mL FGF4-PeproTech, and 1x B27 supplement-Thermo Fisher Scientific in RPMI 1640-Corning) for another 4 days (fresh HE media was changed daily). At the end of HE differentiation, cells were primed for colonic differentiation over a 12-day period in colonic medium comprised of advanced DMEM/F12 with 3 μM CHIR99021, 1x GlutaMAX, 1x Pen/Strep, 0.3 μM LDN193189-Cayman Chemical Company, 1× B27 supplement and 0.1 μg/mL EGF-R&D Systems. Colonic media was refreshed daily, and detached spheroids were collected and reseeded in Corning Matrigel simultaneously with colonic media. Differentiated colonic cells were disassociated into single cells by Accutase for 10 min at 37°C and then seeded in Matrigel with colonic media (with the addition of 10 μM Y-27632 for the first 2 days). iPSC-derived organoids were obtained from both detached spheroids and seeded colonic cells. Organoids were fed every other day and split every 7 days. To remove other types of cells, single cell suspension from organoids, digested by 0.05% trypsin at 37°C for 10 min (10% FBS-Peak Serum as final ratio to deactivated trypsin), was subjected to fluorescence-activated cell sorting (FACS) with MoFlo Astrios sorter (Beckman Coulter, Pennsylvania, USA) or FACSAria Fusion Sorter (BD Biosciences, New Jersey, USA) in the CHOP Flow Core after incubating with PE anti-CD326 (EpCAM) Monoclonal Antibody (G8.8) (ThermoFisher Scientific, Massachusetts, USA) and DAPI for 30 min on ice in dark. Sorted DAPI^–^EpCAM^+^ intestinal cells were seeded and expanded in Matrigel (approximately 50,000 live cells per 30 μL Matrigel; 10 μM Y-27632 was added for the first 10 days and then split as normal) to generate organoids for subsequent analysis and experiments.

### Mouse B cell isolation/maintenance, treatment, and resazurin assay.

To isolate mouse B cells, normal mouse spleen was harvested from adult WT mice and processed into single cell suspension by mincing it through a 70 μm cell strainer placed in a 6-well plate containing 5 mL of 1x DPBS, using the flat end of a plunger from a 3-cc syringe. Mouse experiments were approved under IACUC protocol 001278 at the Children’s Hospital of Philadelphia. The pellet was collected by centrifugation at 300*g* at 4°C and incubated in 5 mL of 1x RBC lysis buffer (Thermo Fisher Scientific, Massachusetts, USA) per spleen for 5 min at room temperature with occasional shaking to remove blood cells. The reaction was stopped by adding 20 mL of 1x DPBS. Subsequently, the cells were collected and incubated with FITC anti-mouse CD19 [6D5] (Biolegend, California, USA) diluted at 1:50 in FACS buffer for 30 min on ice in the dark. DAPI at a final concentration of 0.1 μg/mL was add for an additional 10 min. After washing with 3 mL FACS buffer and resuspending in 1mL FACS buffer, the cells were sorted with a MoFlo Astrios sorter (Beckman Coulter, Pennsylvania, USA) or FACSAria Fusion Sorter (BD Biosciences, New Jersey, USA) in CHOP Flow Core. Specifically, 100,000 sorted DAPI-CD19+ B cells per well were plated in a 96-well plate with 100 μL RPMI 1640 with L-Glu media (Corning, 10%FBS+1xAnti-Anti+10mM HEPES-- Thermo Fisher Scientific + 50uM β-Mercaptoethanol-- Thermo Fisher Scientific) at 37°C/5%CO_2_. To stimulate B cells, 10μg/mL of F(ab’)2-Goat anti-Mouse IgM (mu) Secondary Antibody (Cat# 16-5092-85, Thermo Scientific, Massachusetts, USA) was added to the media upon plating. To evaluate the function of TNFSF13 in B cells, recombinant Human TNFSF13 (HEK293-expressed) protein (Cat #5860-AP-010, R&D Systems, Minnesota, USA) at a varying concentration gradient (0, 0.0005, 0.001, 0.005, 0.01, 0.05, 0.1, 0.2, 0.5, 1, 1.5, 2 μg/mL) was added at 4h post plating. Meanwhile, to assess the efficiency of neutralizing antibody, nTNFSF13 (Cat #MAB5860, R&D Systems) with a concentration gradient (0, 0.005, 0.01, 0.05, 0.1, 0.5, 1, 5, 10, 20, 30, 50 μg/mL) was added along with 500 ng/mL recombinant Human TNFSF13 protein for each well at 4h post plating. For measuring proliferation in B cells, Resazurin (Cat# AR002, R&D Systems) was added to all wells at a volume equal to 10% of the cell culture volume (10 μL) and incubated for another 4 hours in incubator. Fluorescence was measured using a TECAN Infinite 200Pro microplate reader (TECAN, Zürich, Switzerland) with a wavelength of 544 nm excitation and 590 nm emission. Negative control wells containing only media were set up in parallel to account for background effects. The process and analysis of data was conducted using an online tool (https://www.aatbio.com/tools/four-parameter-logistic-4pl-curve-regression-online-calculator) and the equation form was created as shown in Supplemental Figure 2, B and C.

### Human memory B cell isolation/differentiation, treatment, and analysis by flow cytometry.

Human PBMCs from 7 independent donors were purchased from UPenn Human immunology Core (supported by NIH P30 AI045008 and P30 CA016520). To isolate memory B cells, human PBMC cell pellets were collected by centrifuging at 300*g* for 5min at 4°C, and then incubated in FACS buffer (2% FBS in DPBS) with indicated antibodies in the dark on ice for 30 min: anti-CD19 Mouse Monoclonal Antibody (PE) [clone: SJ25C1] (BioLegend), anti-CD27 Mouse Monoclonal Antibody (FITC) [clone: M-T271] (BioLegend), BD Pharmingen APC-H7 Mouse Anti-Human CD20 (BD Biosciences), Anti-CD20 Mouse Monoclonal Antibody (PE/Cy7) [clone: 2H7] (BioLegend), anti-human IgA Antibody (APC) (Miltenyi Biotec, Cologne, Germany). DAPI (Sigma-Aldrich, Massachusetts, USA) was added at a final concentration of 0.1μg/mL for an additional 10 min. After washing with 3 mL FACS buffer, cells were resuspended and subjected to flow cytometry using a MoFlo Astrios (Beckman Coulter) or FACSAria Fusion Sorter (BD Biosciences) in the CHOP Flow Core. Following sorting, 67,000–150,000 sorted DAPI^–^CD19^+^CD20^+^CD27^+^ memory B cells were seeded equally in a 96-well plate with 150μL B cell medium, consisting of RPMI 1640 with L-Glu media +10%FBS+1xAnti-Anti+10mM HEPES + 1μg/mL R848 (Cat# tlrl-r848, Invivogen, California, USA) + 50uM β-Mercaptoethanol at 37°C/5%CO2, IntestiCult/B cell media mixture, or conditioned media mixture, respectively. Media was changed every other day. Human memory B cells and naive B cells were also isolated by using EasySep Human Memory B Cell Isolation Kit (STEMCELL Technologies) according to manufacturer’s instructions.

For coculture of memory B cell with human colonoids, 3,000 clumps of control, VEO-IBD, variant colonoids were seeded in 45 μL Matrigel with 500 μL human IntestiCult Organoid Growth Media for the first 2 days (considered colonoid seeding day as d-2). After 2 days of growth, an equal number of sorted DAPI^–^CD19^+^CD20^+^CD27^+^ memory B cells (or isolated with EasySep Human Memory B Cell Isolation Kit) were seeded in colonoid wells with 500 μL mixture media (IntestiCult media: B cell media = 1:1) (considered B cell seeding day as d0). Mixture media was changed every other day. At d8, differentiated memory B cells were collected without disturbing the Matrigel dome and subjected to FACS to examine the percentage of plasmablasts. Differentiated memory B cells were collected by centrifugation at 300*g* for 5 min at 4°C, and then incubated in FACS buffer (2% FBS in DPBS) with various antibodies for 30 min in the dark on ice: anti-CD27 Mouse Monoclonal Antibody (FITC) [clone: M-T271] (BioLegend), BD Pharmingen APC Mouse Anti-Human CD38 (BD Biosciences), anti-CD138 Mouse Monoclonal Antibody (PE) [clone: MI15] (BioLegend). DAPI (Sigma-Aldrich, Massachusetts, USA) was added at a final concentration of 0.1 μg/mL for an additional 10 min. After washing with 3 mL FACS buffer, cells were resuspended and analyzed with an LSR Fortessa analyzer (BD Biosciences).

For culturing memory B cells with conditioned media mixture, conditioned media was initially obtained by seeding 3,000 clumps of control, VEO-IBD, and variant colonoids in 45 μL Matrigel with 500 μL human IntestiCult Organoid Growth Media. Fresh conditioned media was then collected from the 2-day conditioned human IntestiCult Organoid Growth Media in human colonoids wells every other day (d2, d4, d6, d8, d10), respectively. Moreover, the remaining conditioned media collected at d4 after seeding was centrifuged and stored for ELISA to test the expression level of secreted TNFSF13. To create conditioned media mixture, collected fresh conditioned media was centrifuged at 300*g* for 5 min at 4°C to remove debris and then mixed with an equal volume of fresh B cell media. An equal number of sorted DAPI^–^CD19^+^CD20^+^CD27^+^ memory B cells (or memory B cells isolated with EasySep™ Human Memory B Cell Isolation Kit) were seeded in the fresh conditioned mixture media (considered d0) and were fed every other day. To examine the percentage of plasma cells and IgA^+^ plasma cells, differentiated memory B cells were collected at d14 by centrifugation at 300xg for 5min at 4°C, and then incubated in FACS buffer (2% FBS in DPBS) with various antibodies for 30 min in the dark on ice: anti-CD19 Mouse Monoclonal Antibody (PE) [clone: SJ25C1] (BioLegend), anti-human CD19 Antibody (Alexa Fluor 700) (BioLegend), anti-Human CD38 (APC) (BD), anti-CD27 Mouse Monoclonal Antibody (FITC) [clone: M-T271] (BioLegend), anti-Human CD20 (APC-Cy7) (Thermo Fisher Scientific), anti-CD138 Mouse Monoclonal Antibody (Brilliant Violet® 605) [clone: MI15] (BioLegend), anti-human IgA Antibody (APC) (Miltenyi Biotec), anti-human IgA Antibody PE (SouthernBiotech), anti-Human IgG Antibody (APC) (BD). DAPI (Sigma-Aldrich) at a final concentration of 0.1μg/mL was added for an additional 10 minutes, or Zombie UV Fixable Viability Kit (Biolegend) was used where indicated according to manufacture instructions. After washing with 3 mL FACS buffer, cells were resuspended and analyzed with an LSR Fortessa (BD Biosciences) or Cytek Aurora spectral analyzer (Cytek Biosciences). Plasmablast (FSC^hi^CD20^+^CD27^+^CD38^+^CD138^lo^) and plasma cell (FSC^hi^CD20^−^CD27^hi^CD38^hi^CD138^+^) populations were defined as described previously (62). Before FACS (d14 post-seeding), media in each well was collected and stored at -80°C after centrifugation for testing the expression level of IgA, IgG, and IgM with ELISA kits.

### Study approval.

This study was conducted with the approval of the Institutional Review Board (IRB): IRB # 14-010826. All parents of patients provided written informed consent.

### Data availability.

Transcript profiling data are deposited in U.S. National Library of Medicine Gene Expression Omnibus (GEO) with accession number GSE243445. All other data are available from the corresponding author upon request. Transcript profiling data is deposited in U.S. National Library of Medicine Gene Expression Omnibus (GEO) with accession number GSE243445.

Values for all data points in graphs are reported in the Supporting Data Values file.

Additional materials and methods are provided in the online Supplemental Materials and Methods.

## Author contributions

KEH had full access to all data in the study and takes responsibility for the integrity of the data and the accuracy of the data analysis. KEH and JRK conceptualized and supervised the study. KEH, JRK, XM, SKN, and YT designed and conducted all the experiments and manuscript drafting. JRK, KES, KHK, MD, SKN, NR, and DAP provided critical review of the manuscript for important intellectual content. Bioinformation analysis was performed by ND and XM. JRK, RS, MAC, DAP, and MD were involved in patient inclusion and sample acquisition. AK and XM performed IMC staining and data analysis. SKN, YT, YL, TAK, PAW, KM, LRP, LAS, and CHD helped with protocol optimizing, conducting experiments, and material support.

## Funding support

This work is the result of NIH funding, in whole or in part, and is subject to the NIH Public Access Policy. Through acceptance of this federal funding, the NIH has been given a right to make the work publicly available in PubMed Central.

- NIH R01 DK124369 (KEH).
- Lisa Dean Moseley Stem Cell Grant (KEH).
- Children’s Hospital of Philadelphia (CHOP) Institutional Development Funds (KEH).
- CHOP Roberts Collaborative Pilot Grant (ND).
- NIH R01AI146026 (NR.
- NIH R01 AI179680 (NR).
- The Jeffrey Modell Foundation (NR).
- NIH R01DK127044 (JRK).
- The National Natural Science Foundation of China 82403064 (YT).

## Supplementary Material

Supplemental data

Unedited blot and gel images

Supporting data values

## Version 1 04/01/2026

Electronic publication
