# Supplementary material for: TNFSF13 insufficiency disrupts human colonic epithelial cell growth and associated B cell dynamics
Source: J Clin Invest. 2026 Apr 1;136(7):e186032. doi: 10.1172/JCI186032 (PMC13038214; doi:10.1172/JCI186032)
Supplement: Supplemental data [file jci-136-186032-s267.pdf]

TNFSF13 insufficiency disrupts human colonic epithelial cell growth and associated B cell dynamics

Supplementary Materials

1. Supplemental Methods

2. Supplemental Methods References

3. Supplemental Figures and Figure legends

4. Supplemental Tables

SUPPLEMENTARY METHODS

TOPO TA clone and sanger sequencing. TOPO® TA Cloning® Kits for Sequencing (Thermo Fisher Scientific) were employed to validate the haplotype of both variant and control sequences according to the manufacturer's protocol. Briefly, mRNA isolated from human PBMCs, colonoids for variant and control, as well as iPSC-derived organoids from variant and WT lines, was reverse transcribed to generate cDNA to serve as the template for TA cloning, following the protocol in above kit. PCR product was generated using a 50 µL PCR reaction mixture comprised of DNA Template 10-100 ng, 10X PCR Buffer 5 µL, 50 mM dNTPs 0.5 µL, and water to a final volume of 49 µL, Taq Polymerase (1 unit/µL) 1 µL (Cat# M0273S, New England Biolabs, Massachusetts, USA), specific primers (~200 ng each, Genewiz, New Jersey, USA) 1 µM for each with standard cycling parameters in thermal cycler ProFlex™ 3 x 32-well PCR System (95 ºC 5min, 95 ºC 30s, 56 ºC 30s, 72 ºC 40s, 35 cycles, 72 ºC 10min, 4 ºC) (Supplementary Table 2). The resulting PCR product was assessed using agarose gel electrophoresis.

For the TOPO® Cloning reaction, a mixture containing fresh PCR product (4 µL), Salt Solution (1 µL), water (to a final volume of 5 µL), and TOPO® vector (1 µL) was prepared and incubated at room temperature for 5 min. Subsequently, 2 µL of the TOPO® Cloning reaction and 1 vial of One Shot® chemically competent E. coli were gently mixed and incubated on ice for 5 min. This mixture was then subjected to a 30-second heat-shock at 42 ºC, followed by immediate transfer to ice. Pre-warmed LB Broth (Miller) medium (Sigma-Aldrich) was added, and the mixture was horizontally shaken at 37 ºC for 1 hour. Following the incubation, 50 µL of the mixture was spread onto a pre-warmed selective plate (containing LB Agar Broth with 50 µg/mL ampicillin--

Qiagen, Hilden, Germany) according to the manufacturer's protocol and submitted to GENEWIZ for Sanger sequencing with the universal primer M13 forward and M13 reverse. The sequencing data were analyzed using the Benchling online tool (https://www.benchling.com/) to determine and confirm the haplotype of the variant and control sequences.

Histological analyses and immunostaining. Human colonoids or organoids were collected, fixed in 4% paraformaldehyde (PFA, VWR) in 1x PBS for 24 hours at 4°C, transferred to 70% ethanol and then submitted to the Molecular Pathology and Imaging Core (MPIC) of UPenn to create histological samples/unstained slides. To simultaneously detect the expression of RNAscope probes and antibodies, RNAscope® Multiplex Fluorescent v2 Assay (Cat# 323100) was employed, combined with Immunofluorescence Integrated Co-Detection kit (Advanced Cell Diagnostics, California, USA) according to the manufacturer's protocol. Briefly, after baking for 1 hour at 60°C, the air-dried fresh sections were deparaffinized (2x 5min fresh xylene, 2x 2min 100% ethanol at room temperature, in turn) and thoroughly dried in an oven for 5 min at 60°C. The slides were then treated with hydrogen peroxide for 10 min at room temperature. Following this, the slides were washed twice with distilled water before slowly immersing the slide rack into mild-boiled 1x co-detection target retrieval solution for 15 minutes (98-102°C). At the end of target retrieval, the hot slide rack was promptly transferred to 1x PBST (0.1% Tween-20). The slides were then incubated overnight at 4°C with primary antibody diluted in co-detection antibody diluent: anti-Ki67 antibody (SP6) (1:100, Abcam), anti-E-Cadherin antibody (1:50, BD Biosciences), anti-FABP2/I-

FABP antibody (1:100, R&D systems). Following primary antibody incubation and washing 3x 2min with PBST, the slides were applied post-primary fixation by submersion in 10% Neutral Buffered Formalin (NBF, VWR, Pennsylvania, USA) for 30 min at room temperature in a fume hood. Next, protease plus was applied for 30 min at 40°C in a HybEZ™ Oven, followed by two 2-minute washes with distilled water. The slides were then subjected to hybridization with 1:50 diluted *TNFSF13* (Cat# 406981-C2) in *FAS* (Cat# 427031) (or diluted in probe diluent if using only the *TNFSF13* probe), positive control (Cat# 320881) and negative control (Cat# 320871) probes for 2 hours at 40°C in a HybEZ™ Oven, respectively. After probe hybridization, the slides could be either stored in 5x SSC (Promega) overnight at room temperature or for next step after being rinsed with 1x wash buffer for 2 x 2 min. Subsequently, the slides were hybridized with a series of steps: AMP1 for 30 min, AMP2 for 30 min, AMP3 for 15 min, HRP-C1 signal for 15 min (for *FAS* probe), 1:750 diluted Opal™ 570 (Cat# FP1488001KT, Akoya Biosciences, Massachusetts, USA) fluorophore in RNAscope® Multiplex TSA buffer for 30 min, HRP blocker for 15 min, HRP-C2 signal for 15 min (for *TNFSF13* probe), 1:750 diluted Opal™ 690 (Cat# FP1497001KT, Akoya Biosciences) fluorophore in RNAscope® Multiplex TSA buffer for 30 min, HRP blocker for 15 min in a HybEZ™ Oven at 40°C, successively. Washes with 1x wash buffer twice for 2 min at room temperature were performed between hybridization steps. After the final wash step, the slides were incubated with specific fluorophore-conjugated secondary antibodies (AffiniPure IgG, Jackson ImmunoResearch, Pennsylvania, USA) from the same host as primary antibodies, diluted at 1:500 in co-detection antibody diluent, for 1 hour at room temperature. Following this, the slides were washed with 1x PBST 3x 2min at room

temperature and then counterstained with DAPI for 30s without washing and mounted with Prolong Gold antifade mounting medium (Thermo Fisher Scientific). The slides could be stored at 4°C in the dark for up to two weeks.

Click-iT™ Plus TUNEL Assay for In Situ Apoptosis Detection, Alexa Fluor™ 594 dye (Cat# 10618, Thermo Fisher Scientific) was employed to identify apoptosis on slides according to the manufacturer's protocol. Briefly, after baking for 1 hour at 60°C, tissue sections were deparaffinized/hydrated first (2× 5 min fresh xylene, 2x 5min 100% ethanol, 5 min 95% ethanol, 5 min 80% ethanol, 5 min 70% ethanol, 5min di H_2O at room temperature, in turn) and then applied for antigen retrieval for 15min in 10 mM citric acid in di H_2O (pH 6.0) using a 2100 antigen retriever (Aptum Biologics, Southampton, UK). After rinsing once for 5 min with diH_2O and 1xPBS respectively, the slides were fixed with 4% paraformaldehyde for 15 minutes at 37°C, permeabilized with Proteinase K solution at room temperature and fixed with another 4% paraformaldehyde for 5 minutes at 37°C, in turn. Between these steps, the slides underwent two 5-minute washes in PBS. In the final stages of the procedure, the slides were rinsed with deionized water, followed by a pretreatment step with 100 μL of TdT Reaction Buffer for 10 minutes at 37°C. Subsequently, the slides were incubated in 50 μL TdT reaction mixture (47 μL TdT Reaction Buffer, 1 μL EdUTP, 2 μL TdT enzyme) for 60 minutes at 37°C. Following being washed with deionized water, 3% BSA and 0.1% Triton™ X-100 (Sigma-Aldrich) in PBS, as well as 1x PBS for 5 min each, the slides were exposed to 50 μL of the Click-iT™ Plus TUNEL reaction cocktail (comprising 45 μL Click-iT™ Plus TUNEL Supermix, 5 μL 10X Click-iT™ Plus TUNEL Reaction Buffer Additive) for 30 minutes at 37°C, protected from light. Following this, the slides were washed with 3%

BSA in PBS and then 1x PBS for 5 minutes each. Moving forward, the slides were blocked with 3% BSA in PBS for 1 hour at room temperature protected from light before being subjected to an overnight incubation at 4°C with primary antibody (FABP2/I-FABP) diluted in blocking buffer. After being washed twice with 3% BSA in PBS, the slides were treated with fluorophore-conjugated secondary antibody diluted at 1:500 in blocking buffer for 1 hour and then counterstained with DAPI for 8 min at room temperature, protected from light. The slides finally were mounted with Prolong Gold antifade mounting medium and stored at 4°C in the dark.

All images were acquired eight hours later with Keyence BZ-X 800 all-in-one microscope and were further analyzed using software from the Keyence microscope, Adobe Photoshop, or Image J software. For quantification of copies of *TNFSF13* and *FAS* per cell in colonoids/organoids and colon tissue, the total number of red/green dots (representing positive signal) in each view were divided by total numbers of cells in the same view. At least 10 colonoids/organoids/crypts from 4-6 images for each sample were analyzed. For quantification of $TUNEL^{+}$ cells, more than 10 colonoids/views from 4-6 images were assessed for each sample. Detailed antibodies information was listed in Supplementary Table 2.

5-ethynyl-2'-deoxyuridine (EdU) assay. For EdU assay, appropriate colonoids, organoids and monolayers were pre-treated with 10 μM EdU in the respective media at day 7 (day 2 for monolayer) after plating. Following 2-hour EdU treatment, cells were collected and enzymatically digested to single cells, as described above. After DAPI staining and subsequently washing with 3 mL of 1% BSA in PBS, cell pellet was

collected and processed for EdU staining using Click-iT™ Plus EdU Alexa Fluor™ 647 Flow Cytometry Assay Kit (Thermo Fisher Scientific) according to the manufacturer's protocol. Briefly, the pellet was fixed with 100 µL of Click-iT™ fixative (Component D) for 15 minutes at room temperature, protected from light. After being washed with 3 mL of 1% BSA in PBS at the end of fixation, cells were then permeabilized in 100 µL of 1X Click-iT™ saponin-based permeabilization and wash reagent for additional 15 min in darkness on ice. Next, 500 µL of Click-iT™ Plus reaction cocktail (438 µL DPBS, 10 µL Copper protectant, 2.5 µL Fluorescent dye picolyl azide, 50 µL Reaction buffer additive for one sample) was added in cells without washing and incubated for 30 minutes at room temperature, protected from light. After being washed once with 3 mL of 1X Click-iT™ saponin-based permeabilization and wash reagent and resuspended in FACS buffer, the cells were analyzed with an LSR Fortessa analyzer (BD Biosciences) in the CHOP Flow Core.

Colonoids/organoids formation assay. For colonoids/organoids formation assay, cells were collected at day 7 after plating and digested with 0.05% trypsin (ThermoFisher Scientific, Massachusetts, USA) for 10 min at 37°C in a bead bath (final ratio as 10% FBS was added to de-activate trypsin). Single cells were further dissociated by pipetting up and down several times. 5,000 live cells (or 1,000 live cells for tissue-derived colonoids) were quantified by a Countess™ 3 FL Automated Cell Counter (ThermoFisher Scientific, Massachusetts, USA) and plated in 10 µL Matrigel per well in a 96-well plate with 100 µL of media. 10 µM Y-27632 was added for the first 2 (tissue-derived colonoids) or all (iPSC-derived colon organoids) days. Imaging and

quantification of live colonoids/colon organoids were performed on days 1-7 to monitor plating efficiency and growth using the Keyence BZ-X 700 all-in-one microscope with accompanying analysis software.

Co-immunoprecipitation (co-IP) and Western blotting. Human colonoids cells at day 7 after plating were collected as shown above and lysed in 1 mL of NP-40 lysis buffer (Thermo Fisher Scientific) for co-IP, or RIPA lysis buffer (Thermo Fisher Scientific) for regular protein samples, along with protease and phosphatase inhibitors for 30 minutes on ice with shaking at 10-minute intervals. The resultant supernatant was then transferred to a fresh tube placed on ice, following centrifugation at 10,000 × g for 10 minutes at 4°C or stored at -80°C for subsequent use. Next, for co-IP, 500μL of the lysate, containing antigen sample, was mixed with 10 μg of antibody of Human TNFSF13 Antibody (Cat# MAB8844, R&D Systems) or Mouse IgG2B Isotype Control (Cat# MAB004, R&D Systems) and incubated overnight at 4ºC with constant shaking. 25μL (0.25mg) per sample of Sera-Mag™ SpeedBeads Magnetic Protein A/G Particles (Cytiva) were prewashed in 175 μL and 1 mL of wash buffer (25 mM Tris, 0.65 M NaCl, 0.05% Tween-20 detergent- Promega, pH 7.5) with gently vortex to mix, in turn. The particles were subsequently collected using a magnetic stand. These pre-washed magnetic beads were introduced into the antigen sample/antibody mixture and incubated at room temperature for 1 hour with mixing. Following this incubation, the beads were collected with a magnetic stand and washed three times, each with 500μL of wash buffer. After the final wash, the beads were resuspended in 100μL of SDS-PAGE reducing sample buffer (GenScript, New Jersey, USA) and heated either at 96-

100ºC in a heating block for 10 minutes or at room temperature for 10 minutes with mixing if the samples were intended for Western blot with a rabbit antibody (primary or secondary). Subsequently, the beads were separated magnetically, and the supernatant containing the target antigen was carefully collected and stored at -20°C.

NuPAGE™ 4 to 12%, Bis-Tris, 1.0–1.5 mm, Mini Protein Gels (Thermo Fisher Scientific) were employed for detecting FAS protein in co-IP supernatant containing target antigen or other soluble proteins in colonoids/organoids according to the manufacturer's protocol. Briefly, appropriate samples and pre-stained protein marker (ApexBio, Texas, USA) were loaded into a NuPAGE mini Protein Gel and electrophoresis was then carried out with 1X NuPAGE® SDS Running Buffer (200 mL for the Upper Buffer Chamber and 600 mL for the Lower Buffer Chamber, Thermo Fisher Scientific) at a constant voltage of 200V for 50 min within a XCell SureLock™ Mini-Cell system (Thermo Fisher Scientific). Subsequently, proteins were transferred to a pre-soaked PVDF membrane (pre-soaking first with methanol for 30s and washing with water and then immersing in 2 x NuPAGE® Transfer Buffer for 5 min, Thermo Fisher Scientific) using Trans-Blot Turbo Transfer System (Bio-Rad, California, USA) for 30-60 min under standard conditions. After transfer, the membrane was blocked with 5% dry milk (Lab Scientific, New Jersey, USA) in TBST (0.1% Tween-20 in Cat# ab133619, Abcam, Cambridge, UK), anti-BCL-XL (Cat# 2762S, Cell Signaling Technology, Massachusetts, USA), anti-β-ACTIN (Cat# A5316-.2ML, Sigma-Aldrich) in blocking buffer overnight at 4°C on a shaker. After being washed three times for 10 min with TBST, the membrane was incubated with diluted Peroxidase (HRP) Anti-Rabbit IgG Goat Secondary Antibody (Cat# 7074S, Cell Signaling Technology), or Rabbit anti-

Mouse IgG (H+L) Secondary Antibody [HRP] (Abcam or Novus Biologicals, Colorado, USA), or Anti-mouse IgG VeriBlot for IP secondary antibody (Cat# ab131368, Abcam) in blocking buffer for 1 hour at room temperature. Protein detection was facilitated by a luminol-based detection reagent (Santa Cruz Biotechnology, Texas, USA). Subsequently, the membrane was imaged using a Gel Doc XR+ Gel Documentation System (Bio-Rad). Band intensity was measured by ImageJ software. Detailed antibodies information was listed in Supplementary Table 2.

RNA isolation and qRT-PCR. Total RNA was isolated with Quick-RNA™ Miniprep Kit (ZYMO RESEARCH) from human PBMCs, patient-derived colonoids and iPSC-derived organoids and then subjected to cDNA synthesis using random hexamers with TaqMan™ Reverse Transcription Reagents (Thermo Fisher Scientific) according to the manufacturer's instructions. Real-time quantitative PCR (qPCR) was performed either with the Power SYBR Green Master Mix or TAQMAN Fast Gene Expression Universal PCR Master Mix 2X (for Taqman probes) (Applied Biosystems) on QuantStudio 3 and/or 5 Real-Time PCR Systems (Thermo Fisher Scientific) with specific primers (Supplementary Table 5) according to the manufacture's protocol. GAPDH was used as an internal control for normalization. The relative gene expression levels were calculated using the formula $2^{-\Delta\Delta Cp}$ method. Microsoft excel and GraphPad Prism9 software were used to process the qPCR data.

Neutralization experiments. Experiments used 1 μg/mL TNFSF13 (Cat #MAB5860, R&D Systems), 10 μg/mL recombinant Human TNFSF13 (HEK293-expressed) protein (Cat

#5860-AP-010, R&D Systems), Mouse IgG1 Isotype Control (Cat # MAB002, R&D Systems), or 5 µg/mL FAS (clone ZB4, Cat #05-338, Millipore-Sigma, Massachusetts, USA) and Mouse IgG1 Negative Control (Millipore-Sigma) antibodies, or 5µg/mL recombinant human Fas ligand/TNFSF6 Protein (Cat#126-FL-010, R&D Systems) and Mouse IgG2B Isotype Control (Cat# MAB004, R&D Systems) in media. Media was changed every other day. For patient-derived colonoids, iPSC-derived organoids and differentiated memory B cells, neutralizing antibodies were introduced from day 0 and continued until the time of sample collection. Detailed antibodies information is listed in Supplementary Table 2.

ELISA. To evaluate secreted TNFSF13 protein, 300 clusters derived from all human colonoids, or 2000 clusters derived from iPSC-organoids lines were plated in 30 µL Matrigel and feed with 500 µL media in a 24-well plate with standard passage protocol. 3 wells with 500 µL media, devoid of organoids, were designated as negative control on the same plate, simultaneously. Cells were harvested at d4 post-seeding with the aforementioned protocol. To obtain both cytosolic and solubilized membrane and membrane-associated proteins from the same sample, the Mem-PERTM Plus Membrane Protein Extraction Kit (Thermo Fisher Scientific) was employed according to the manufacturer's protocol. Briefly, cell pellet was resuspended and incubated in 0.75mL of Permeabilization buffer with protease and phosphatase inhibitors (Thermo Fisher Scientific) for 10 minutes at 4°C with constant mixing after being washed with 3mL and 1.5mL of Cell Wash Solution successively, supplemented with protease and phosphatase inhibitors, followed by centrifugation at 300 × g for 5 minutes at 4°C. The

supernatant, containing cytosolic proteins, was collected and stored at -80°C after being centrifuged at 16,000 × g for 15 minutes at 4°C. Meanwhile, the resulting pellet was reconstituted in 0.5mL of Solubilization Buffer along with protease and phosphatase inhibitors and incubated at 4°C for 30 minutes with constant mixing. After centrifugation at 16,000 ×g for 15 minutes at 4°C, the supernatant, containing solubilized membrane and membrane-associated proteins, was transferred and stored at -80°C. Furthermore, media that contained secreted proteins were collected and stored at -80°C following centrifugation at 300 × g for 5 minutes at 4°C. For B cell differentiation assays, 3000 cell clusters derived from human colonoid lines were plated in 45 µL Matrigel and fed with 500 µL media in a 24-well plate. Conditioned media containing secreted proteins were collected at day 4 post-seeding or day 9 post-seeding and stored at -80°C following centrifugation at 300 × g for 5 minutes at 4°C, respectively.

The Human APRIL/TNFSF13 DuoSet® ELISA Development System (Cat# DY884B) was employed to measure the expression level of TNFSF13 in colonoids/iPSC-organoids or media according to the manufacturer’s protocol. Briefly, the sealed plate was incubated with 100 µL per well of the diluted Capture Antibody overnight at room temperature and washed three times with 400 µL per well of Wash Buffer. After blocking with 300 µL of Reagent Diluent per well for a minimum of 1 hour at room temperature, the washed plate was treated with 100 µL per well of either sample or standards in Reagent Diluent and incubated for 2 hours at room temperature with an adhesive strip covering. The standard was reconstituted in Reagent Diluent with a concentration gradient (2000 pg/mL, 1000 pg/mL, 500 pg/mL, 250 pg/mL, 125 pg/mL, 62.5 pg/mL, 31.3 pg/mL, 0 pg/mL). Then, the plate was incubated with 100 µL of the

Detection Antibody for 2 hours, followed by 100 µL of the working dilution of Streptavidin-HRP for 20 min, and 100 µL of Substrate Solution for 20 min, in turn. The plate was washed 3 times with 400 µL per well of Wash Buffer between these steps. At the end of the final wash, 50 µL of Stop Solution was added to each well and mixed thoroughly by gently tapping the plate. The optical density (O.D.) of each well was determined promptly under 450 nm with wavelength correction setting at 570nm, using a GloMax®-Multi Detection System (Promega, Wisconsin, USA) or Varioskan LUX multimode microplate reader. The corrected O.D. is the reading at 450 nm subtract readings at 570 nm. Data processing and analysis used a four-parameter curve fit (4PL) online tool (https://www.aatbio.com/tools/four-parameter-logistic-4pl-curve-regression-online-calculator) and the equation form was created as follows based on the standard curve (Y=absorbance O.D., X=concentration of TNFSF13 in pg/mL):

$$Y = Min + \frac{Max - Min}{1 + \left(\frac{X}{inflection\ Point}\right)^{Hill\ coefficient}}$$

To evaluate secreted IgA, IgG and IgM protein, media was collected at day 14 post-seeding from differentiated memory B cells and stored at -80°C following centrifugation at 2000 × g for 10 minutes at 4°C. In parallel, 3 wells containing 150 µL media, devoid of cells, were designated as negative control on the same plate. The Human IgA ELISA Kit (Cat# ab196263), Human IgG (Total) ELISA Kit (Cat# BMS2091) and Human IgM ELISA Kit (Cat# BMS2098) were used according to the manufacturer's protocol. Briefly, 50 µL of diluted samples (1:1 into Sample Diluent NS) and/or standard were added to appropriate wells of SimpleStep Pre-Coated 96-Well Microplate. Two blank wells were used as the zero control. Each sample was assayed with two technical

replicates. After adding another 50 µL of the Antibody Cocktail to each well, the plate was sealed and incubated for 1h at room temperature on a plate shaker set to 400 rpm. Following a wash step with 3 x 350 µL 1X Wash Buffer PT, 100 µL of TMB Development Solution was added to the washed plate and incubated for 10 minutes in the dark on a plate shaker set to 400 rpm. At the end of the incubation, 100 µL of Stop Solution was added, and absorbance at 450 nm wavelength measured using a GloMax®-Multi Detection System (Promega) or Varioskan LUX multimode microplate reader. The concentration was determined as described above.

Due to the variation in the percentage of B cells and memory B cells from each donor, we seeded 67,000 -150,000 human memory cells per well in our assays. As such, the absolute values of ELISA between two assays were not directly comparable. However, we ensured equal cell numbers between control and case in each assay for meaningful comparisons.

Single cell RNA sequencing (scRNA-seq) preprocessing and analysis.

Sample preparation. Human colonoids (2 control, 2 VEO-IBD, 1 variant with 2 replicates from different passages) were collected and digested into single cells as shown above. The cell pellet was resuspended and incubated in 45 µL of FACS buffer and 5 µL of Human TruStain FcX™ Fc Blocking reagent (BioLegend) for 10 min at 4°C. Then, 1 µg of TotalSeqtrade-B0251 anti-human Hashtag 1 Antibody (BioLegend) was introduced for untreated samples (TotalSeqtrade-B0252 anti-human Hashtag 2 Antibody for TNFα-treated sample, TotalSeqtrade-B0253 anti-human Hashtag 3 Antibody for IFN-γ treated sample and TotalSeqtrade-B0254 anti-human Hashtag 4

Antibody for Trail treated sample) in FACS buffer (up to 50 µL) and incubated for another 30 min at 4°C after centrifuging the antibody pool at 14,000 x g for 10 minutes at 4°C. Afterward, the cells were washed three times with 3 mL FACS buffer, centrifuged at 4°C for 5 minutes at 300 x g, and then incubated with DAPI before being sorted in 1xDPBS+0.04% BSA with a MoFlo Astrios sorter (Beckman Coulter) or FACSAria Fusion Sorter (BD Biosciences) in CHOP Flow Core, as shown above, to isolate live single cells. Equivalent cell number from sorted untreated and treated samples were combined to create a sample pool for library construction. Only data from untreated samples was used in this study.

For fresh tissue from human colon biopsy, colonic biopsies were collected during colonoscopy and placed into Eppendorf tubes containing 1mL of collection medium (comprising 1% Pen/Strep and 1x HEPES and 1x GlutaMAX in Advanced DMEM/F12). These samples were immediately transported to the lab for processing or cryopreservation. For isolation of crypts and lamina propria, the biopsies were washed twice with 1mL of Chelation buffer before incubating in 1mL Chelation buffer along with 2 mM EDTA for 15min at 4°C with rotation. The tissue was then vortexed using a vortex mixer (Scientific Industries) at the maximum speed setting for 5 cycles, with 30 seconds of vortexing followed by a 30-second rest period on ice. After vortexing, the tissue, along with the supernatant, was transferred into a cold 35 mm dish with the luminal side (epithelium) facing upward. The crypts were meticulously scraped off the tissue using two Dumont SS forceps (Angled) under a Stereo microscope (Aven tools). The crypts contained in solution were then collected into a 15 mL conical and spun down at 700x g for 1min, followed by digestion steps in 1.5 mL TrypLE along with 0.5 U/mL DNAse I

(Roche, Basel, Switzerland) for 30 min at 37°C with 800 rpm using a ThermoMixer (Thermo Fisher Scientific). After deactivation with 10%FBS and centrifugation at 700x g for 3min, the epithelial cells were reconstituted in 500 µL of 1%BSA in 1x HBSS, supplemented with 0.5 U/mL of DNAse I. Meanwhile, following rinsing twice with 5% FBS in cold DMEM/F12 (the solution was collected in the crypts collection conical), the lamina propria fraction was digested in 500 µL of stroma dissociation enzyme mix, comprising 0.13 WU/mL of Liberase-TH (Cat #5401135001, Sigma-Aldrich), 0.5U/mL DNAse I in 1x HBSS, for 30 min at 37°C with 800 rpm using a ThermoMixer. The resulting solution was then passed through a 40 µm strainer placed in a 50mL conical to collect supernatant. After two additional cycles, the remaining fragments was additionally forced through the strainer using a plunger from a 1 mL insulin syringe. A wash with 4 mL of 5% FBS in cold DMEM/F12 was performed to collect any remaining cells from the strainer in the same conical. Following centrifugation at 700x g for 3min, the lamina propria cells was reconstituted in 500 µL of 5% FBS in cold DMEM/F12. Cell Gibco, Massachusetts, USA) exclusion using a Countess™ 3 FL Automated Cell Counter (Thermo Fisher Scientific). If cell viability was lower than 70%, the Dead Cell Removal kit (Cat #130-090-101, Miltenyi Biotech) was employed following the manufacturer’s instructions. After viability checks, the cells were ready for subsequent library construction steps.

Library preparation. Chromium Next GEM Single Cell 3' Reagent Kits v3.1 with Feature Barcoding technology for Cell Surface Protein (10X GENOMIC, California, USA) were employed for library construction according to the manufacturer’s protocol. Briefly, a total of 43.2 µL of 10,000 cells diluted in nuclease-free water (Thermo Fisher

Scientific) (viability ≥90% for colonoid samples and ≥71% for biopsy samples) from above sample pool were combined with 31.8 µL Master Mix (comprising 18.8 µL RT Reagent B, 2.4 µL Template Switch Oligo, 2.0 µL Reducing Agent B, 8.7 µL RT Enzyme C) and transferred to an assembled Chromium Next GEM Chip G, which was filled with Gel Beads or 50% Glycerol (VWR), and the setup was processed in a Chromium Controller (10X GENOMIC) for ~18 min. Upon completion of the run, 100 µL of the Gel Beads-in-emulsion (GEMs) was carefully transferred to a new tube and incubated in a thermal cycler (53°C 45min, 85°C 5min, 4°C). Followed by recovery using 125 µL recovery agent for 2 min, the remaining GEMs were incubated with Dynabeads Cleanup Mix (182 µL Cleanup Buffer, 8 µL Dynabeads MyOne SILANE, 5 µL Reducing Agent B, 5 µL Nuclease-free Water) for 10 min at room temperature and then eluted with Elution Solution I (98 µL Buffer EB, 1 µL 10% Tween 20- Bio-Rad, 1 µL Reducing Agent B) following the standard steps as shown in protocol. 35 µL sample was further processed for cDNA Amplification by mixing with 50 µL Amp mix and 15 µL Feature cDNA Primers 2 in a thermal cycler (98 ºC 3min, 98 ºC 15s, 63 ºC 20s, 72 ºC 1min, 10 cycles, 72 ºC 1min, 4 ºC). The resulting cDNA was then subjected to Pellet Cleanup (for 3' Gene Expression library) and Supernatant Cleanup (for Cell Surface Protein library) with SPRIselect reagent (Beckman Coulter), following standard steps as shown in the protocol. Concentration of the samples obtained from Pellet Cleanup were determined using a Qubit 4 Fluorometer (Thermo Fisher Scientific) and Qubit dsDNA HS Assay Kit (Thermo Fisher Scientific) for subsequent Sample Index PCR step (with concentrations of 25-30ng/µL for Pellet Cleanup samples and 3-10ng/µL for Supernatant Cleanup samples). Next, 10 µl of purified cDNA sample obtained from Pellet Cleanup were

subjected to Fragmentation, End Repair & A-tailing by mixing with 25 µL Buffer EB (Qiagen) and 15 µL Fragmentation Mix (comprising 5 µL Fragmentation Buffer and 10 µL Fragmentation Enzyme) in a thermal cycler (4 ºC, 32 ºC 5min, 65 ºC 30min, 4 ºC) and Post Fragmentation, End Repair & A-tailing Double-Sided Size Selection using SPRIselect reagent with steps as shown in the protocol. Following size selection, 50 µL of resulting samples were subjected to Adaptor Ligation by mixing with 50 µL Adaptor Ligation Mix (20 µL Ligation Buffer, 10 µL DNA Ligase, 20 µL Adaptor Oligos) in a thermal cycler (20 ºC 15min, 4 ºC) and Post Ligation Cleanup using SPRIselect reagent following the protocol. The samples were then subjected to Sample Index PCR by mixing 60 µL Sample Index PCR Mix (50 µL Amp mix, 10 µL SI Primers) in 30 µL sample and 10 µL of an individual Single Index for each well (recording the well ID that was used) in a thermal cycler (98 ºC 45s, 98 ºC 20s, 54 ºC 30s, 72 ºC 20s, 11 cycles, 72 ºC 1min, 4 ºC) and Post Sample Index PCR Double Sided Size Selection using SPRIselect reagent following the protocol. Finally, 35 µL samples of 3' Gene Expression library can be stored at -20°C for long-term storage or for sequencing. For Cell Surface Protein Library Construction, 5 µL of DNA sample obtained from the Transferred Supernatant Cleanup step was subjected to Sample Index PCR by mixing with Sample Index PCR Mix (50 µL Amp mix, 35 µL Feature SI Primers 2) and 10 µL of an individual Single Index to each well (record the well ID that was used) in a thermal cycler (98 ºC 45s, 98 ºC 20s, 54 ºC 30s, 72 ºC 20s, 9 cycles, 72 ºC 1min, 4 ºC) and Post Sample Index PCR Double Sided Size Selection using SPRIselect reagent, following the protocol. At the end, 40 µL samples of 3' Gene Expression library can be stored at -20°C for long-term storage or for sequencing. Both the libraries for 3' Gene Expression

and Cell Surface Protein were subsequently submitted to CHOP Center for Applied Genomics for sequencing using NovaSeq platform (Illumina, California, USA).

Preprocessing and annotation of single-cell RNA-Seq data- colonoids. Fastq files were generated using 10X Genomics Cell Ranger v6.0.0.0 then aligned to the GRCh38 human reference genome. HTO data was normalized using the centered log ratio transformation then hashtag demultiplexing of the pooled samples was performed using the HTODemux algorithm, with default settings, in Seurat v4.3.0(1). Singlets were then extracted and used for downstream analyses. Seurat was used for quality control, sample normalization, dimensional reduction, data integration, clustering, and visualization. First, cells containing <200 genes or having >25% mitochondrial gene expression were removed. The R package DoubletFinder(2) was then used to remove doublets that had not been previously detected. Data was normalized and variance stabilized using SCTransform v2 prior to integration of the different scRNA-seq datasets. To identify cell clusters, PCA was performed followed by nearest-neighbor graph construction using the first 40 principal component dimensions. Clusters were then identified using the FindClusters algorithm set to a resolution of 0.4. Clusters were annotated using markers identified with the FindAllMarkers function as well as manual visualization of known cell markers from the literature. Clusters with fewer than 5 DEGs (min.diff.pct = 0.25) were merged (Supplementary Table 3-4).

Preprocessing and analysis of biopsy single-cell RNA-Seq data. Epithelial and stromal samples were analyzed separately. Fastq files were generated as described above. Ambient RNA was removed using SoupX v1.6.2(3). The adjusted counts were then converted to Seurat objects and similar QC and processing steps were applied as

described above, with the exception for mitochondrial cutoffs, which were set to 85% and 30% for epithelial and stromal samples, respectively. For the epithelial samples, major clusters were identified at a resolution of 0.1. Epithelial cells were then extracted and samples were re-integrated and processed. Initial clustering identified 15 clusters including 1 mitochondrial, 1 ribosomal and 1 stress cluster, which were excluded from further analysis. For the stromal samples, major clusters were identified at a resolution of 1.1. Five B cell clusters were identified within the data and were extracted for further analysis. Samples were re-integrated and processed. Final clustering revealed 10 subclusters including 3 B cell and 7 plasma cell clusters.

Differential expression analysis. For colonoid data, we performed a combined differential expression analysis by comparing cells from all clusters across the different phenotypes. DEGs were identified using the FindMarkers function in Seurat (min.diff.pct = 0.1). We used clusterProfiler v4.7.1.1(4) to identify significantly enriched KEGG pathways(5) and biological processes using the Benjamini-Hochberg adjusted p-value for multiple test correction set to <.05. Data are deposited in GEO. The accession number is GSE243445.

We evaluated 4,805 and 4,277 cells, respectively, from 2 independent healthy control and 2 TNFSF13 wild type VEO-IBD colonoid lines, and 4,682 cells from 2 different passages of TNFSF13 variant colonoids.

Imaging Mass Cytometry (IMC). Formalin-fixed, paraffin embedded tissue sections from a total of 7 patient samples (3 controls, 3 VEO-IBDs, and 1 TNFSF13 variant with 2 replicates) were subjected to analysis using IMC. Tissue sections were stained with a

metal-conjugated antibody cocktail using previously published methods(6) (Supplementary Table 5). We acquired 1-3 regions of interest (ROIs) per tissue section, resulting in a total of 13 images for quantitative analyses. *Cell segmentation*: For cell segmentation, we used the pixel classification feature in Ilastik software(7) and trained the software to distinguish nuclear signals from the background, using the Iridium-191 channel of each IMC image. Probability maps from the nuclear pixel classification step were extracted to segment the nuclei using Cell Profiler software (8). The nuclear segmentation was expanded 6 pixels outward to approximate cell surface. Cell segmentation masks were extracted for performing cell annotation.

Cell annotation: Cell annotation was performed using the object classification feature in Ilastik. First, cells were categorized into the following major cell categories: T cells ($CD3^+$), B cells/PCs ($CD20^+$/$CD27^+$/$CD38^+$), dendritic cells ($CD11b^+$), macrophages ($CD68^+$), and monocytes ($CD14^+$). Once these major cell type predictions were exported, we performed object classification again to subcategorize T cells into $CD4^+$ T cells, $CD8^+$ T cells, $Foxp3^+$ regulatory T cells, and $CD3^+$ T cells, and to distinguish between B cells ($CD20^+$) and PCs ($CD20^-$/$CD27^+$/$CD38^+$). We exported csv files containing the probability data for each cell, which was used to annotate the cells into 10 cell populations: $CD3^+$ T cells, $CD4^+$ T cells, $CD8^+$ T cells, regulatory T cells, B cells, PCs, myeloid cells, dendritic cells, macrophages, and “non-immune” cells. A csv file containing the mean intensity of each channel for each cell after passing through a 3x3 pixel median filter was exported from Cell Profiler.

Data filtering and analysis: All IMC analyses were performed using R version 3.6.3. The E-cadherin channel was used to create epithelial tissue masks. Using the

EBImage package in R, we categorized all cells based on their closest distance to the epithelium. All cells that were annotated as non-immune cells and were 0 μM from the epithelium were annotated to be epithelial cells, and all other non-immune cells were annotated as stromal cells. All cells that were >75 μm from the epithelium were excluded from the study to focus our analysis on cells that were in close proximity to the epithelium (Figure 5D, Supplementary Figure 10A). Furthermore, we used EBImage to manually remove all lymphoid tissues and submucosal tissues from downstream analysis (Figure 5D-E, Supplementary Figure 10B-C). The number of cells analyzed from each IMC image after filtering is detailed in Supplementary information, Table S5.

IMC identified 9 major immune cell populations within colon sections from 7 patients (3 controls, 3 TNFSF13 wild type VEO-IBD, and 1 TNFSF13 variant with 2 different biopsies): $CD3^+$ T cells, $CD4^+$ T cells (T helper cells), $CD8^+$ T cells (cytotoxic T cells), $FOXP3^+$ regulatory T cells (Tregs), B cells, PCs, myeloid cells, dendritic cells, and macrophages (Figure 5D, Supplementary Figure 10A-B and Table 5). Because IMC retains the X and Y coordinates of each cell in each image, we were able to assess immune cell composition with spatial resolution.

Flow cytometry. For human PBMCs, Ficoll-Paque Plus (GE Healthcare, Illinois, USA) was employed to isolate PBMCs from whole blood according to the manufacturer's protocol. Zombie Aqua was used to distinguish live cells. B cells were identified by physical characteristics and CD19 expression, and subsets were further identified by additional antibodies: IgM, IgD, CD27, CD38, CD80 and CD21. T cells were taken from physical gate followed by CD3, and subsets were further determined using CD4, CD8,

CD45RA, CXCR5, ICOS, CD25, CD161, TCR V alpha 7.2 and TCR V alpha24x18. ILCs were determined using physical lymphocyte gate and alive cells from DAPI negative. Followed by CD45 and lineage negative (CD19, CD3, CD1a, CD11c, CD14, CD34, CD123, BDCA2, and FceRI). Next is CD127 positive. CRTH2 negative is used later to determine ILC1 and ILC3 in combination with cKit and NKp44. CRTH2 positive is ILC2. Monocytes were visualized from first the physical gate followed by CD14. CD16 was analyzed from the lymphocyte, monocyte, and neutrophil physical gates. Analysis was carried out on an LSR Fortessa analyzer (BD Biosciences) in the CHOP Flow Core.

For colonoids and organoids, appropriate cells at day 7 post-plating were collected and recovered from Matrigel. After being digested in 0.05% trypsin (Thermo Fisher Scientific) for 10 min in 37°C bead bath (final concentration of 10% FBS was added to de-activate trypsin), cells were dissociated by pipetting up and down several times to achieve a single-cell suspension. After centrifugation at 300xg at 4°C, cell pellet was resuspended and incubated in FACS buffer (2% FBS in DPBS) with various antibodies for 30 min in dark on ice: Apc anti-human CD267 (TACI) (1A1) (BioLegend), Brilliant Violet 421™ anti-human CD269 (BCMA) (BioLegend), FITC anti-CD95 Mouse Monoclonal Antibody (clone: DX2) (BioLegend), PE anti-HVEM (TR2) Mouse Monoclonal Antibody (clone: 122) (BioLegend). DAPI (Sigma-Aldrich) was added at a final concentration of 0.1 μg/mL for an additional 10 min. Subsequently, samples were analyzed on an LSR Fortessa analyzer (BD Biosciences) in the CHOP Flow Cytometry Core, following washing with 3 mL FACS buffer. Alternatively, propidium iodide (PI, Thermo Fisher Scientific) was added at a final concentration of 1 μg/mL after washing until analysis.

To validate the efficacy of flow antibodies for TNFSF13 candidate receptors, human PBMCs isolated from whole blood by Ficoll-Paque Plus (GE Healthcare) was used with the same panels. For monolayers, cells were collected around on d8 post-plating and digested with 100 µL TrypLE™ Express Enzyme (Thermo Fisher Scientific) (de-activated using an equal volume of 10% FBS in advanced DMEM/F12) at 37°C for 5 min. Single cells were resuspended in FACS buffer and performed with the same protocol and panels. Detailed antibodies information was listed in Supplementary Table 2.

Protein–Protein Docking Analysis. Docking analyses were conducted using the HDOCK online server (http://hdock.phys.hust.edu.cn/), an integrated protein–protein docking platform based on a hybrid strategy combining template-based modeling and free docking (9). The protein structures or amino acid sequences of human TNFSF13 (UniProt ID: O75888), TNFSF6 (UniProt ID: Q53ZZ1), and FAS (UniProt ID: P25445) were retrieved from the UniProt database. FAS was designated as the receptor protein, while FASL and TNFSF13 were used as ligand proteins for separate docking analyses. For each protein pair, HDOCK generated multiple docking conformations, which were ranked according to the HDOCK docking score reflecting predicted binding affinity. The top-ranked docking models were selected for subsequent analysis. Interacting amino acid residues were identified based on a distance cutoff of ≤ 5 Å, as defined by the HDOCK interaction analysis. Two-dimensional representations of protein–protein interactions, including hydrogen bonds and hydrophobic contacts, were generated using LigPlot+ software. Three-dimensional visualization and structural analysis of docking

complexes were performed using PyMOL (version 4.3.0), enabling the identification and labeling of key interacting residues at the binding interface.

Protein-protein binding affinities and competitive binding analysis. Surface Plasmon Resonance (SPR) was used to determine the binding affinities between TNFRSF6 (FAS) and its ligands TNFSF13 and FASL (TNFSF6), as well as to evaluate their competitive binding to TNFRSF6. SPR experiments were performed using a Biacore 1K SPR system (Cytiva, Uppsala, Sweden) at 25 °C. Recombinant human FAS protein was immobilized on a CM5 sensor chip (Cytiva) using standard amine-coupling chemistry in accordance with the manufacturer's instructions. Briefly, the sensor surface was activated with a mixture of 37.5 mg/mL N-ethyl-N′-(3-dimethylaminopropyl) carbodiimide (EDC) and 5.75 mg/mL N-hydroxysuccinimide (NHS), followed by immobilization of FAS in 10 µg/mL in 10 mM Sodium acetate, pH4.0 (Cytiva) at the rate of 10 µL/min for 420s. Remaining active sites were blocked with 1 M Ethanolamine at the same flow

For affinity measurements, startup solution (1x running buffer—0.05% Tween20 in PBS, pH 7.2-7.4) was injected over sensor surfaces for 2 h. Then, 20 µg/mL recombinant TNFSF13 or FASL were injected over the TNFRSF6-coated surface at the rate of 30 µl/min for 90s in 1x running buffer. Association and dissociation phases (dissociation time is 600s) were recorded for each analyte concentration.

To assess competitive binding of TNFSF13 and FASL to TNFRSF6, ternary interaction and competitive binding assays were performed using the A–B–A injection program implemented in the Biacore SPR system:

1) FASL–APRIL Competition Assay. FASL was defined as analyte A, and TNFSF13 was defined as analyte B. Recombinant human FAS was immobilized on a CM5 sensor chip as described above. FASL at a series of concentrations (100, 10, 1, and 0 nM) was first injected over the TNFRSF6-coated sensor surface. The binding responses obtained from these injections were recorded and used as background signals corresponding to FASL binding alone. TNFSF13 at a fixed concentration of 100 nM was pre-incubated with FASL at identical final concentrations (100, 10, or 1 nM). The resulting mixtures were then injected over the TNFRSF6-immobilized surface, and the binding responses were recorded to assess the effect of FASL on TNFSF13 –FAS interaction. For the control group, TNFSF13 (100 nM) without prior incubation with FASL (0 nM FASL) was injected over the sensor surface to measure TNFSF13 –FAS binding in the absence of competition.
2) TNFSF13–FASL Competition Assay. In a reciprocal competition assay, TNFSF13 was designated as analyte A, and FASL as analyte B. TNFSF13 at different concentrations (500, 50, 5, and 0 nM) was first injected over the TNFRSF6-coated surface, and the resulting binding responses were recorded as background signals for TNFSF13 alone. For the experimental group, FASL at a fixed concentration of 100 nM was pre-incubated with TNFSF13 at matching final concentrations (500, 50, or 5 nM). The mixtures were subsequently injected over the TNFRSF6-immobilized surface to evaluate the impact of TNFSF13 on FASL–FAS binding. For the control group, FASL (100 nM) incubated without TNFSF13 (0 nM TNFSF13) was injected over the FAS surface as the reference condition.

A concentration-dependent reduction in binding responses compared with the respective control groups was interpreted as evidence of competitive binding between TNFSF13 and FASL for TNFRSF6. The SPR data/ sensorgrams were fitted using both steady-state affinity fitting (Steady State Affinity Fit Model) and kinetic fitting based on a 1:1 Langmuir binding model (1:1 Binding Model Kinetics Fit) to derive equilibrium dissociation constants (K_D) as well as association (k_on) and dissociation (k_off) rate constants. Sensorgrams were reference-subtracted and analyzed using Biacore Evaluation Software, and all experiments were independently repeated at least three times.

FAS expressing and cytokines treatment in cell lines. An FAS-expressing HEK293T cell line was generated using a transient eukaryotic expression lentiviral vector, PLV3-CMV-FAS-EGFP-puro, which was constructed to express the full-length coding sequence (CDS) of human FAS (NM_000043.6). HEK293T cells were transfected using Lipofectamine 2000 (Thermo Fisher Scientific) in Opti-MEM reduced-serum medium (Thermo Fisher Scientific) according to the manufacturer’s instructions. Following transfection, cells were subjected to puromycin selection for 3 days to enrich FAS-expressing cells. After selection, cells were treated with 100ng/mL IgG, rTNFSF13 for 2h and then fixed and subjected to immunofluorescence staining to assess ligand binding, as described above.

The Jurkat T cell line was used to assess apoptosis after 2h-treatment with 100ng/mL IgG, rTNFSF13 and rFASL, respectively. Apoptosis was evaluated by flow cytometry with an APC Annexin V Apoptosis Detection Kit with PI (Biolegend) according

to the manufacturer's instructions. Flow cytometry data were acquired as described above. In parallel, apoptosis-related signaling was further assessed by Western blot analysis, as described above.

Statistical analyses. Three healthy control and three VEO-IBD human colonoid lines from distinct patients were used as biological replicates. Three replicates with different passages from a single *TNFSF13* variant human colonoid line, and one WT and one variant iPSC-derived colon organoid line were used for statistical analyses. Statistical analysis of qPCR, flow cytometry analyses, organoids formation assay and quantification of positive cells in immunostaining assay were performed using two-tailed Student's t-tests, one-way ANOVA, two-way ANOVA or multi-comparison with Prism GraphPad or Microsoft Excel, and a significance threshold of $P<.05$ was utilized to determine statistical significance. Error bars denote mean ± standard deviation. The schematics were created with BioRender.com.

Data availability. Single cell RNA sequencing data will be deposited on a publicly available database. All other data are available from the corresponding author upon request.

SUPPLEMENTARY METHODS REFERENCES

1. Hao Y, Hao S, Andersen-Nissen E, Mauck WM, 3rd, Zheng S, Butler A, et al. Integrated analysis of multimodal single-cell data. *Cell.* 2021;184(13):3573-87 e29.
2. McGinnis CS, Murrow LM, and Gartner ZJ. DoubletFinder: Doublet Detection in Single-Cell RNA Sequencing Data Using Artificial Nearest Neighbors. *Cell Syst.* 2019;8(4):329-37 e4.
3. Young MD, and Behjati S. SoupX removes ambient RNA contamination from droplet-based single-cell RNA sequencing data. *Gigascience.* 2020;9(12).
4. Wu T, Hu E, Xu S, Chen M, Guo P, Dai Z, et al. clusterProfiler 4.0: A universal enrichment tool for interpreting omics data. *Innovation (Camb).* 2021;2(3):100141.
5. Kanehisa M, and Goto S. KEGG: kyoto encyclopedia of genes and genomes. *Nucleic Acids Res.* 2000;28(1):27-30.
6. Kondo A, Ma S, Lee MYY, Ortiz V, Traum D, Schug J, et al. Highly Multiplexed Image Analysis of Intestinal Tissue Sections in Patients With Inflammatory Bowel Disease. *Gastroenterology.* 2021;161(6):1940-52.
7. Sommer C, Straehle, C., Kothe, U., and Hamprecht, F.A. . Ilastik. Interactive learning and segmentation toolkit. *In 2011 8th IEEE International Symposium on Biomedical Imaging (IEEE).* 2011;pp. 230–233.
8. Kamentsky L, Jones TR, Fraser A, Bray MA, Logan DJ, Madden KL, et al. Improved structure, function and compatibility for CellProfiler: modular high-throughput image analysis software. *Bioinformatics.* 2011;27(8):1179-80.

9. Yan Y, Tao H, He J, and Huang SY. The HDOCK server for integrated protein-protein docking. *Nat Protoc.* 2020;15(5):1829-52.

SUPPLEMENTARY FIGURE LEGENDS

SUPPLEMENTARY FIGURE 1

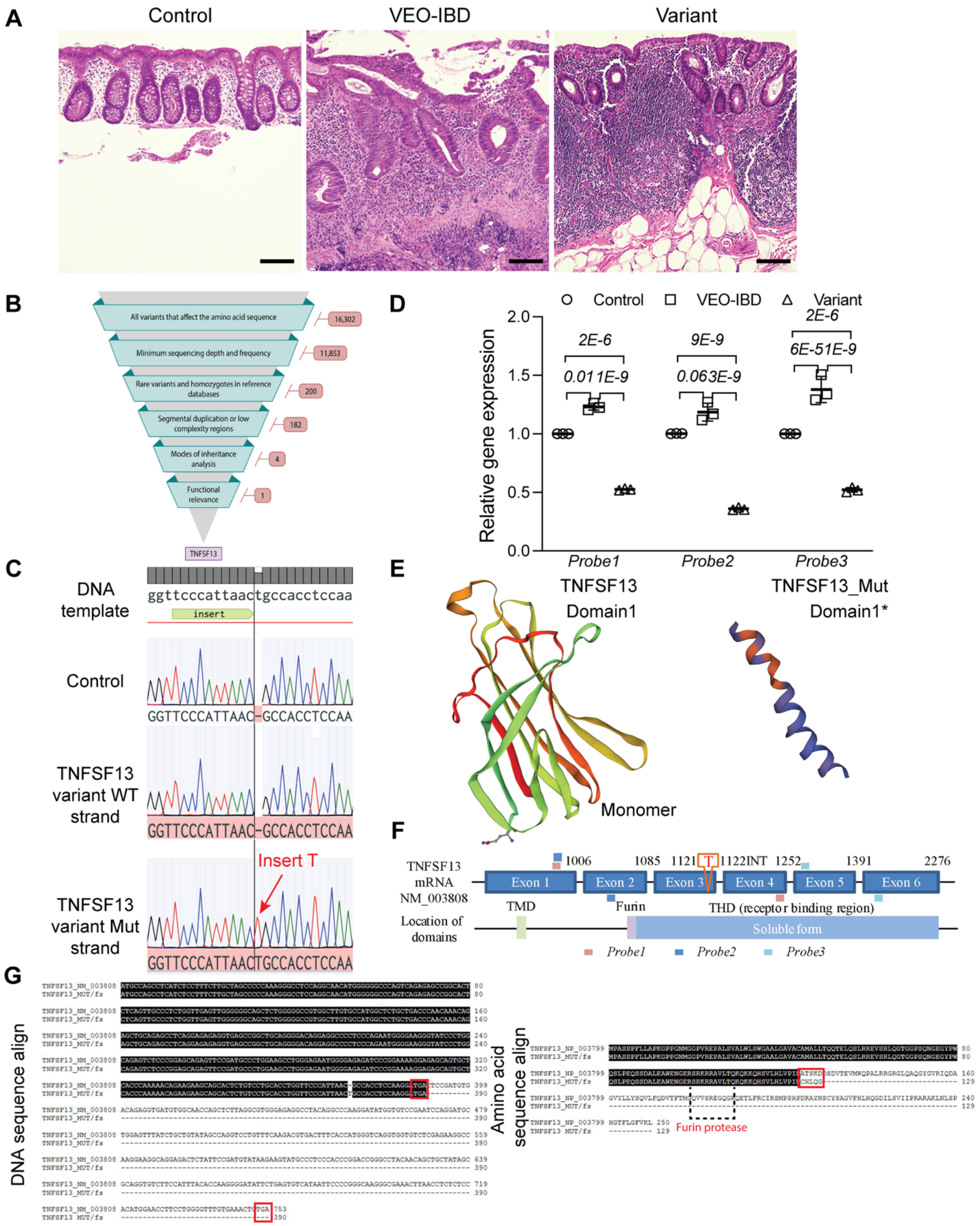

Supplementary Figure 1 related to Figure 1. Mutation of TNFSF13 in variant both in patient-derived colonoids and iPSC-organoids. (A) Representative H&E images of colon from control, VEO-IBD and variant. Scale bar: 100 μm. n=3 different patients for Control and VEO-IBD, n=3 slides from different blocks for variant**. (B)** Diagram for filtration strategy of the whole exome sequencing. **(C)** Sanger sequencing for TNFSF13 in variant and healthy control after TOPO TA clone with colonoids, iPSC-organoids and PBMC cDNA. Red arrowhead denoted T insert in the mutant strand. Three control colonoid lines and 3 independent passages of variant colonoids were used. **(D)** qPCR for *TNFSF13* with different probes from different location in *TNFSF13* mRNA in colonoids from control, VEO-IBD and variant. Location of the probes is shown in **(F)**. n=3 lines of colonoids from 3 different patients for Control and VEO-IBD, n=3 passages of colonoids for variant. **(E)** Images for 3D structure of TNFSF13 and predicted structure of TNFSF13 variant monomer protein. **(F)** Schematic for location of qRT-PCR probes in TNFSF13 mRNA and corresponding domains of TNFSF13 protein. *Probe1* contains the inserted site; *Probe2* is from the upper stream of the inserted site; *Probe3* is from the downstream of the inserted site. Red T denotes the insert of TNFSF13 variant. The size of exons/introns (thin line)/untranslated portions of the exons (UTR) and domains are not shown to scale. **(G)** Alignment of DNA (left) and amino acid sequences (right) of TNFSF13 and TNFSF13 variant sequence (c.372_373 T ins, NM_003808). Red box denotes stop codon (left) or new truncated amino acid sequence for variant (right). Dash line denotes the Furin protease recognizing site in TNFSF13 protein. *P* value as shown in the bar graphs unless *P*>0.05. Two-way ANOVA (with multiple comparisons) was used for statistical analysis in **(D)**.

SUPPLEMENTARY FIGURE 2

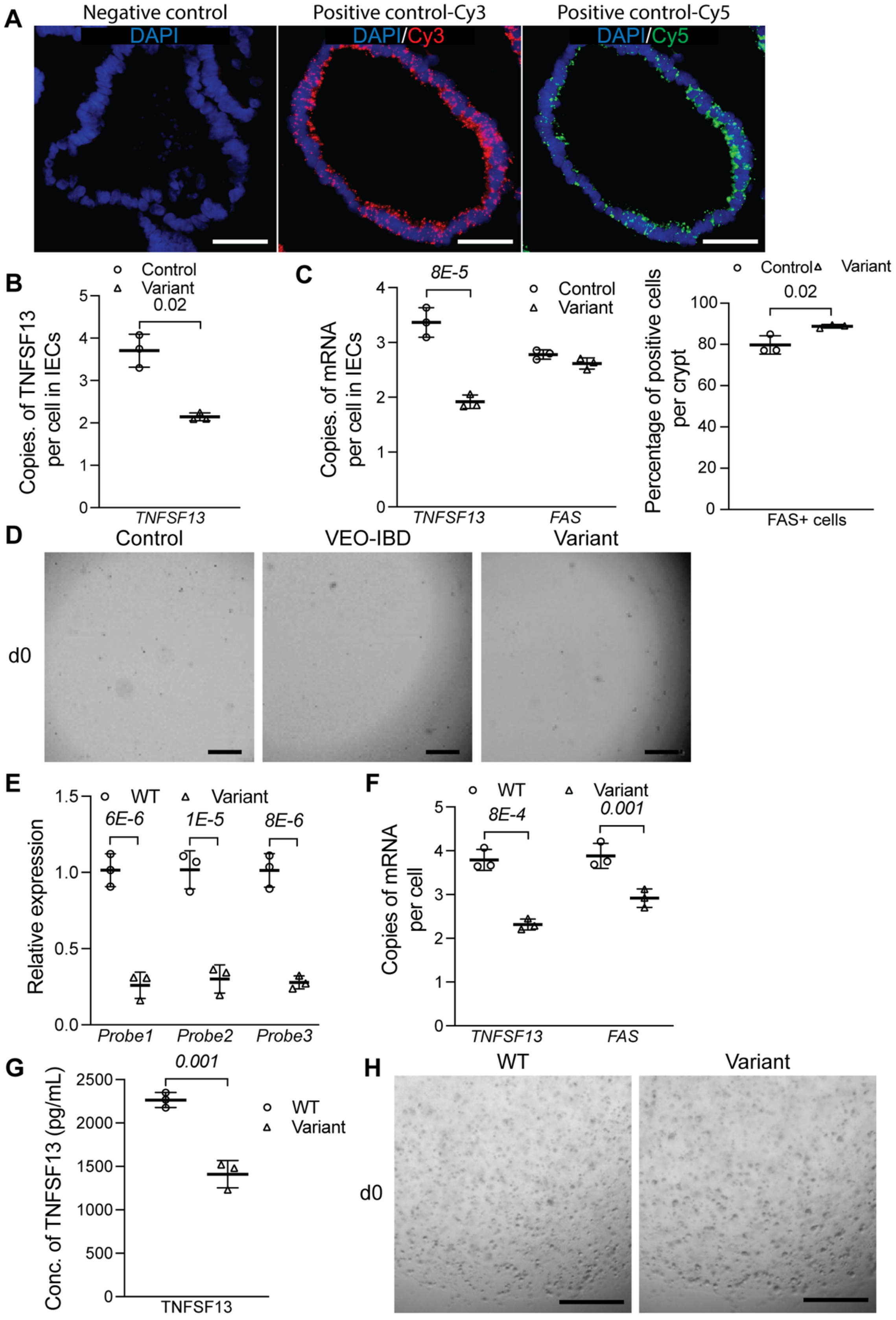

692

Supplementary Figure 2 related to Figure 1. Expression of TNFSF13 in human clonoids and iPSC-derived organoids. (A) Representative IF images for negative control and positive control RNAscope probes for *TNFSF13* (Cy3) and *FAS* (Cy5) probes in human colonoids in main figure. Scale bar: 50 μm. **(B)** Quantification of copies of *TNFSF13* (red dot) in colonoids in Figure 1B. n=3 lines of colonoids from 3 independent patients for control, n=3 passages of colonoids for variant. **(C)** Quantification of copies of *TNFSF13* (red dot) and *FAS* (green dot) (Left) and percentage of FAS$^+$ and FAS$^+$KI67$^+$ cells per crypt (Right) in IECs in Figure 1C. n=3 independent patients for control and VEO-IBD, n=3 slides from different blocks for variant**. (D)** Representative images of indicated samples for colonoid formation assays on d0 post-seeding, corresponding with Figure 1E. Scale bar: 300 μm. n=4 lines of colonoids from 4 different patients for control and VEO-IBD, n=4 independent passages of colonoids for variant. **(E)** qPCR for *TNFSF13* with different probes from different location in *TNFSF13* mRNA in WT and variant iPSC-organoids. n=3 passages of iPSC-organoids. **(F)** Quantification of *TNFSF13* (red dot) in organoids in Figure 1F. n=3 passages of iPSC-organoids. **(G)** ELISA for secreted TNFSF13 in iPSC-derived organoids culture conditioned media. n=3 passages of iPSC-organoids. **(H)** Representative images for organoid formation assay on d0 post seeding in WT and variant iPSC-derived organoids. Scale bar: 400 μm. n=3 passages of iPSC-organoids. Each passage has at least 2 technical replicates. *P* value as shown in the bar graphs unless *P*>0.05. Two-way ANOVA (with multiple comparisons) or two-tailed Student's *t*-test was used for statistical analysis in (B-C) and (E-G).

SUPPLEMENTARY FIGURE 3

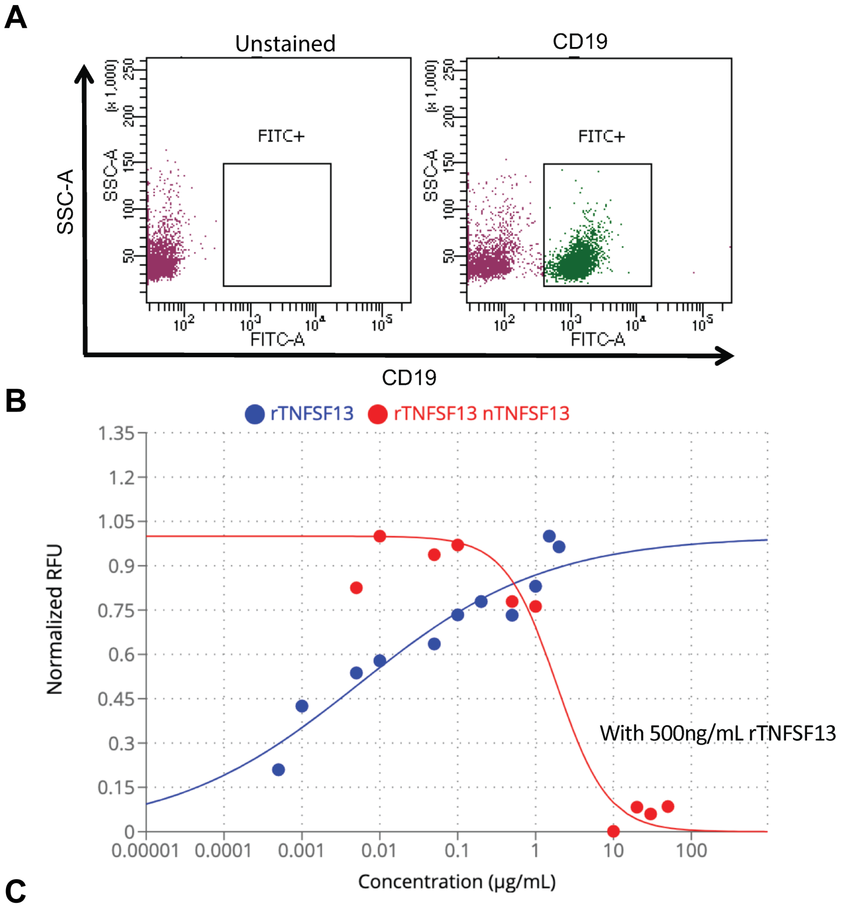

C

X_{50} Regression Results [rTNFSF13]

Parameter	Value
X_{50}	0.0054
Equations	
Equation	$Y = 0 + \frac{1 - 0}{1 + (\frac{X}{0.0054})^{-0.3617}}$
Equation Form	$Y = Min + \frac{Max - Min}{1 + (\frac{X}{X50})^{Hill\ coefficient}}$

X_{50} Regression Results [rTNFSF13 nTNFSF13]

Parameter	Value
X_{50}	1.8537
Equations	
Equation	$Y = 0 + \frac{1 - 0}{1 + (\frac{X}{1.8537})^{1.3152}}$
Equation Form	$Y = Min + \frac{Max - Min}{1 + (\frac{X}{X50})^{Hill\ coefficient}}$

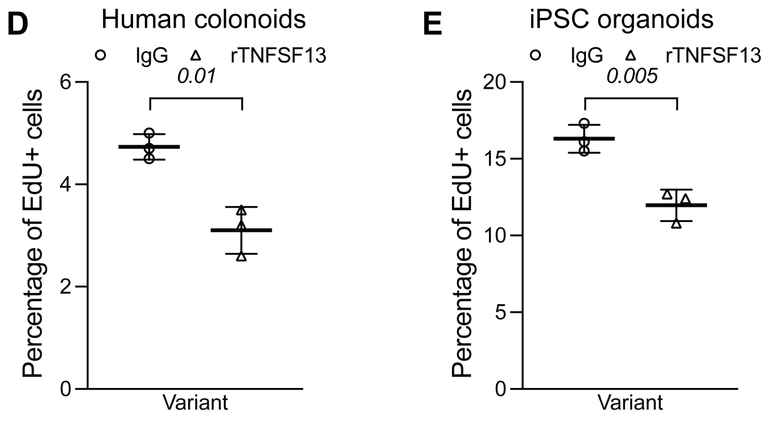

Supplementary Figure 3 related to Figure 1. TNFSF13 promotes proliferation of mouse splenic B cells with dose-dependent pattern. (A) FACS for sorting of mouse splenic DAPI$^-$CD19$^+$ B cells from n=3 WT mice. **(B)** Curve chart for cell proliferation assay with resazurin in mouse splenic B cells after treatment with rTNFSF13 and/or nTNFSF13. Red line: cells were treated with gradient concentration of rTNFSF13. Red line: cells were treated with gradient concentration of nTNFSF13 and 500 ng/mL rTNFSF13. n=3. **(C)** Equation for the curve in **(B)**. Left: equation for rTNFSF13 treatment curve (blue line). Right: equation for rTNFSF13+nTNFSF13 treatment curve (red line). (**D**) Percentage of EdU$^+$ cells after IgG or recombinant TNFSF13 (rTNFSF13) treatment for variant tissue-derived colonoids. (n=3 lines of colonoids from 3 different patients) or (**E**) variant iPSC-organoids at d7 post-seeding. n=3 passages of organoids. *P* values are shown on bar graphs unless P>0.05. Two-tailed Student t-test was used in (**D**-**E**).

SUPPLEMENTARY FIGURE 4

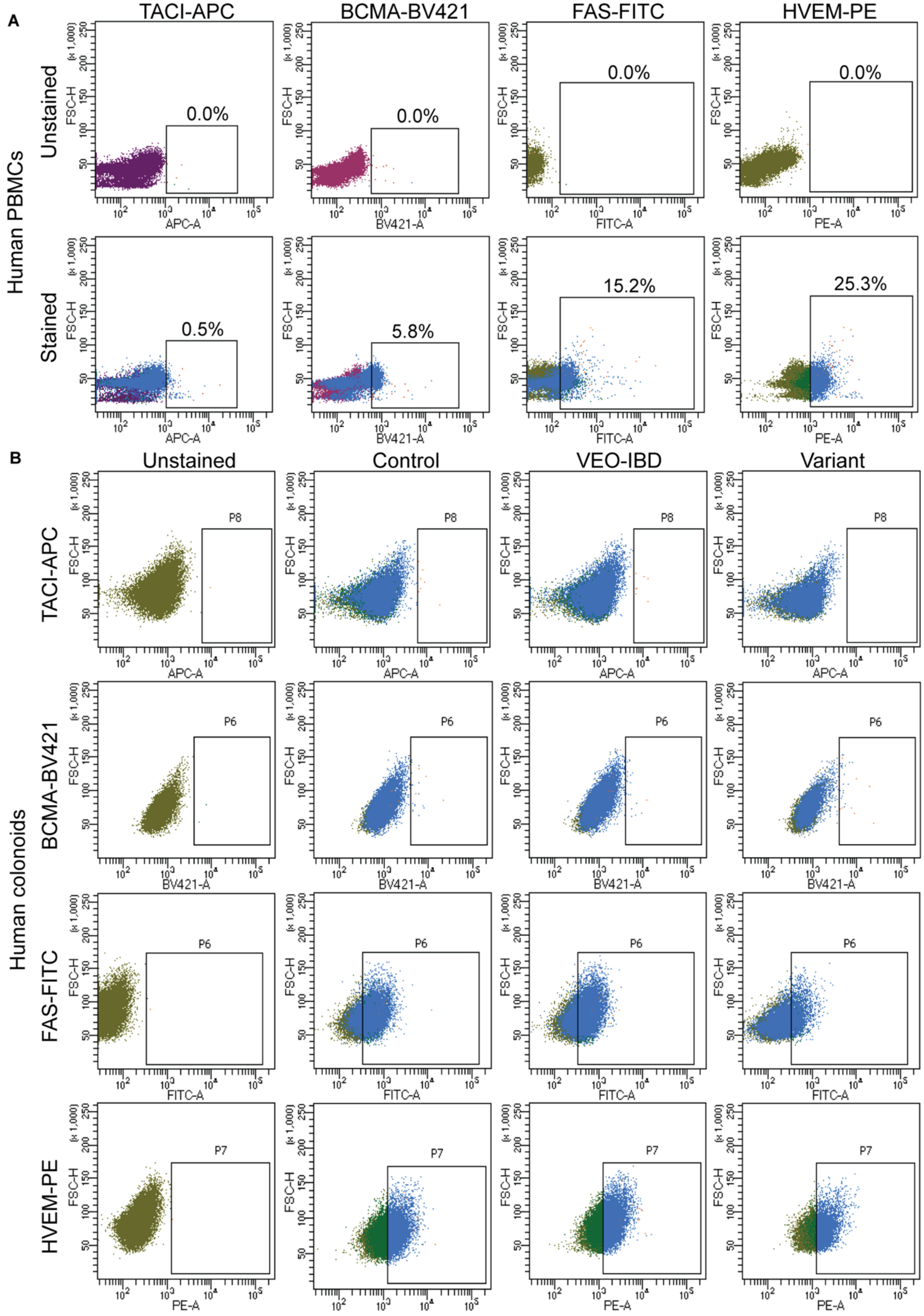

731

Supplementary Figure 4 related to Figure 2. FACS strategy for TNFSF13 receptors in human PBMCs and colonoids. (A) Verification of FACS antibodies of TNFSF13 in human PBMCs. Representative FACS images and percentage of population for $TACI^{+}$, $BCMA^{+}$, FAS^{+} and $HVEM^{+}$ in $DAPI^{-}$ population. **(B)** Representative FACS images for $TACI^{+}$, $BCMA^{+}$, FAS^{+} and $HVEM^{+}$ in $DAPI^{-}$ population in human colonoids of control, VEO-IBD and variant. The experiments were performed on independent patient lines as described in Figure 2 A & B.

SUPPLEMENTARY FIGURE 5

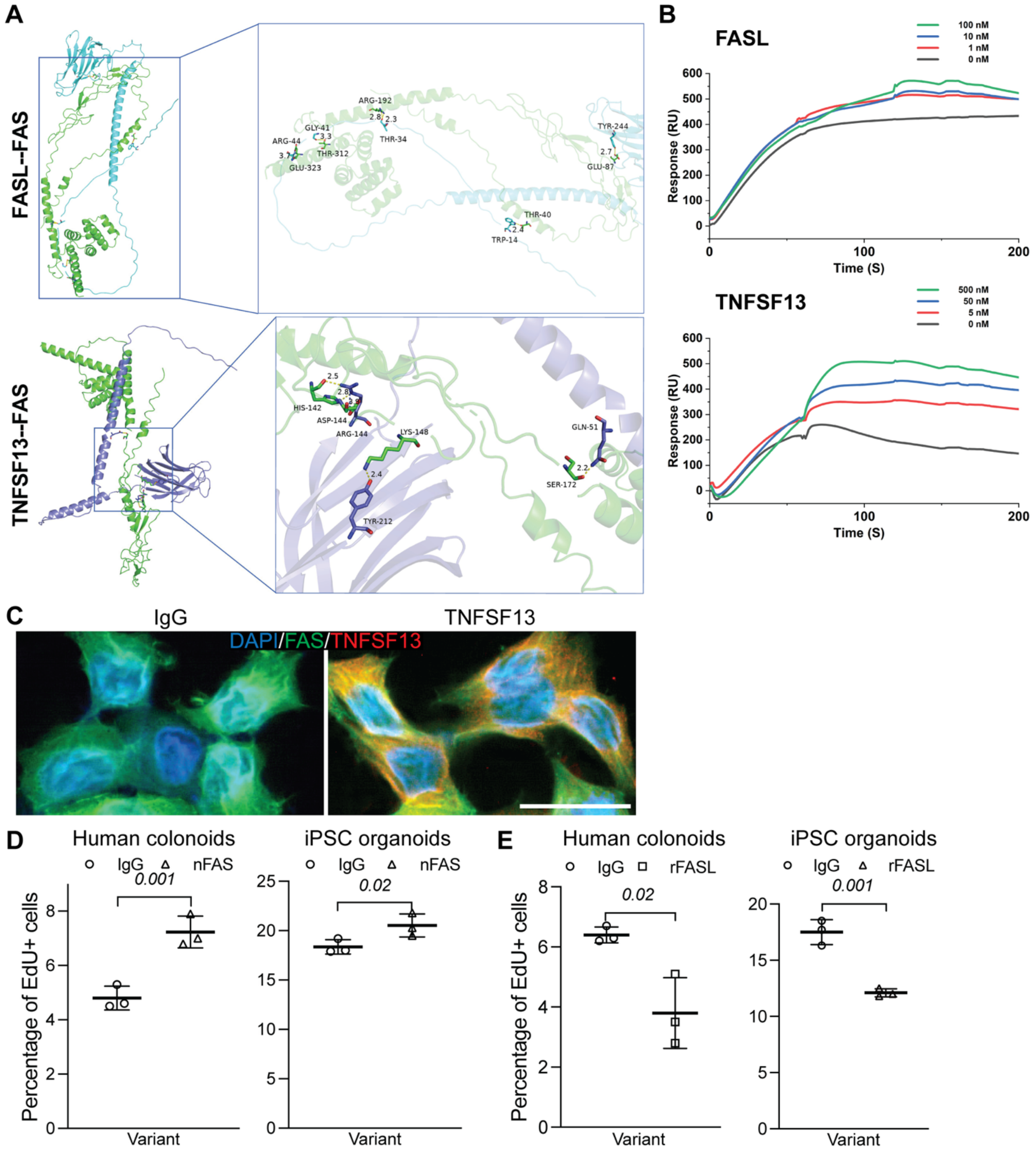

Supplementary Figure 5 related to Figure 2. TNFSF13-FAS interaction and EdU assay for nFAS and rFASL in variant organoids. (**A**) 3D structural models of docking analyses of FASL-FAS (upper) and TNFSF13-FAS (lower). FAS (green), FASL (blue),

and TNFSF13 (purple) are shown as ribbon structures; interacting residues are shown as sticks, with hydrogen bonds (yellow dashed lines) and salt bridges (blue dashed lines). (B) SPR competitive binding analyses of competing of FASL to TNFSF13-FAS (upper) and competing of TNFSF13 to FASL-FAS (lower) with different concentration. N=3 independent repeat. (**C**) Representative IF images for TNFSF13 and FAS-EGFP in FAS-expressing HEK293T cells. Scale bar: 50 μm. N=3 independent replicates/wells. (**D**) Percentage of EdU$^+$ cells in IgG or FAS neutralizing antibody (nFAS)-treated variant colonoids (left) or variant iPSC-organoids (right) on d7 post-seeding. (**E**) Percentage of EdU$^+$ cells in IgG or recombinant FAS ligand (rFASL)-treated variant colonoids (left) or variant iPSC-organoids (right) on d7 post-seeding. *P* values are shown on bar graphs unless $P>0.05$. Two-tailed Student t-test was used in (**D** and **E**). n= 3 lines of colonoids from 3 different patients for control and VEO-IBD, n=3 passages of colonoids for variant; n=3 passages each of iPSC lines.

SUPPLEMENTARY FIGURE 6

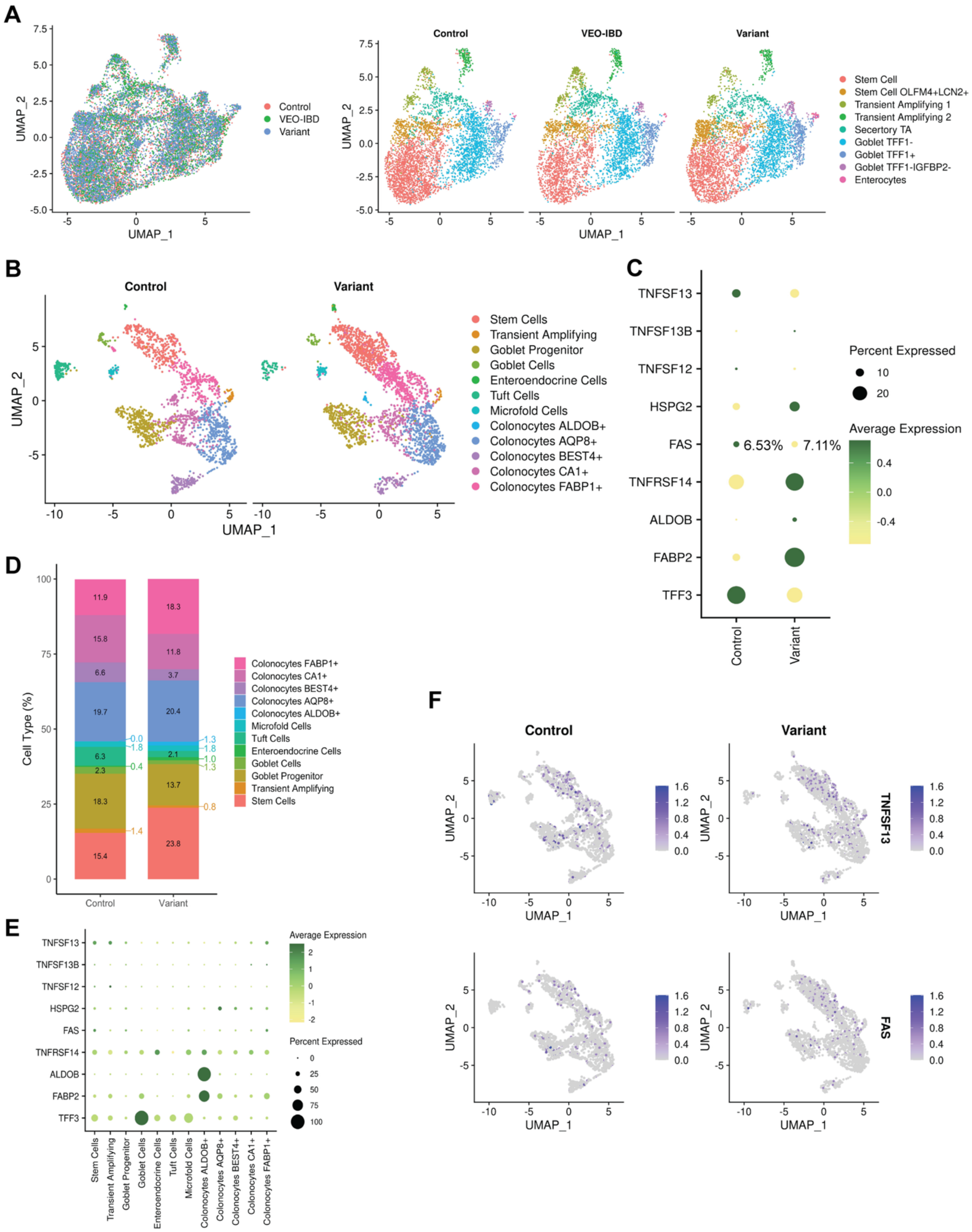

Supplementary Figure 6 related to Figure 3. scRNAseq analysis of human colonoids and biopsy. (A) UMAP visualizations of scRNA-seq data for human colonoids. n=2 lines of colonoids from 2 different patients for control and VEO-IBD, n=2 passages of colonoids for variant. Left: Overlay of the control, VEO-IBD and variant samples; Right: Annotated cell clusters of control, VEO-IBD and variant samples. **(B)** UMAP visualizations of scRNA-seq data for epithelial cells from healthy control and variant colon biopsies. **(C)** Dot plot with relative expression of selected genes of TNFSF13 family and related receptors and enterocyte markers among control and variant in human colon biopsies. Numeric in FAS category mean percentage of FAS^+ cells in annotated cells between control (6.53%) and variant (7.11%) group. **(D)** Barplot indicated relative proportion (%) of epithelial cells in 1 of control and 1 of variant in **(B)**. **(E)** Dot plot indicated the expression pattern of selected genes of TNFSF13 family and related receptors and enterocyte markers among annotated clusters for human biopsy scRNA-seq data with relative expression. **(F)** UMAP plots showing the expression pattern of TNFSF13 and FAS in annotated epithelial cells clusters for human biopsy scRNA-seq data. n=1 patient for control and variant.

SUPPLEMENTARY FIGURE 7

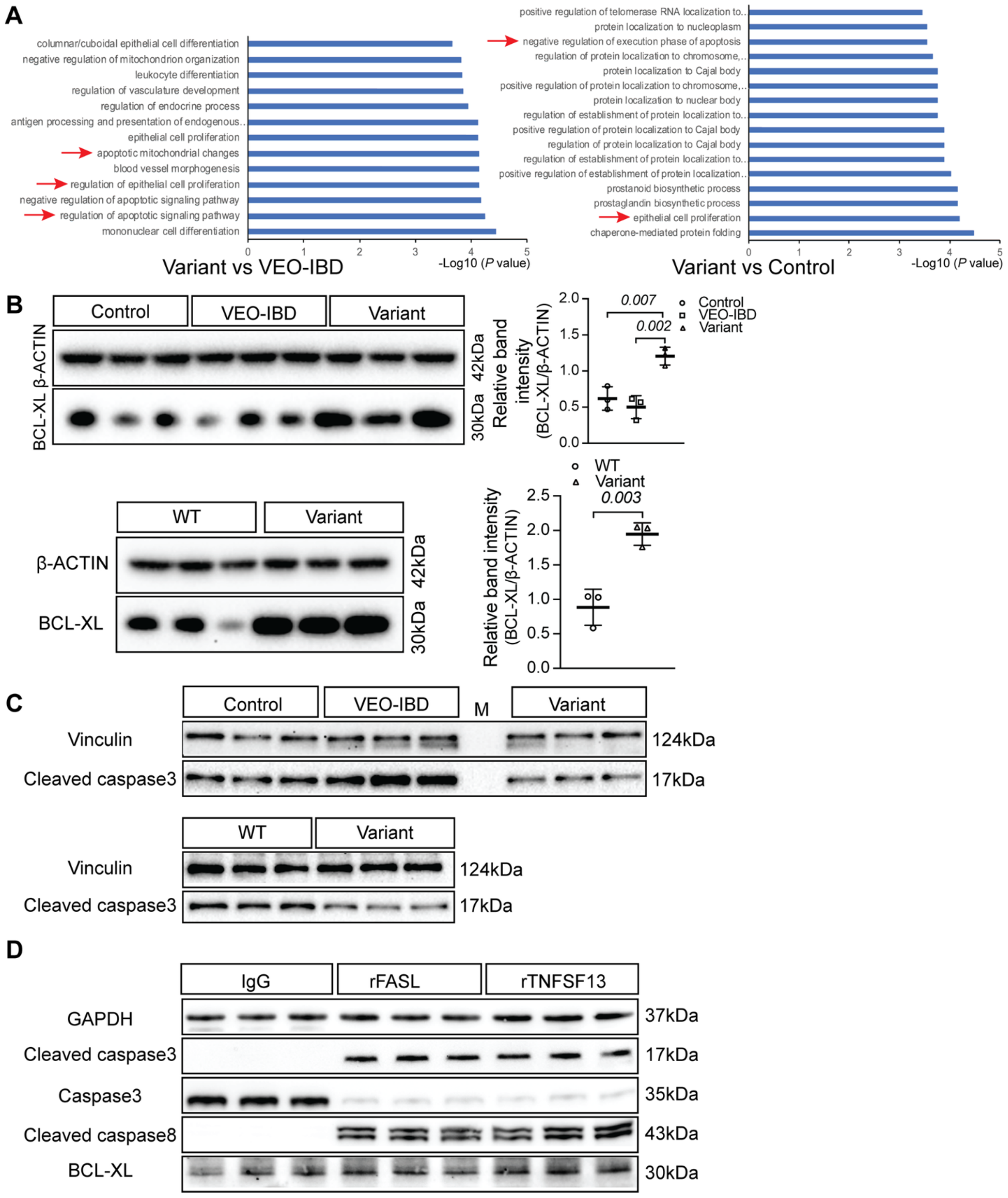

Supplementary Figure 7 related to Figure 4. GO analysis of biological process for human colonoids scRNAseq data. (A) GO analysis of biological process on DEGs in

human colonoids from scRNA-seq data. Bar graphs showing significantly changed biological processes between variant vs VEO-IBD, variant vs control (VEO-IBD vs control has no significantly changed category). Red arrow heads denote apoptosis and proliferation related categories. **(B)** Upper: representative western blot image (left) and quantification (right) for BCL-XL (with β -ACTIN as a loading control) in human colonoids. Lower: representative western blot image (left) and quantification (right) for BCL-XL in iPSC colonoids (with β-ACTIN as a loading control). (**C**) Representative western blot image for cleaved caspase3 (with Vinculin as a loading control) in human colonoids (Upper) and iPSC colonoids (Lower). 'M' denotes the protein ladder lane which was not overlayed in presented image. (**D**) Representative western blot image for cleaved caspase3, full length caspase3, cleaved caspase8 and BCL-XL (with GAPDH as a loading control) in IgG, rTNFSF13 and rFASL treated Jurkat T cells. N=3 independent experimental replicates are shown. *P* value shown in the bar graphs unless *P*>0.05. One-way ANOVA (with multiple comparisons) was used for statistical analysis. n=3 lines of colonoids from 3 different patients for control and VEO-IBD, n=3 passages of colonoids for variant. n=3 passages of iPSC-organoids. Three independent experimental replicates from 3 independent passages of WT and variant organoids lines are shown.

SUPPLEMENTARY FIGURE 8

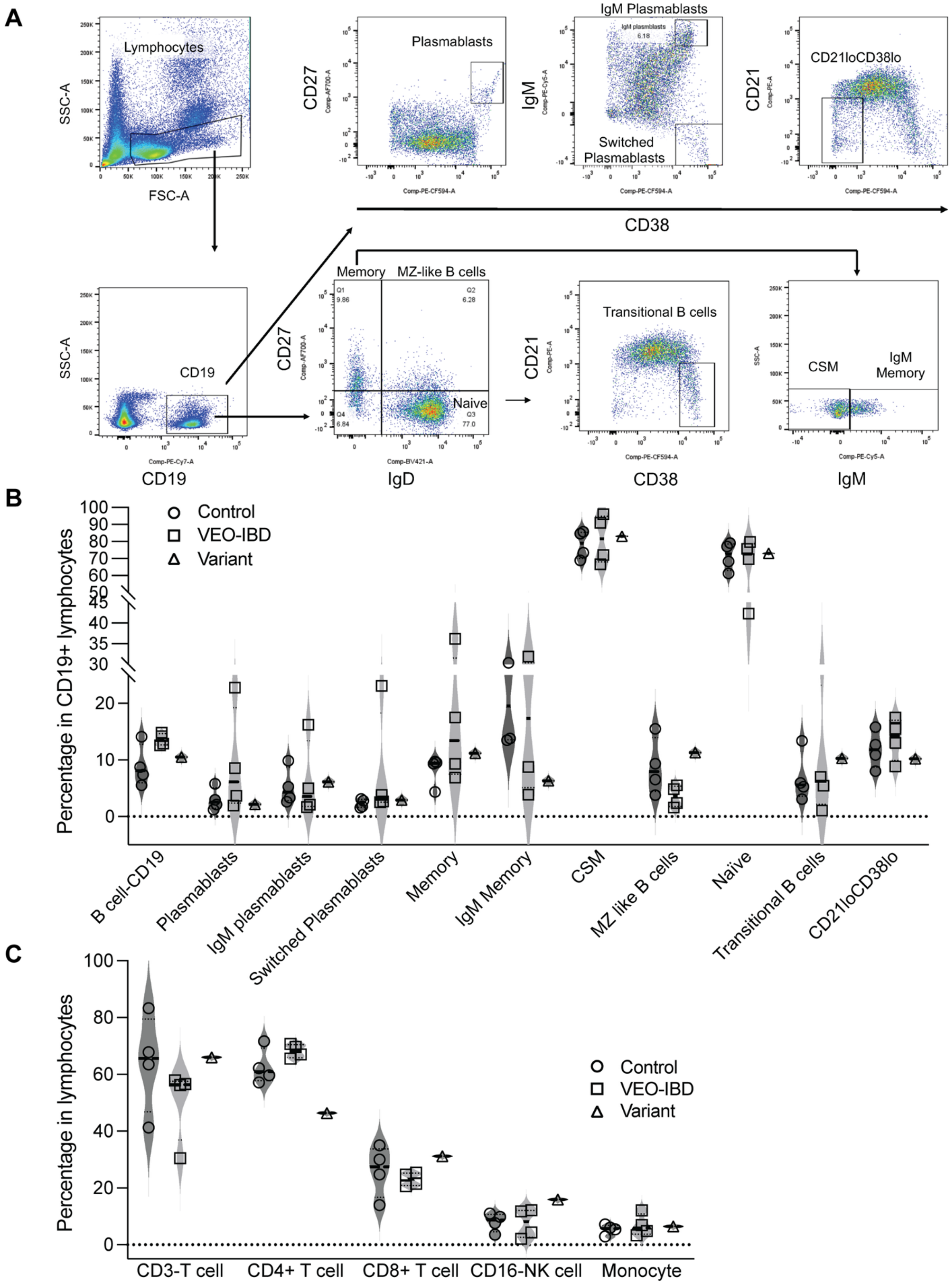

802

Supplementary Figure 8 related to Figure 5. No significant differences in immunophenotyping in PBMCs between variant and non-monogenic VEO-IBD subjects. (A) FACS strategy for identifying subtypes of B cells among human PBMCs from the corresponding patients. **(B)** Percentage of subtypes of B cells in DAPI^{-}CD19^{+} lymphocytes with FACS in 3 of control, 3 of VEO-IBD and 1 of variant patients. CSM: class-switched memory B cell; MZ: marginal zone. **(C)** Percentage of subtypes of other immune cells (T cells, Nature killer cells, Monocytes) in DAPI^{-} lymphocytes with FACS in 3 of control, 3 of VEO-IBD and 1 of variant patients. NK cell: Nature killer cells. n=4 different patients for Control and VEO-IBD, n=1 patient for Variant. Two-way ANOVA was used for statistical analysis only between Control and VEO-IBD. *P* value was not shown if $P>0.05$.

SUPPLEMENTARY FIGURE 9

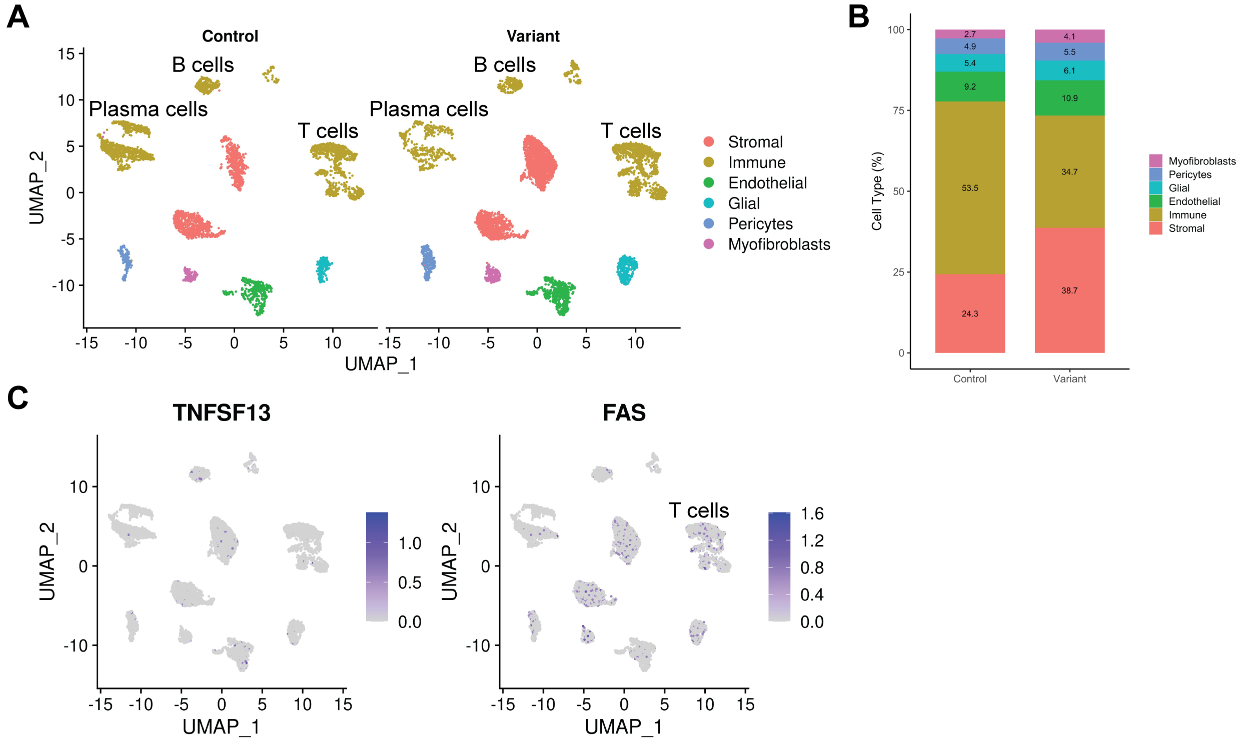

Supplementary Figure 9 related to Figure 5. scRNAseq analysis of human biopsies. (A) UMAP visualizations of scRNA-seq data for lamina propria cells from control and variant colon biopsies. **(B)** Barplot indicating cell type abundance (%) of lamina propria cells in 1 control and 1 variant. **(C)** UMAP plots showing the expression pattern of *TNFSF13* and *FAS* in annotated lamina propria cells clusters for human biopsy scRNA-seq data. n=1 patient for control and variant.

SUPPLEMENTARY FIGURE 10

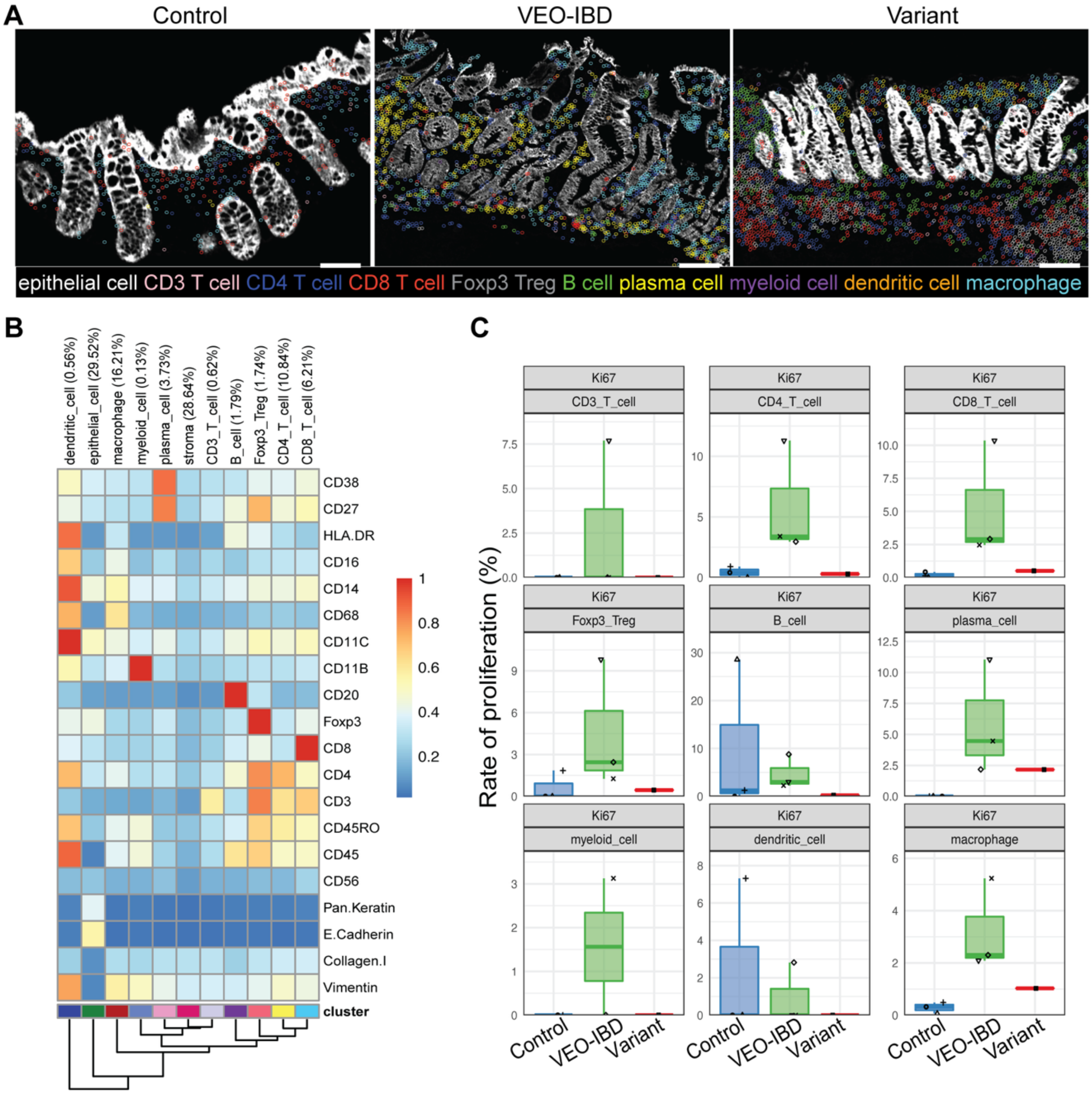

Supplementary Figure 10 related to Figure 5. Local immune analysis in colon by IMC. (A) Representative IMC overlay images for epithelial and immune cell markers in colon from 3 of control, 3 of VEO-IBD and 2 slides from different affected region of 1 variant patient. Scale bar: 100 μm. **(B)** Heatmap representing the expression profiles of the 11 annotated cell populations. Color scale represents the average expression of a given marker in each cell population. **(C)** Boxplot showing the rate of proliferation of

immune cell composition quantified by calculating the proportion of specific markers in cells that are $KI67^+$ in all cells at the same region (stroma + epithelium cell populations). Each point represents a sample/patient. n=3 different patients for control and VEO-IBD, n=3 slides from different blocks for variant.

SUPPLEMENTARY FIGURE 11

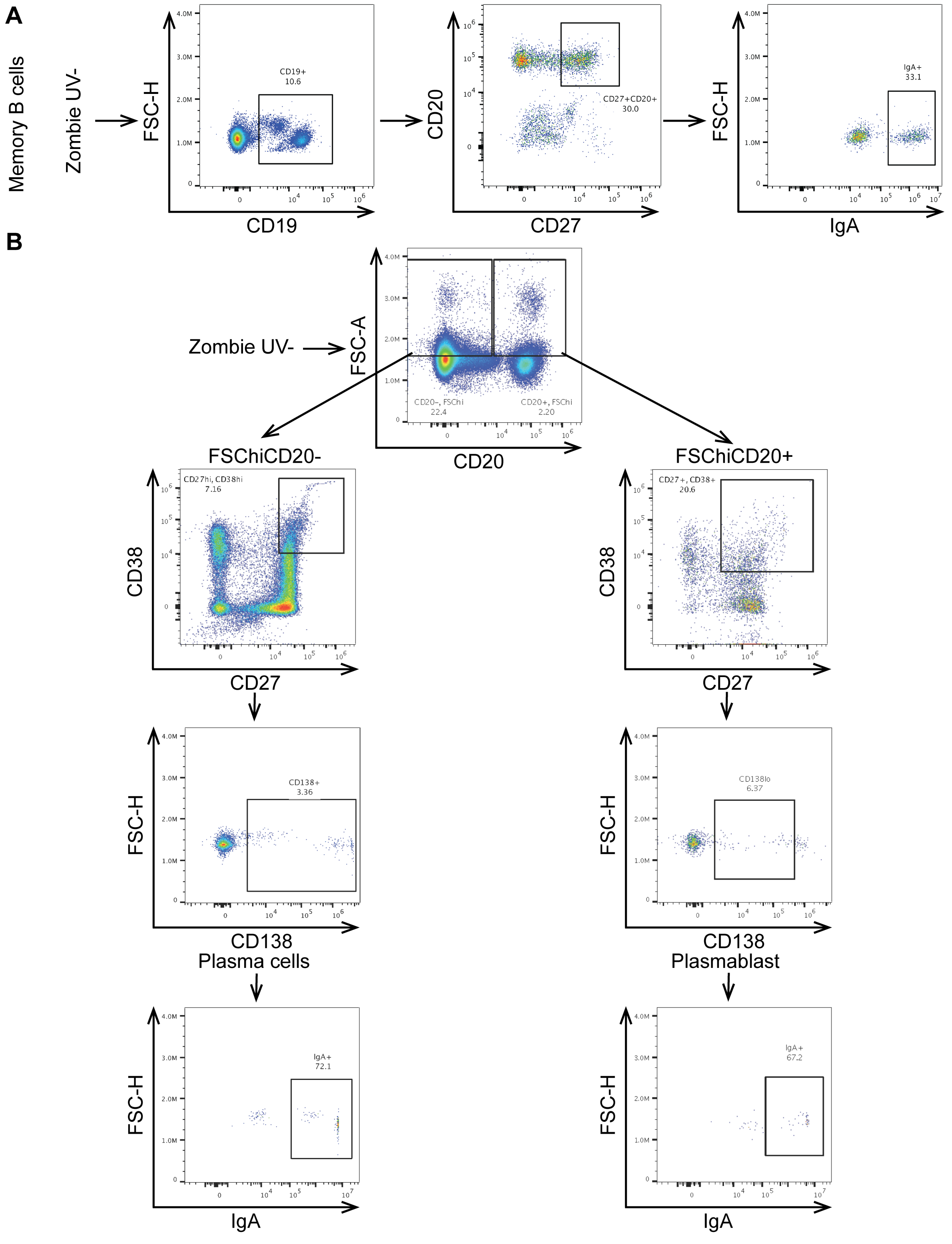

Supplementary Figure 11 related to Figure 6. FACS gating strategy for identifying human memory B cells, plasmablast, plasma cells and IgA$^+$ cells**. (A)** FACS gating strategy for identifying memory B cells (Zombie UV$^-$CD19$^+$CD27$^+$CD20$^+$) and IgA$^+$ memory B cells (Zombie UV$^-$CD19$^+$CD27$^+$CD20$^+$IgA$^+$) among human PBMCs. All cells were sorted from Zombie UV$^-$ population. **(B)** FACS gating strategy for identifying plasmablast (Zombie UV$^-$FSC^{hi}CD20$^+$CD27$^+$CD38$^+$CD138^{lo}) at d8-post seeding differentiated from sorted human memory B cells and plasma cells (Zombie UV$^-$FSC^{hi}CD20$^-$CD27^{hi}CD38^{hi}CD138$^+$) and IgA$^+$ plasma cells (Zombie UV$^-$FSC^{hi}CD20$^-$CD27^{hi}CD38^{hi}CD138$^+$IgA$^+$) at d14-post seeding. All cells were sorted from Zombie UV$^-$ population from human PBMCs or differentiated memory B cells. All the experiments were completed for 4 independent replicates.

SUPPLEMENTARY FIGURE 12

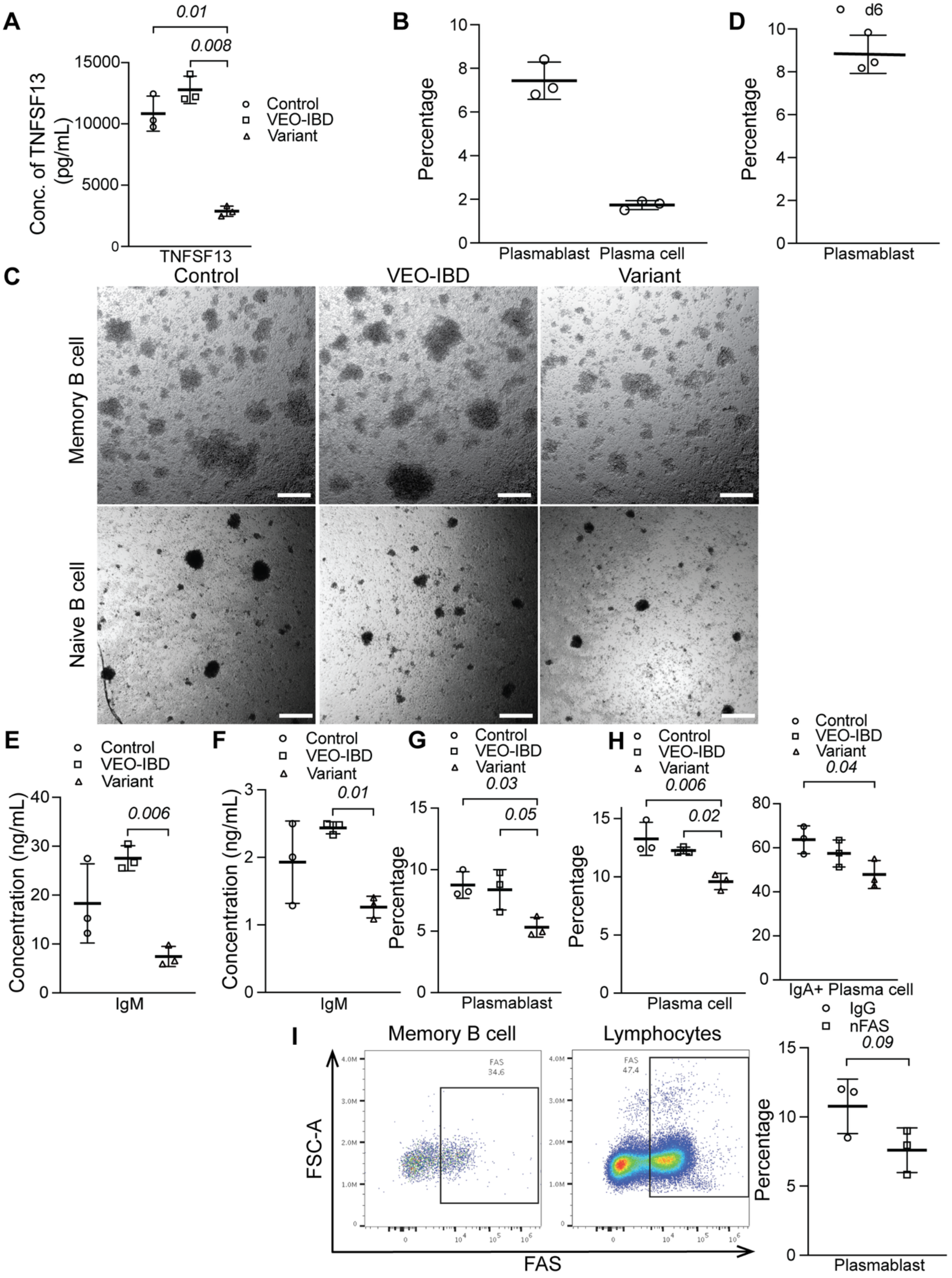

Supplementary Figure 12 related to Figure 6. TNFSF13 and differentiation of memory B cells. (A) ELISA for secreted TNFSF13 at d4-post seeding control, VEO-IBD and variant colonoids (3,000 clumps were seeded at d0) for co-culture and conditioned medium collection. The experiments were performed on independent patient lines as described in Figure 2 A & B. **(B)** Percentage of plasmablast differentiated from sorted human memory B cells at d8-post seeding and plasma cells differentiated from sorted human memory B cells at d14-post seeding by culturing in B cell medium. Three independent experiments from 3 donors were performed. **(C)** Representative images for differentiated memory B cell cluster (upper) and differentiated naïve B cell cluster (lower) at d8 post-seeding by culturing in mixture of B cell medium and conditioned medium. Clusters are growing cells. Scale bar: 300 µm (upper) and 500 µm (lower). **(D)** Percentage of plasmablasts differentiated from sorted human memory B cells at d6 post-seeding by culturing in B cell medium. Three independent experiments from 3 donors were performed. **(E)** ELISA for IgM in media from differentiated human memory B cells at day 14 post-seeding and **(F)** at day 14-post seeding by culturing in conditioned media mixture starting from day 6 post-seeding. The experiments were performed across 3 independent experiments with replicates. **(G)** Percentage of plasmablasts differentiated from sorted human naïve B cell at day 8 post-seeding via culturing in conditioned media consisting of B cell media and conditioned media (ratio 1:1). The experiment was performed across 3 independent replicates. **(H)** Percentage of plasma cells (left) and IgA^+ plasma cells (right) differentiated from sorted human naïve B cells at day 14 post-seeding with B cell media-conditioned media (ratio 1:1). The experiment was performed across 3 independent replicates. **(I)** Left: Representative FACS images for FAS^+ population in human memory

B cells and Lymphocytes from PBMCs. Right: Percentage of plasmablasts differentiated from IgG or FAS neutralizing antibody (nFAS)-treated human memory B cells at day 8 post-seeding with B cell media-conditioned media (ratio 1:1) from human control colonoids. The experiment was performed with three independent control colonoid lines. *P* value shown in the bar graphs unless $P>0.05$. One-way ANOVA (with multiple comparisons) or two-tailed Student's *t*-test was used for statistical analysis. n=3 lines of colonoids from 3 different patients for control and VEO-IBD, n=3 passages of colonoids for variant. n=13 independent donors to obtain human memory B cells.

SUPPLEMENTARY FIGURE 13

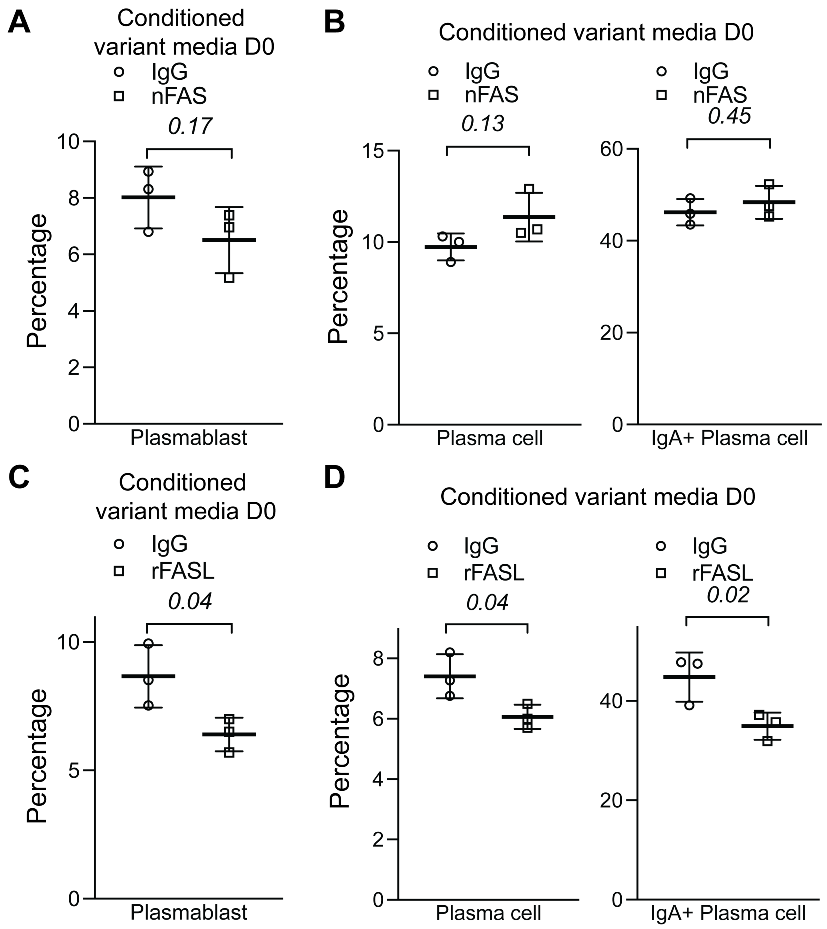

Supplementary Figure 13 related to Figure 6. TNFSF13 and differentiation of memory B cells with variant conditioned media. (**A**) Percentage of plasmablasts differentiated from IgG or FAS neutralizing antibody (nFAS)-treated human memory B cells at day 8 post-seeding with B cell media-conditioned media (ratio 1:1) from human variant colonoids. (**B**) Percentage of plasma cells (left) and IgA^+ plasma cells (right) differentiated from IgG or FAS neutralizing antibody (nFAS)-treated human memory B cells at day 14 post-seeding with B cell media-conditioned media (ratio 1:1) from human variant colonoids. (**C**) Percentage of plasmablasts differentiated from IgG or rFASL-treated human memory B cells at day 8 post-seeding with B cell media-conditioned media (ratio 1:1) from human variant colonoids. (**D**) Percentage of plasma cells (left) and IgA^+

plasma cells (right) differentiated from IgG or rFASL-treated human memory B cells at day 14 post-seeding with B cell media-conditioned media (ratio 1:1) from human variant colonoids. The experiment was performed with three independent variant colonoid lines with three different passages from the same variant patient. *P* value shown in the bar graphs. Two-tailed Student's *t*-test was used for statistical analysis. n=3 lines of colonoids from 3 different patients for control and VEO-IBD.

Supplementary Table 1 Patients demographics and speciement

Colonoid line	Age at diagnosis (y)	Age at collection(y)	Sex	Diagnosis	Disease activity at collection	Used for
TNFSF13 variant RC-A	0.58	7.44	M	VEO-IBD	moderate	WES, TOPO TA colone, qPCR, OFR, monolayer, Western blotting, flow cytometry, scRNA-seq (colonoids), IMC, ELISA, immunostaining
TNFSF13 variant RC-A	0.58	11.09	M	VEO-IBD	moderate	scRNA-seq (biopsy)
**IBD/VEO-IBD control**						
VEO-IBD-1	4.33	6.33	M	VEO-IBD	Severe	qPCR, OFR, monolayer, Western blotting, flow cytometry, scRNA-seq, IMC, ELISA, immunostaining
VEO-IBD-2	4.08	4.34	M	VEO-IBD	moderate	
VEO-IBD-3	5.5	5.5	F	VEO-IBD	Severe	
VEO-IBD-4	1.83	7.4	M	VEO-IBD	moderate	FACS with PBMCs for verification of TNFSF13 receptors
**Healthy control**						
Control-1	N/A	6	M	N/A	N/A	qPCR, OFR, monolayer, Western blotting, flow cytometry, scRNA-seq, IMC, ELISA, immunostaining
Control-2	N/A	3.78	F	N/A	N/A	
Control-3	N/A	5.45	F	N/A	N/A	
Control-4	N/A	3.97	M	N/A	N/A	flow cytometry (PBMCs)
Control-5	N/A	10.13	M	N/A	N/A	TOPO TA colone
Control-6	N/A	9.78	M	N/A	N/A	scRNA-seq (biopsy)

Supplementary Table 2 Antibodies and primers

Antibody/probe	Catalog number	Used for
Human APRIL/TNFSF13 Antibody	MAB5860	B cell and colonoids neutralizing
Recombinant Human APRIL/TNFSF13 (HEK293-expressed) Protein	5860-AP-010	B cell, colonoids, cell line, SPR
Mouse IgG1 Isotype Control	MAB002	B cell, colonoids, cell line, SPR
Recombinant ruman Fas ligand/TNFSF6 Protein	126-FL-010	colonoids, cell line, SPR
FAS	05-338	Neutralizing
Mouse IgG1 Negative Control	MABC002	Neutralizing control for FAS
Apc anti-human CD267 (TACI)	#311911	FACS
Brilliant Violet 421™ anti-human CD269 (BCMA)	#357519	FACS
FITC anti-CD95 Mouse Monoclonal Antibody	#305605	FACS
PE anti-HVEM (TR2) Mouse Monoclonal Antibody	#318805	FACS
FITC anti-mouse CD19	#115505	FACS
PE-Cy7 anti-human CD326 (EpCAM) Antibody	#324222	FACS
FITC anti-human CD326 (EpCAM) Antibody	#324203	FACS
IgA Antibody, anti-human, APC	#130-113-998	FACS
Anti-CD27 Mouse Monoclonal Antibody (FITC) [clone: M-T271]	#356403	FACS
BD Pharmingen™ APC Mouse Anti-Human CD38	#560980	FACS
Anti-CD19 Mouse Monoclonal Antibody (PE) [clone: SJ25C1]	#363003	FACS
Anti-CD138 Mouse Monoclonal Antibody (PE) [clone: MI15]	#356503	FACS
Anti-CD20 Mouse Monoclonal Antibody (PE/Cy7®) [clone: 2H7]	#302311	FACS
Anti-CD138 Mouse Monoclonal Antibody (Brilliant Violet® 605) [clone: MI15]	#356519	FACS
BD Pharmingen™ APC-H7 Mouse Anti-Human CD20	#560734	FACS
Alexa Fluor® 700 anti-human CD19 Antibody	#302226	FACS
BD Pharmingen™ APC Mouse Anti-Human IgG	#550931	FACS
Goat F(ab')2 Anti-Human IgA-PE; 0.25 mg	#2052-09	FACS
F(ab')2-Goat anti-Mouse IgM (mu) Antibody	16-5092-85	mouse B cell stimulating
anti-Ki67 antibody	ab16667	immunostaining
anti-E-Cadherin antibody	#610182	immunostaining
anti-FABP2/I-FABP antibody	AF3078-SP	immunostaining
TNFSF13 probe	#406981-C2	RNAscope
FAS probe	# 427031	RNAscope
positive control probe	#320881	RNAscope
negative control probe	#320871	RNAscope
Human TNFSF13 Antibody	MAB8844	co-IP capture antibody
Mouse IgG2B Isotype Control	MAB004	co-IP
Recombinant Anti-Fas antibody	ab133619	Western blotting
BCL-XL Antibody	CST#2762S	Western blotting
Monoclonal Anti-β-ACTIN antibody	A5316-.2ML	Western blotting
specific fluorophore-conjugated secondary antibodies AffiniPure IgG	Jackson ImmunoResearch	immunostaining
Opal™ 690 fluorophore	#FP1497001KT	RNAscope
Opal™ 570 fluorophore	#FP1488001KT	RNAscope
Peroxidase (HRP) Anti-Rabbit IgG Goat Secondary Antibody	CST#7074S	Western blotting
Rabbit Anti-Mouse IgG H&L (HRP) (ab6728)	ab6728	Western blotting
Rabbit anti-Mouse IgG (H+L) Secondary Antibody [HRP]	NBP1-75249	Western blotting
Anti-mouse IgG VeriBlot for IP secondary antibody	ab131368	Western blotting
Caspase-3 Antibody	CST#9662	Western blotting
Vinculin Antibody	CST#4650	Western blotting
Cleaved Caspase-3 (Asp175) Antibody	CST#9661	Western blotting
Cleaved Caspase-8 (Asp387) (D5B2) Rabbit Monoclonal Antibody	CST#8592	Western blotting
GAPDH Loading Control Monoclonal Antibody (GA1R)	#MA5-15738	Western blotting
BD Horizon™ BV711 Mouse Anti-Human CD45	#564357	FACS
EasySep™ Human Memory B Cell Isolation Kit	#17864	memory B cell isolating

**qPCR-Taqman**		
TNFSF13	Hs00601664_g1	
TNFSF13B	Hs00198106_m1	
TNFSF12-TNFSF13	Hs01650719_m1	

**qPCR-SYBR Green**	5' to 3'	
	**Forward**	**Reverse**
TNFSF13 qF1	GGCAACCAGCTCTTAGGCG	AAGTCACGTCTTGAAACAGGAC
TNFSF13 qF2	TGCCCTCTGGTTGAGTTGG	CCATTCTCCCAGGCTTCCAG
TNFSF13 qF3	GGGTCAGGTGGTGTCTCG	AAGTTTCGCCCTTGCCCG
BCL2L1	GAGCTGGTGGTTGACTTTCTC	TCCATCTCCGATTCAGTCCCT
ACAA2	AAGTCTCACCTGAAACAGTTGA	CACGCAAACCAACATGCCT
ID1	CTGCTCTACGACATGAACGG	GAAGGTCCCTGATGTAGTCGAT
ECM1	AGCACCCCAATGAACAGAAGG	CTGCATTCCAGGACTCAGGTT

AldoB	TGTCTGGTGGCATGAGTGAAG	GGCCCGTCCATAAGAGAAACTT

**TOPO TA colone**	5' to 3'	
	**Forward**	**Reverse**
TNFSF13 eF5	AGCGTGGGGATTGTAAGC	CTGGGGTTACCTGGCTAT

Supplementary Table 3 Effective cell count for scRNAseq

Human colonoids

Sample ID	Pre Filtering	Post Filtering
Control-1	2,915	2,487
Control-2	3,001	2,318
VEO-IBD-2	2,811	2,394
VEO-IBD-3	2,373	1,883
Variant-1	3,159	2,442
Variant-2	2,927	2,240
in total	17,186	13,764

Human biopsy

	Pre-QC	Post-QC	Final-QC*
Variant-LPL	8226	7175	6014
Control-6-LPL	8743	6434	4755
Variant-epithelial	6101	4302	2814
Control-6-epithelial	6409	3307	2207

*Final-QC: after removal of clusters based on top markers (doublets and markers limited to mitochondrial or ribosomal genes)

Supplementary Table 4 DEGs for scRNAseq in human colonoids

Variant vs VEO-IBD

Genes	log2 FC	Pct Variant	Pct VEO	Adj P Val
LCN2	1.272	0.862	0.728	1.21E-170
PRSS2	1.026	0.405	0.159	2.74E-158
HES1	0.826	0.823	0.662	5.57E-223
TFF3	0.768	0.876	0.740	4.58E-116
BTG2	0.702	0.752	0.514	8.82E-204
VMP1	0.698	0.894	0.734	2.28E-262
ATF3	0.688	0.678	0.419	3.09E-200
CD24	0.684	0.936	0.774	0.00E+00
NOTUM	0.676	0.317	0.080	1.31E-175
KLF6	0.648	0.804	0.618	4.66E-184
PRAC1	0.625	0.592	0.176	0.00E+00
MMP7	0.620	0.308	0.161	3.19E-64
FUT9	0.609	0.588	0.355	3.75E-152
SERPINA1	0.562	0.383	0.175	1.10E-112
S100A4	0.546	0.368	0.238	6.01E-50
ECM1	0.541	0.423	0.257	1.72E-75
IL32	0.526	0.385	0.136	9.43E-164
DPYSL2	0.522	0.806	0.660	3.95E-135
RHOB	0.501	0.715	0.489	2.82E-157
MUC1	0.501	0.531	0.391	1.32E-59
ODAM	0.499	0.335	0.114	1.16E-141
ITGB8	0.431	0.493	0.267	1.13E-125
ZFP36L1	0.421	0.795	0.646	2.25E-105
PROX1	0.418	0.405	0.229	1.48E-81
IVNS1ABP	0.418	0.728	0.571	4.36E-97
ACAA2	0.416	0.471	0.352	5.30E-50
SLC38A11	0.410	0.271	0.072	1.03E-135
ELF3	0.409	0.781	0.634	1.67E-89
CDK6	0.409	0.753	0.623	5.28E-88
DST	0.402	0.576	0.386	4.33E-91
NFAT5	0.401	0.587	0.436	6.85E-76
NKD1	0.394	0.491	0.336	1.76E-66
EGR1	0.393	0.598	0.445	1.04E-71
IFI6	0.392	0.346	0.150	6.83E-108
MME	0.391	0.274	0.088	3.96E-114
ID1	0.390	0.409	0.243	4.50E-73
RPS4Y1	0.386	0.946	0.546	1.73E-128
RARRES2	0.385	0.425	0.273	2.36E-60
PLK2	0.377	0.588	0.425	3.54E-71
TUBA1A	0.376	0.427	0.296	1.34E-46
IRF2BP2	0.367	0.834	0.691	1.62E-99
CTNNB1	0.365	0.812	0.684	7.48E-89
KDM5B	0.358	0.633	0.452	1.01E-83
DUSP1	0.352	0.614	0.462	3.44E-67
MSI2	0.350	0.722	0.569	1.76E-82
ABHD2	0.347	0.557	0.383	5.25E-78
CLDN4	0.344	0.830	0.697	7.73E-84
SEMA6A	0.342	0.329	0.117	1.79E-126
DEPP1	0.334	0.281	0.101	5.45E-102
PBX1	0.318	0.419	0.228	4.33E-87
TFDP2	0.309	0.504	0.353	8.54E-61
TRBC2	0.309	0.347	0.179	2.02E-75
KLF5	0.306	0.795	0.685	3.25E-60
TUBB2B	0.305	0.242	0.079	4.10E-94
TCF4	0.303	0.427	0.270	1.31E-57
ACTN1	0.302	0.769	0.655	2.76E-63
NFIA	0.301	0.695	0.573	3.22E-55
RUNX1	0.296	0.518	0.368	4.83E-58
CDKN1C	0.293	0.338	0.232	5.63E-29
SNHG14	0.288	0.499	0.373	1.29E-41
TRIB1	0.288	0.589	0.458	6.75E-50
GTF2I	0.287	0.763	0.662	3.98E-53
DUSP4	0.285	0.428	0.285	2.00E-52
AL354707.1	0.283	0.277	0.090	7.13E-112
LPP	0.282	0.654	0.523	4.85E-52
APCDD1	0.278	0.404	0.292	2.11E-30
ZMYND8	0.277	0.599	0.460	2.02E-51
INHBB	0.277	0.267	0.126	2.98E-62
ZBTB20	0.275	0.368	0.215	8.37E-59
ARHGAP5	0.275	0.660	0.525	4.31E-52
USP9Y	0.270	0.293	0.114	3.80E-94
SNHG5	0.267	0.716	0.575	1.84E-55
AC022075.1	0.266	0.273	0.166	9.37E-35
PTK7	0.265	0.626	0.507	9.59E-43
TACSTD2	0.265	0.345	0.216	3.84E-42
NUDT4	0.264	0.499	0.371	4.02E-44
NET1	0.261	0.588	0.455	1.47E-46

Variant vs Control

Genes	log2 FC	Pct Variant	Pct Control	Adj P Val
LCN2	1.914	0.862	0.512	0.00E+00
TFF3	1.142	0.876	0.677	1.00E-259
MMP7	0.833	0.308	0.075	1.26E-187
MUC1	0.773	0.531	0.253	4.82E-205
PRSS2	0.713	0.405	0.296	3.70E-40
SERPINA1	0.686	0.383	0.143	1.87E-171
KLK7	0.684	0.686	0.470	1.81E-166
NOTUM	0.622	0.317	0.116	1.33E-135
ECM1	0.563	0.423	0.258	3.59E-83
RPS4Y1	0.554	0.946	0.497	1.97E-238
TCN1	0.513	0.285	0.139	2.61E-72
RARRES2	0.466	0.425	0.256	3.79E-88
SLC6A8	0.459	0.195	0.095	3.85E-45
ODAM	0.412	0.335	0.166	1.19E-87
IL32	0.407	0.385	0.233	2.21E-69
ID1	0.371	0.409	0.255	1.06E-68
ABHD2	0.368	0.557	0.389	3.16E-87
TACSTD2	0.366	0.345	0.149	1.49E-108
PLK2	0.357	0.588	0.442	2.26E-67
IFI6	0.354	0.346	0.175	3.90E-87
TCF4	0.351	0.427	0.248	3.72E-82
BTG2	0.351	0.752	0.644	3.65E-59
ANXA1	0.347	0.487	0.347	4.64E-47
AC020656.1	0.346	0.504	0.398	2.70E-39
ITGB8	0.333	0.493	0.338	1.81E-70
IGFBP3	0.324	0.420	0.284	5.02E-41
ZFP36L1	0.323	0.795	0.693	8.73E-66
TRBC2	0.318	0.347	0.178	2.82E-84
AHNAK	0.304	0.634	0.528	2.57E-41
GABRA2	0.296	0.254	0.068	7.01E-131
RHOB	0.296	0.715	0.596	9.08E-58
CD74	0.290	0.443	0.294	6.87E-60
ATF3	0.287	0.678	0.558	1.80E-44
ADIRF	0.281	0.318	0.215	1.05E-28
BCL2L1	0.271	0.525	0.407	4.13E-42
USP9Y	0.271	0.293	0.117	3.60E-99
TSHZ2	0.270	0.299	0.125	1.68E-94
IFITM2	0.267	0.190	0.016	2.06E-169
GSN	0.266	0.654	0.526	7.15E-55
KCTD12	0.263	0.235	0.103	3.29E-66
DNAJC15	0.257	0.375	0.208	2.00E-72
AKR1C3	0.256	0.408	0.296	1.38E-36
PDLIM4	0.251	0.334	0.210	7.81E-45
MDH2	-0.250	0.511	0.638	4.43E-52
TXNL4A	-0.251	0.382	0.541	1.26E-60
SNRPD1	-0.252	0.510	0.635	1.14E-45
TCIM	-0.255	0.022	0.156	1.32E-110
PRDX6	-0.255	0.611	0.735	2.62E-56
TRAP1	-0.255	0.246	0.422	6.66E-75
EIF1AX	-0.256	0.643	0.767	3.25E-58
RGMB	-0.257	0.333	0.474	1.74E-44
CCT8	-0.257	0.555	0.697	2.45E-60
NASP	-0.260	0.568	0.670	3.80E-34
ANP32B	-0.262	0.609	0.737	3.90E-54
CFAP97	-0.262	0.371	0.536	6.52E-64
CYC1	-0.264	0.328	0.484	1.49E-60
PARP1	-0.264	0.513	0.671	1.88E-59
LSM4	-0.265	0.519	0.675	1.87E-61
TOMM5	-0.266	0.429	0.580	1.99E-56
MRPS34	-0.268	0.443	0.604	1.31E-62
ITPR2	-0.268	0.420	0.583	7.82E-55
CCT6A	-0.270	0.630	0.752	5.87E-56
GSTM3	-0.271	0.437	0.602	1.35E-64
TOMM40	-0.272	0.212	0.389	6.46E-80
FERMT1	-0.274	0.319	0.499	1.53E-74
AURKAIP1	-0.275	0.541	0.697	7.09E-70
IFI27L2	-0.276	0.165	0.357	1.48E-99
TRMT112	-0.276	0.517	0.670	5.21E-69
PRKDC	-0.276	0.623	0.754	6.03E-56
ASCL2	-0.279	0.652	0.766	1.29E-40
DNMT1	-0.279	0.245	0.398	1.75E-57
PAICS	-0.279	0.370	0.551	5.20E-75
SNHG17	-0.280	0.122	0.298	1.42E-98
EBPL	-0.281	0.314	0.498	9.34E-80
FBL	-0.282	0.413	0.589	1.14E-73
AZGP1	-0.283	0.232	0.411	3.14E-80
HMGA1	-0.283	0.676	0.798	8.25E-69

VEO-IBD vs Control

Genes	log2 FC	Pct VEO	Pct Control	Adj P Val
OLFM4	1.061	0.607	0.170	0.00E+00
IFI27	0.714	0.440	0.189	5.06E-180
PAX8-AS1	0.713	0.430	0.007	0.00E+00
LCN2	0.642	0.728	0.512	8.63E-113
AC020656.1	0.516	0.616	0.398	3.86E-130
HLA-C	0.516	0.932	0.827	2.93E-184
IGFBP3	0.506	0.503	0.284	9.36E-100
PIGR	0.505	0.376	0.172	2.07E-112
ANXA1	0.501	0.585	0.347	5.13E-120
ADIRF	0.498	0.466	0.215	5.40E-152
TCN1	0.417	0.299	0.139	1.13E-76
CDC42EP5	0.413	0.500	0.314	1.92E-92
UCA1	0.401	0.493	0.331	2.84E-62
PLP2	0.400	0.406	0.163	2.09E-154
KLK6	0.391	0.782	0.677	4.49E-71
KLK7	0.364	0.588	0.470	1.41E-45
SLCO1B3	0.331	0.430	0.245	2.81E-83
LGALS1	0.327	0.268	0.166	1.54E-31
SLC14A1	0.302	0.333	0.193	1.27E-47
MUC1	0.272	0.391	0.253	4.18E-45
HLA-E	0.266	0.618	0.515	7.27E-41
SEMA6A	-0.256	0.117	0.289	8.60E-88
PLCG2	-0.258	0.311	0.454	2.15E-45
PRSS23	-0.261	0.403	0.549	1.64E-51
EPHB2	-0.261	0.270	0.436	1.94E-62
SNHG17	-0.272	0.127	0.298	7.24E-87
PROX1	-0.274	0.229	0.386	2.25E-57
SNHG5	-0.279	0.575	0.736	1.13E-69
MSI2	-0.281	0.569	0.691	1.92E-50
GABPB1-AS1	-0.291	0.584	0.693	3.64E-47
GPR155	-0.292	0.083	0.259	3.38E-106
DACH1	-0.294	0.180	0.364	2.14E-85
MUC5B	-0.295	0.055	0.201	4.52E-91
RCN1	-0.295	0.525	0.681	2.49E-64
TUBB2B	-0.296	0.079	0.253	1.07E-103
THBS2	-0.307	0.374	0.518	1.34E-43
CDK6	-0.308	0.623	0.753	9.39E-61
PRSS2	-0.313	0.159	0.296	1.04E-49
PALD1	-0.322	0.208	0.421	3.66E-106
NKD1	-0.331	0.336	0.518	4.36E-73
CCND2	-0.348	0.445	0.596	3.08E-57
BTG2	-0.351	0.514	0.644	1.16E-49
RETNLB	-0.357	0.010	0.213	5.11E-194
PLCB4	-0.359	0.350	0.497	7.21E-58
CTNNB1	-0.364	0.684	0.805	1.96E-85
RGMB	-0.371	0.280	0.474	4.54E-87
ACAA2	-0.375	0.352	0.504	2.10E-63
SNHG14	-0.378	0.373	0.548	1.69E-76
FUT9	-0.385	0.355	0.558	6.16E-98
TUBA1A	-0.390	0.296	0.501	1.53E-91
TGFBI	-0.399	0.371	0.581	2.11E-103
ATF3	-0.401	0.419	0.558	2.96E-57
AMACR	-0.424	0.283	0.499	2.15E-105
ITPR2	-0.424	0.363	0.583	3.59E-113
LGR5	-0.460	0.328	0.558	3.33E-124
GSTM3	-0.511	0.289	0.602	1.15E-218
SMOC2	-0.533	0.752	0.865	7.47E-130
SLC38A11	-0.568	0.072	0.312	3.43E-183
VCAN	-0.663	0.098	0.366	2.32E-194
PRAC1	-0.722	0.176	0.606	0.00E+00
MTRNR2L8	-1.720	0.332	0.762	0.00E+00
PLA2G2A	-3.928	0.081	0.439	0.00E+00

KLHL24	0.261	0.434	0.288	2.25E-50
GSN	0.260	0.654	0.501	9.58E-55
PNRC1	0.257	0.707	0.578	2.44E-47
BCL2L1	0.256	0.525	0.397	1.30E-39
MYH9	0.252	0.799	0.697	4.93E-41
CD74	0.252	0.443	0.310	9.05E-43
DYNC1H1	0.252	0.708	0.598	1.92E-44
AC119673.2	0.251	0.201	0.031	2.55E-132
AURKAIP1	-0.251	0.541	0.659	1.05E-44
ANP32B	-0.253	0.609	0.729	1.65E-46
MRPL12	-0.253	0.262	0.412	2.71E-55
MZT2B	-0.256	0.602	0.735	2.25E-54
POMC	-0.259	0.016	0.194	4.55E-168
DCXR	-0.260	0.354	0.513	9.76E-56
FAM162A	-0.260	0.566	0.690	1.18E-37
ARL6IP4	-0.260	0.630	0.733	6.62E-51
CUTA	-0.260	0.530	0.647	1.63E-43
HIGD2A	-0.263	0.505	0.645	9.35E-51
HLA-E	-0.264	0.498	0.618	1.85E-43
PRELID1	-0.265	0.562	0.678	2.30E-46
CCT5	-0.268	0.482	0.607	1.01E-44
SH3BGRL3	-0.268	0.705	0.810	4.39E-46
ATP5MC1	-0.269	0.532	0.659	4.05E-45
TRMT112	-0.277	0.517	0.656	6.14E-57
PRDX6	-0.279	0.611	0.742	1.08E-62
S100P	-0.280	0.080	0.184	1.12E-43
CREB3L2	-0.284	0.234	0.387	1.77E-61
PEG10	-0.285	0.293	0.425	8.56E-40
METRN	-0.285	0.365	0.520	1.77E-60
GTF3A	-0.287	0.329	0.492	1.46E-63
SLCO1B3	-0.287	0.274	0.430	1.18E-57
GSTO1	-0.290	0.452	0.599	1.66E-59
PSMB9	-0.294	0.156	0.337	3.92E-91
PIGR	-0.297	0.215	0.376	3.47E-62
RNASE1	-0.298	0.434	0.592	7.55E-45
MGST1	-0.302	0.447	0.616	4.81E-66
RANBP1	-0.305	0.437	0.584	1.09E-55
TSPO	-0.311	0.734	0.850	1.28E-77
ATP5F1B	-0.315	0.704	0.807	3.03E-72
APRT	-0.319	0.669	0.783	2.30E-68
CYC1	-0.319	0.328	0.512	1.81E-81
NME1	-0.321	0.402	0.551	2.91E-61
EPB41L2	-0.321	0.482	0.647	5.92E-72
TMEM141	-0.324	0.432	0.621	6.14E-69
SNRPB	-0.330	0.609	0.734	1.28E-66
EIF5A	-0.334	0.542	0.683	7.11E-69
MDH2	-0.335	0.511	0.674	1.54E-86
EIF4EBP1	-0.335	0.296	0.485	2.12E-82
ECH1	-0.335	0.493	0.668	7.07E-86
COX5A	-0.340	0.743	0.852	2.84E-90
C1QBP	-0.351	0.562	0.693	7.72E-73
GUK1	-0.367	0.652	0.791	3.33E-96
CCDC85B	-0.381	0.423	0.618	1.46E-95
ZNF511	-0.383	0.447	0.643	1.42E-87
ENO1	-0.384	0.843	0.959	3.20E-116
HMGA1	-0.391	0.676	0.799	1.06E-97
RPL22L1	-0.396	0.481	0.649	1.57E-83
AQP5	-0.403	0.750	0.866	1.58E-68
UCA1	-0.414	0.344	0.493	1.05E-54
MTRNR2L1	-0.419	0.026	0.243	6.83E-203
NQO1	-0.431	0.376	0.609	7.52E-122
ATP5MC3	-0.436	0.808	0.910	4.62E-147
PRDX5	-0.446	0.617	0.799	7.50E-142
TKT	-0.450	0.788	0.908	2.95E-160
SLC14A1	-0.453	0.136	0.333	5.40E-104
CHCHD10	-0.461	0.387	0.651	2.08E-163
HLA-C	-0.461	0.830	0.932	1.68E-152
LDHA	-0.462	0.771	0.925	1.69E-123
SLC25A5	-0.496	0.761	0.895	1.14E-181
LGALS4	-0.517	0.752	0.904	1.52E-171
CDC42EP5	-0.521	0.240	0.500	1.46E-165
ATP5F1D	-0.567	0.756	0.900	1.10E-238
THBS2	-0.595	0.090	0.374	1.96E-226
AGR2	-0.628	0.656	0.871	2.20E-224
PAX8-AS1	-0.681	0.025	0.430	0.00E+00
IFI27	-0.799	0.147	0.440	3.31E-238
HLA-B	-0.843	0.775	0.952	0.00E+00
PLIN2	-0.937	0.674	0.843	6.41E-209
XIST	-1.073	0.010	0.384	0.00E+00
OLFM4	-1.238	0.184	0.607	0.00E+00

CCDC85B	-0.294	0.423	0.587	1.33E-64
EIF4EBP1	-0.295	0.296	0.486	6.22E-82
EIF5A	-0.298	0.542	0.687	2.84E-64
FABP5	-0.299	0.249	0.415	2.63E-68
ODC1	-0.304	0.368	0.552	8.30E-79
SATB2	-0.306	0.018	0.205	5.80E-180
CASC19	-0.309	0.330	0.530	3.17E-78
PDIA6	-0.310	0.792	0.895	1.70E-91
ATP5MC1	-0.320	0.532	0.695	1.16E-77
GTF3A	-0.323	0.329	0.524	1.63E-91
CHCHD10	-0.327	0.387	0.599	1.07E-101
S100P	-0.331	0.080	0.189	1.56E-50
PALD1	-0.331	0.205	0.421	3.31E-118
APRT	-0.331	0.669	0.799	3.70E-90
SLC25A5	-0.331	0.761	0.875	8.26E-94
PEG10	-0.334	0.293	0.485	1.89E-82
PRMT1	-0.335	0.455	0.638	4.70E-92
TMEM141	-0.340	0.432	0.618	1.71E-73
RETNLB	-0.344	0.020	0.213	2.89E-185
PA2G4	-0.346	0.569	0.700	3.73E-65
RCN1	-0.346	0.490	0.681	2.65E-91
PLCB4	-0.349	0.349	0.497	2.92E-61
TSPAN5	-0.362	0.463	0.670	6.10E-111
CCT5	-0.364	0.482	0.678	2.88E-103
ANOS1	-0.365	0.041	0.288	1.37E-226
PRSS23	-0.366	0.335	0.549	6.93E-112
SNU13	-0.371	0.581	0.739	1.02E-101
LGR5	-0.382	0.353	0.558	6.26E-96
CCND2	-0.382	0.367	0.596	2.66E-97
SNRPB	-0.389	0.609	0.763	2.37E-108
CKB	-0.398	0.233	0.437	1.42E-108
NME1	-0.399	0.402	0.606	5.84E-111
ATP5F1D	-0.400	0.756	0.875	5.57E-139
C1QBP	-0.409	0.562	0.742	3.24E-126
RANBP1	-0.413	0.437	0.642	1.64E-109
TKT	-0.416	0.788	0.904	1.12E-153
HLA-B	-0.419	0.775	0.901	3.91E-127
PRDX5	-0.420	0.617	0.800	9.80E-144
LDHA	-0.439	0.771	0.883	1.11E-91
PPIB	-0.455	0.814	0.918	4.34E-172
RPL22L1	-0.459	0.481	0.675	1.34E-121
EPB41L2	-0.475	0.482	0.722	6.35E-171
GABPB1-AS1	-0.501	0.468	0.693	9.26E-155
MTRNR2L1	-0.513	0.026	0.304	7.05E-286
VCAN	-0.539	0.136	0.366	4.75E-144
TGFBI	-0.555	0.275	0.581	2.16E-218
PLIN2	-0.566	0.674	0.788	9.07E-83
AGR2	-0.612	0.656	0.877	5.08E-243
THBS2	-0.903	0.090	0.518	0.00E+00
XIST	-1.514	0.010	0.465	0.00E+00
MTRNR2L8	-1.550	0.426	0.762	0.00E+00
PLA2G2A	-3.520	0.149	0.439	3.12E-267

Supplementary Table 5 Antibody panel for IMC

Mass	Metal	Marker	Clone	Vendor	Dilution
141	Pr	a-SMA	1A4	Fluidigm	1000
142	Nd	Mucin 2	Ccp58	Santa Cruz	100
143	Nd	Vimentin	RV202	Fluidigm	400
144	Nd	CD14	EPR3653	Fluidigm	100
145	Nd	BCL-6	LN22	lovus Biologica	25
146	Nd	CD16	EPR16784	Fluidigm	100
148	Nd	Pan Keratin	C11	Fluidigm	300
149	Sm	CD11b	EPR1344	Fluidigm	200
151	Eu	CD11c	EP1347Y	Abcam	200
152	Sm	CD45	2B11	Fluidigm	200
153	Eu	CD56	Rabbit Poly Ab	Proteintech	300
154	Sm	IL-6	3154011B	Fluidigm	100
155	Gd	Foxp3	236A/E7	Fludigim	75
156	Gd	CD4	EPR6855	Fluidigm	100
158	Gd	E-Cadherin	2.40E+10	Fluidigm	300
159	Tb	CD68	KP1	Fluidigm	600
160	Gd	Lysozyme C	E5	Santa Cruz	200
161	Dy	CD20	H1	Fluidigm	200
162	Dy	CD8a	C8/144B	Fluidigm	300
163	Dy	CXCR3	G025H7	Fluidigm	300
165	Ho	CD69	sc-373798	Santa Cruz	200
166	Ho	NFKB	3166006A	Fluidgim	100
167	ER	Granzyme B	EPR20129-217	Fluidigm	200
168	Er	Ki67	B56	Fluidigm	600
169	Tm	Collagen I	Polyclonal	Fluidigm	600
170	ER	CD3	Polyclonal (C terminal)	Fluidigm	200
171	Yb	CD27	EPR8569	Fluidigm	100
173	Yb	CD45RO	UCHL1	Fluidigm	300
174	Yb	HLA-DR	TAL 1B5	Abcam	600
175	Lu	CD38	H-11	Santa Cruz	100
176	Ho	Histone	D1H2	Fluidigm	1000
191/193	Ir	DNA	--	Fluidigm	400

Detailed information on image acquired by IMC

Subject ID	Slide ID	Condition	Region	Image ID	Acquisition area (ums)	Total no. cells captured per image	No. cells per image, after filtering	No. cells per sample, after filtering
Variant	SUR 5385	variant	Right colon	Variant_ROI 001	2000 x 1100	15514	5795	11011
				Variant_ROI 002	2000 x 2000	22585	5216	
VEO-IBD-1	SUR 2644	VEO IBD	Right colon	VEO-IBD-1_ROI001	1100 x 3500	18780	15544	15544
VEO-IBD-2	SUR 2956	VEO IBD	Colon	VEO-IBD-2_ROI001	600 x 2000	6198	6198	15984
				VEO-IBD-2_ROI002	1400 x 1300	9790	9786	
VEO-IBD-3	SUR 5419	VEO IBD	Colon	VEO-IBD-3_ROI002	1000 x 2000	12867	12764	25129
				VEO-IBD-3_ROI003	900 x 2000	12365	12365	
Control-4	SUR 6941	Healthy	Colon	Control-4_ROI003	900 x 1750	5721	5083	12468
				Control-4_ROI004	350 x 2500	4551	4545	
				Control-4_ROI005	600 x 1000	3066	2840	
Control-3	S15 9148	Healthy	Left colon	Control-3_ROI001	1000 x 2884	10051	6767	6767
Control-2	SUR 3497	Healthy	Left colon	Control-2_ROI001	800 x 2500	9353	9108	11917
				Control-2_ROI002	500 x 1500	2809	2809	
