# Supplementary material for: TNFSF13 insufficiency disrupts human colonic epithelial cell growth and associated B cell dynamics
Source: J Clin Invest. 2026 Apr 1;136(7):e186032. doi: 10.1172/JCI186032 (PMC13038214; doi:10.1172/JCI186032)

Full unedited blot/gel for
Fig 2C

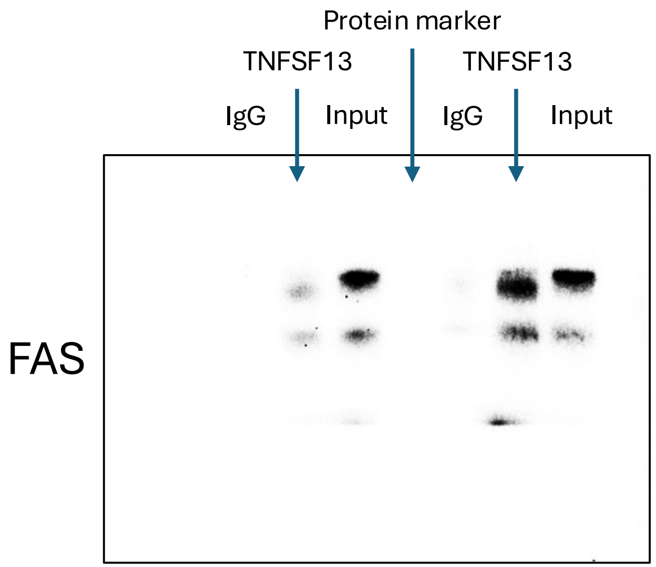

Full unedited blot/gel for
Fig S7B upper

b-ACTIN

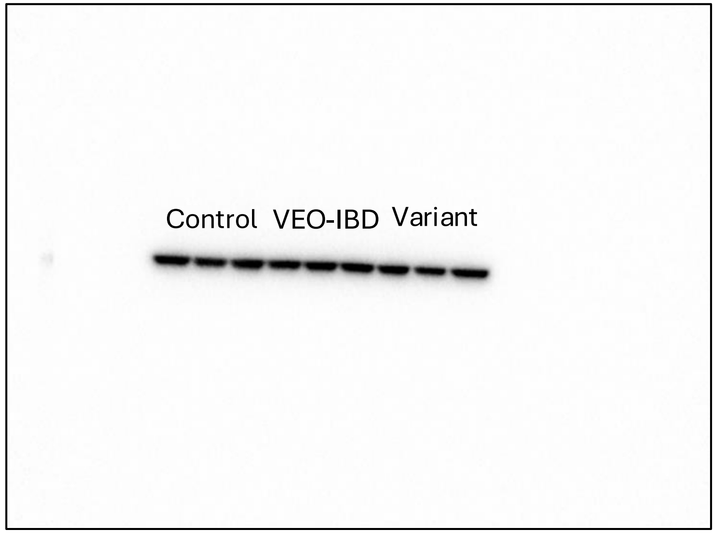

BCL-XL

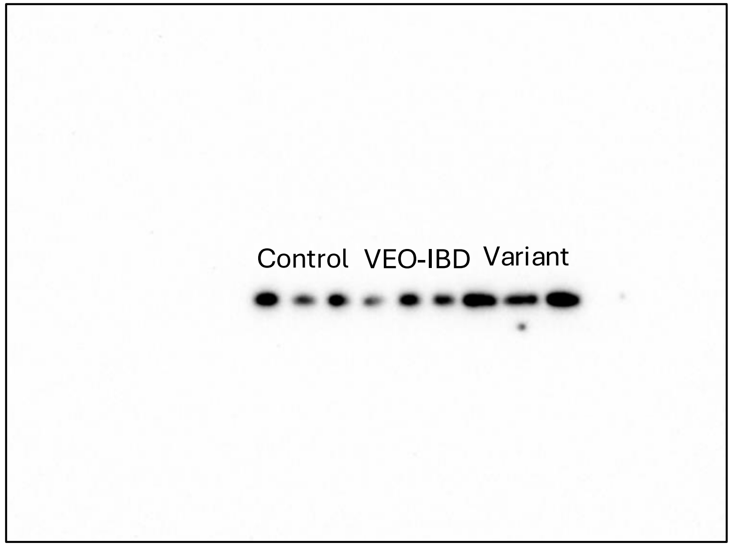

Full unedited blot/gel for
Fig S7B lower

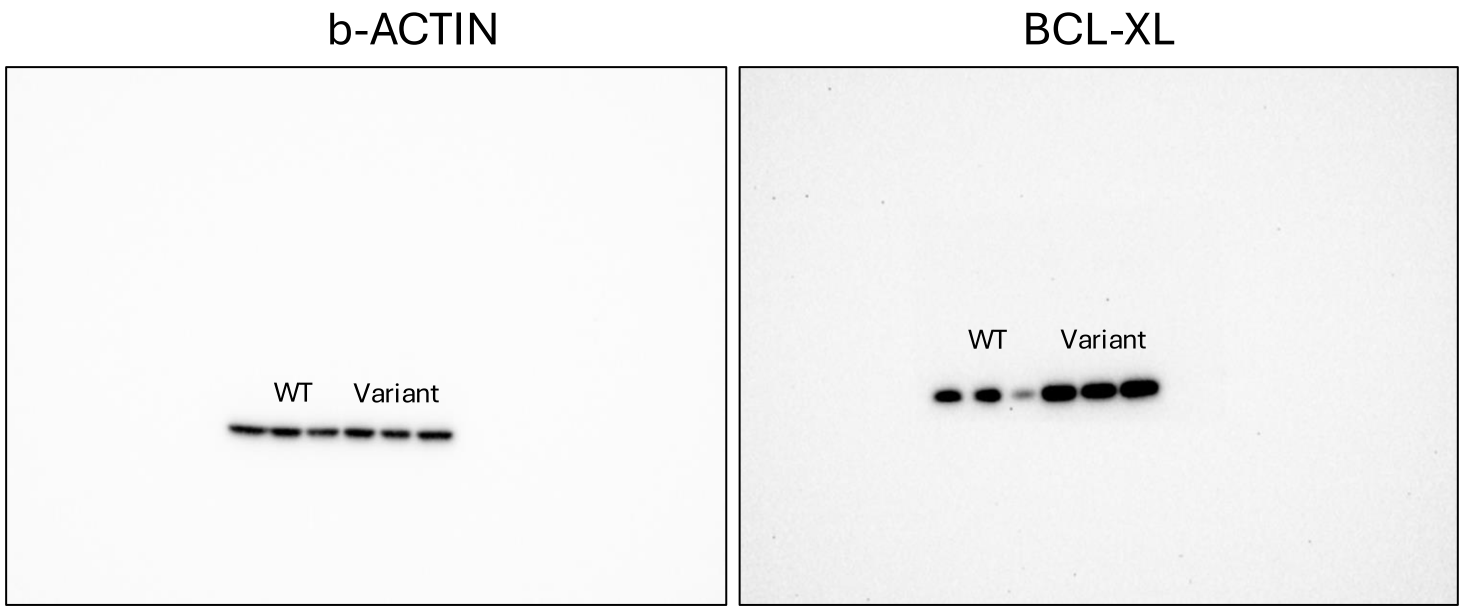

Full unedited blot/gel for
Fig S7C upper

Vinculin

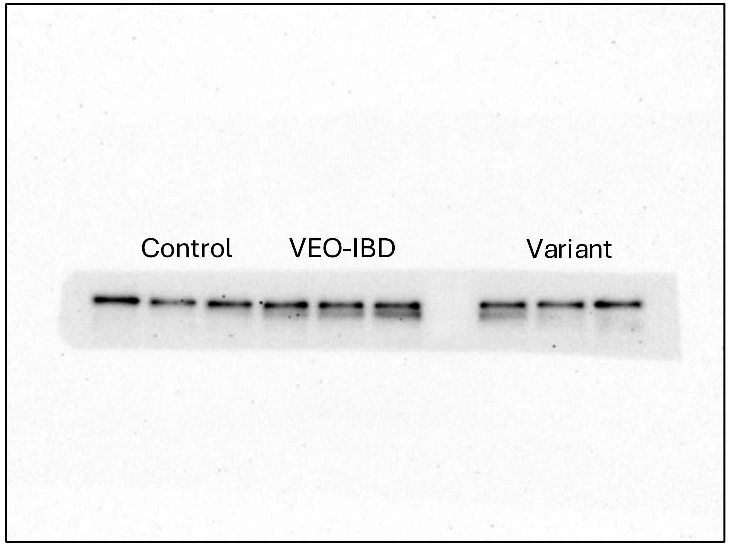

Cleaved capase3

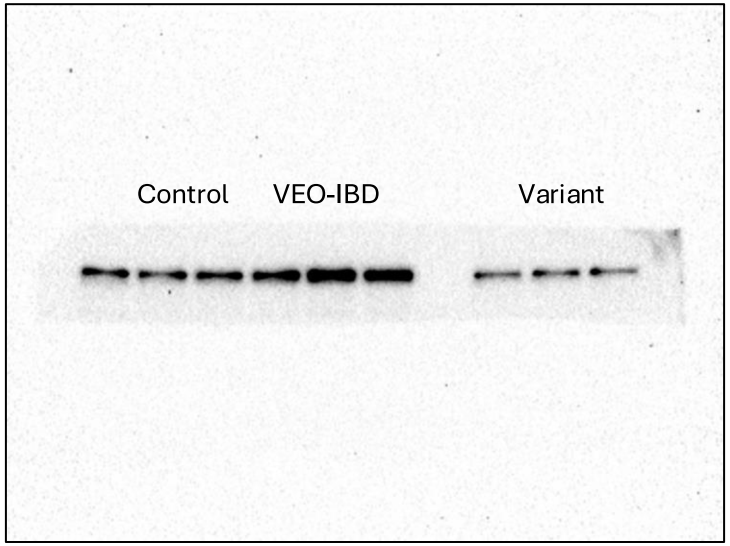

Full unedited blot/gel for
Fig S7B lower

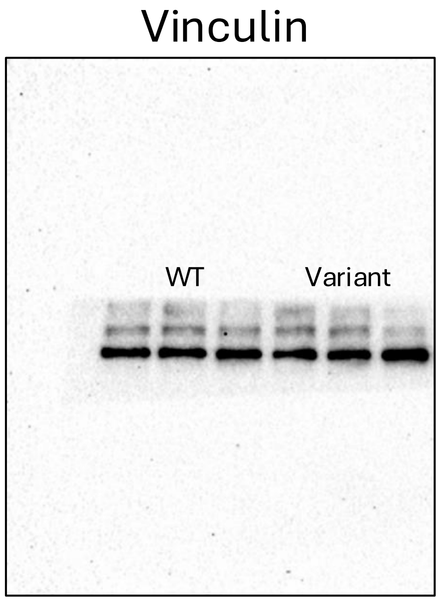

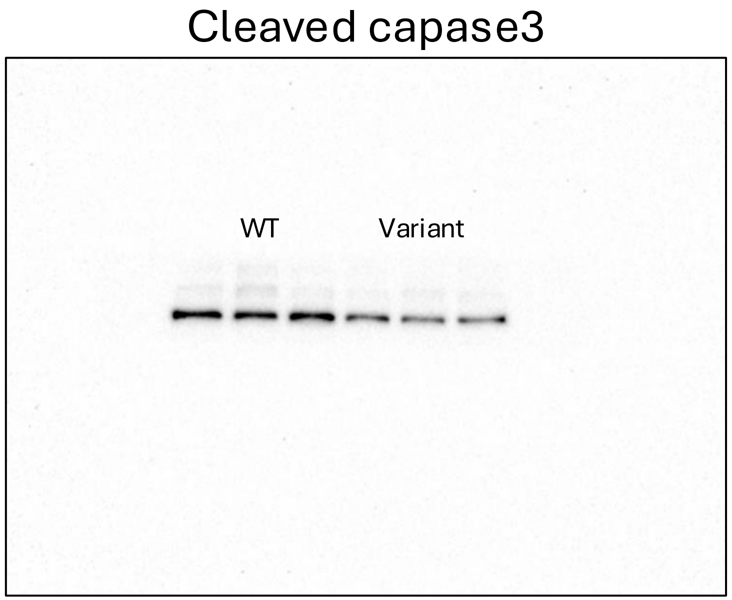

Full unedited blot/gel for
Fig S7D

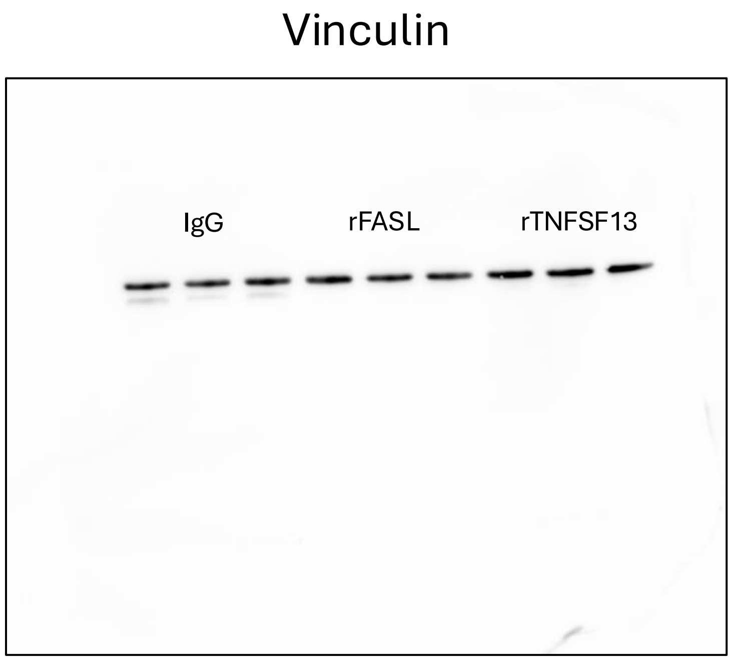

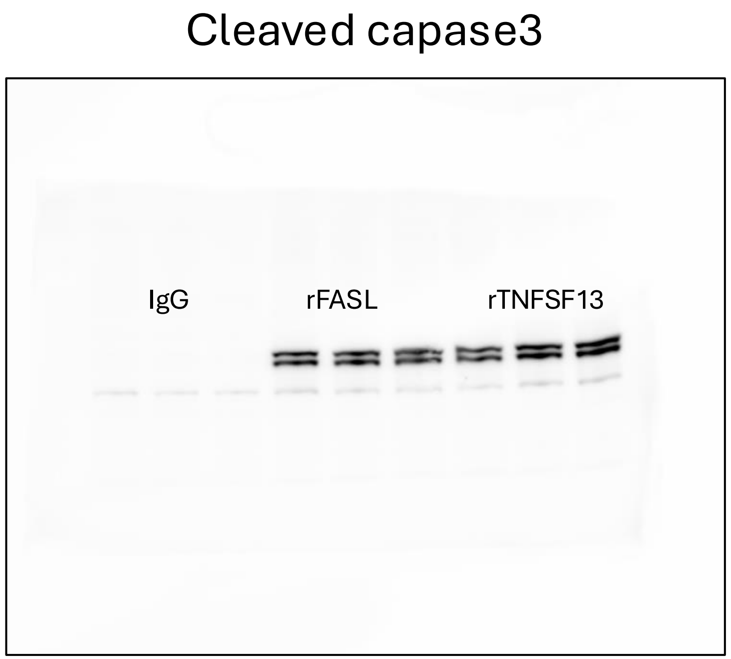

Full unedited blot/gel for
Fig S7D

Vinculin

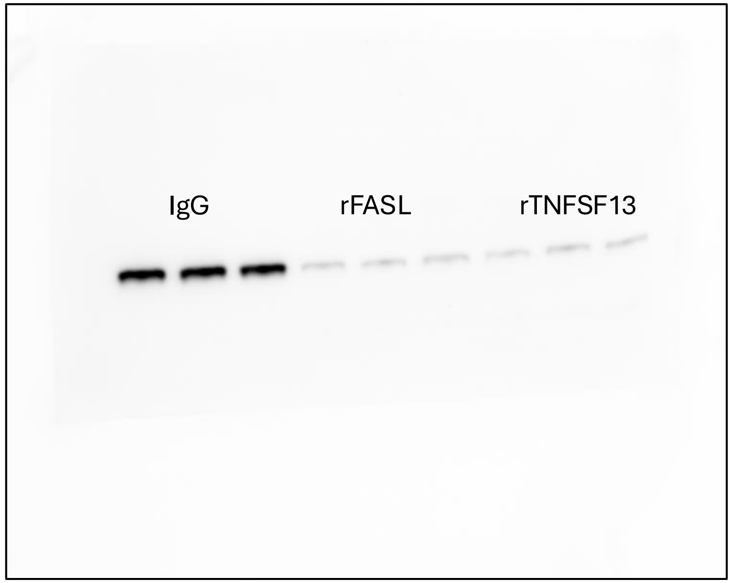

Cleaved capase3

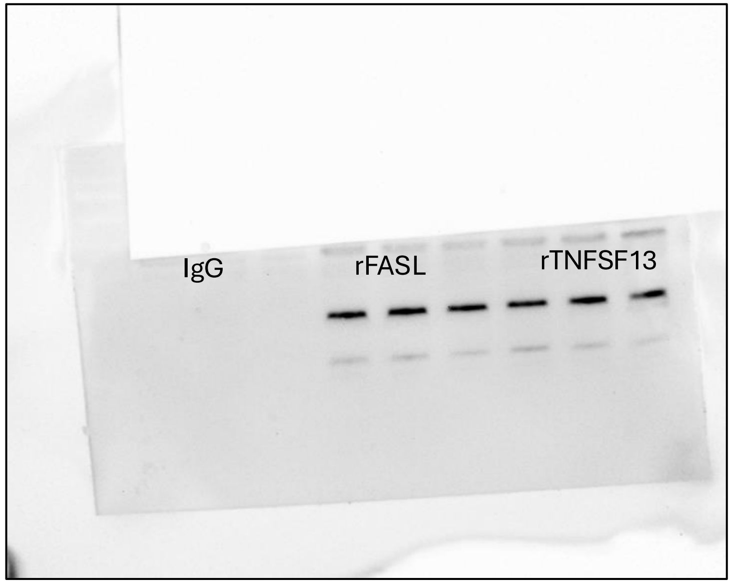

BCL-xL

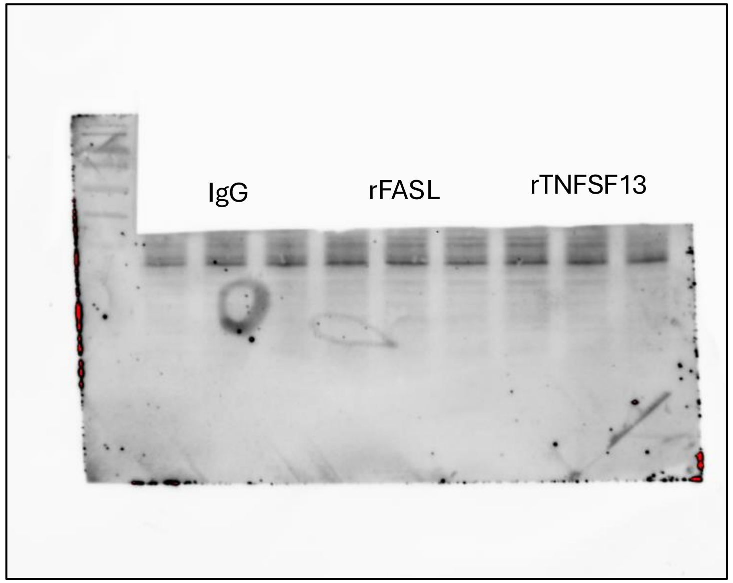

Supplement: Unedited blot and gel images [file jci-136-186032-s268.pdf]
